# Cyanothioacetamides as a synthetic platform for the synthesis of aminopyrazole derivatives

**DOI:** 10.3762/bjoc.19.87

**Published:** 2023-08-08

**Authors:** Valeriy O Filimonov, Alexandra I Topchiy, Vladimir G Ilkin, Tetyana V Beryozkina, Vasiliy A Bakulev

**Affiliations:** 1 Technology of Organic Synthesis Department, Ural Federal University named after the first President of Russia B. N. Yeltsin, 19 Mira st. Yekaterinburg 620002, Russia; 2 Department of Organic Chemistry, Perm State University, 15 Bukireva st., Perm 614990, Russia

**Keywords:** aminopyrazoles, 2-cyanothioacetamides, enamines, hydrazines

## Abstract

It was shown that the reaction of 2-cyanothioacetamides with hydrazine involves both cyano- and thioamide groups, and 3,5-diaminopyrazoles are formed. In the reaction of 2-cyano-3-(dimethylamino)-*N*,*N*-dimethylprop-2-enethioamides with hydrazine and its derivatives, the interaction proceeds with the participation of cyano- and enamine groups, not affecting the thiocarbamoyl group, and leads to the formation of 4-thiocarbamoylpyrazoles. A synthesis method has been developed and a series of 1-substituted-4-thiocarbamoyl pyrazoles has been thus synthesized. The structure of the reaction products was studied using NMR spectroscopy and mass spectrometry and confirmed by X-ray diffraction analysis.

## Introduction

**Figure 1:**
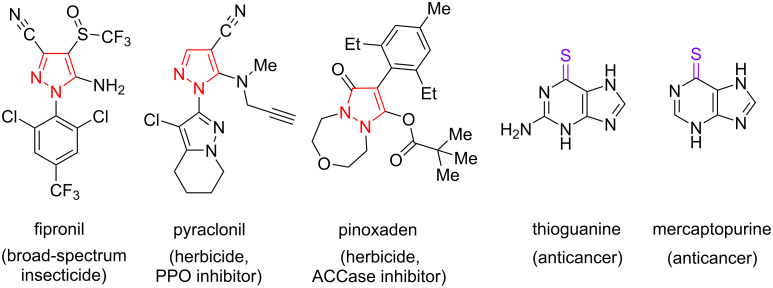
Examples of bioactive pyrazoles for the protection of cultivated plants and drugs containing a thiocarbonyl group.

Compounds containing a pyrazole cycle exhibit a variety of biological activity. Their application is known in pharmacy [1–4] and agro-industry [5–8]. Especially such compounds are used to protect plants from insects and weeds (Figure 1).

Thus, pinoxaden is a commercially available inhibitor of acetyl-CoA carboxylase and affects the biosynthesis of fatty acids in plants, which leads to herbicidal activity [9]. Pyraclonil is used as a protoporphyrinogen oxidase inhibitor for weed control. Such inhibitors not only block the production of chlorophyll and gem in plant pests, but also lead to the formation of highly reactive molecules that attack and destroy lipids and protein membranes of weed cells [10]. Fipronil is an inhibitor of GABA receptors and affects the nervous system of insects as a broad-spectrum insecticide [11–12].

On the other hand, thioamides are an important class of organic compounds. 6-Thioguanine and mercaptopurine (Figure 1), containing an intracyclic thioamide group, are antagonists of purine bases and are well known as cytostatic drugs [13]. The thioamide group as an amide isostere is used in medical chemistry to increase thermal and proteolytic stability and improve the pharmacokinetic properties of biologically active substances containing amide groups [14].

The presence of a pyrazole core and a thioamide group within the hybrid molecules that we are planning to obtain, allows us to expect both an increase in their activity and the emergence of other types of biological activity, and also high synthetic potential of such compounds.

**Scheme 1:**
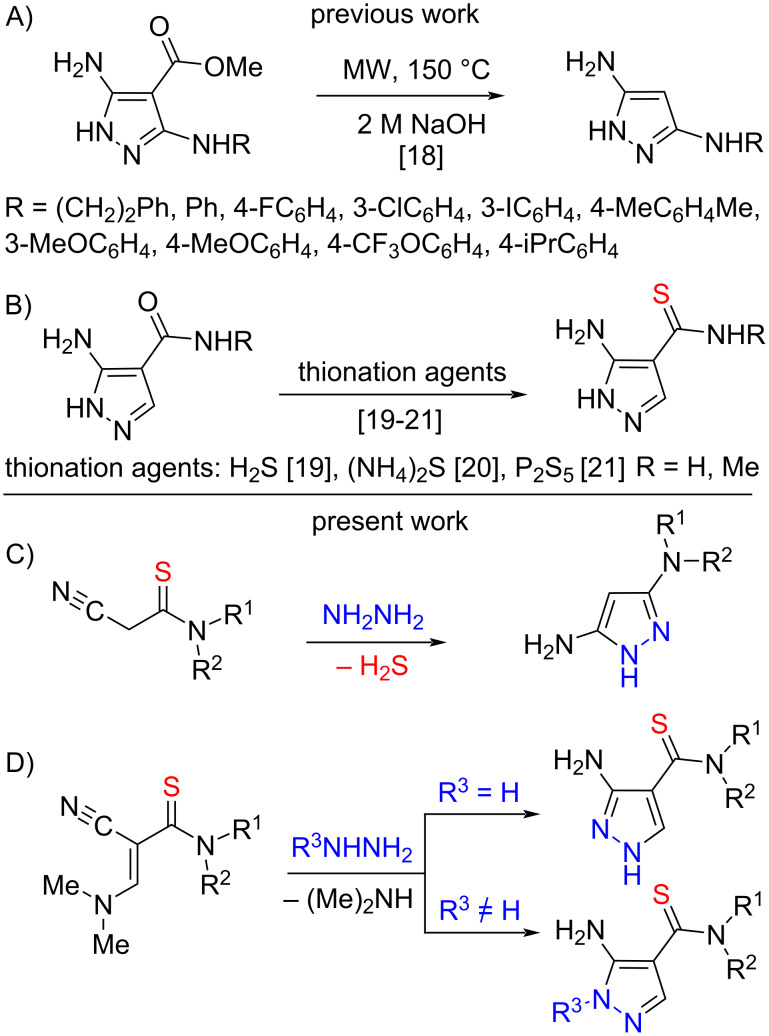
Syntheses of 4,5-diamino- and 4-thiocarbamoyl-5-aminopyrazoles.

The most common method for obtaining 3,5-disubstituted pyrazoles is the cyclocondensation of 1,3-bielectrophilic reagents with hydrazine, which acts as a 1,2-dinucleophile [15–16]. It is worthy to note that a small number of methods for the synthesis of 3,5-diaminopyrazoles are presented in the literature. These syntheses are multistage [17] or are essentially a transformation of the structure of the previously obtained pyrazoles [18] (Scheme 1A).

Methods for the synthesis of pyrazoles containing thioamide and amino groups in a molecule are even less developed than methods for obtaining 3,5-diaminopyrazoles, and presented in the literature by two examples only [19–21] (Scheme 1B).

Thus, the development of effective methods for the production of 3,5-diaminopyrazoles and 3,5-diaminothiocarbamoylpyrazoles is an actual task which was chosen the aim of current study. Here, we first present the formation of 3,5-diaminopyrazoles by reaction of 2-cyanothioacetamides with hydrazine (Scheme 1C). Our paper also contains the data on our study of reaction of 3-amino-2-cyanoprop-2-enethioamides with phenyl- and tosylhydrazines leading to novel 3-amino-4-thiocarbamoylpyrazoles (Scheme 1D).

## Results and Discussion

**Figure 2:**
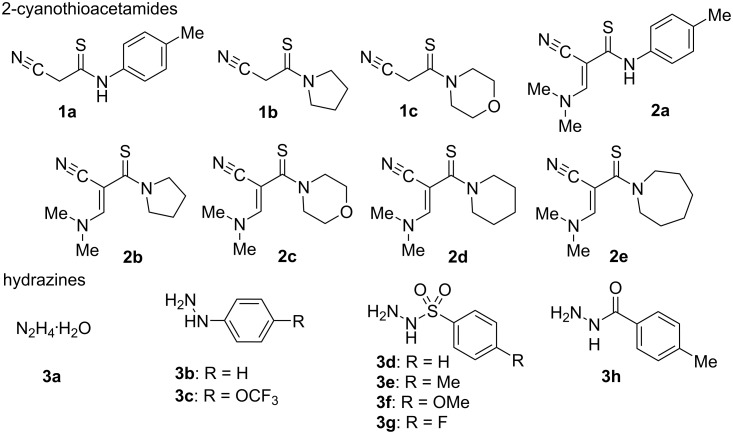
Structures of starting materials.

Considering that the construction of the pyrazole cycle can be carried out by the interaction of hydrazine with 1,3-bielectrophilic reagents, we paid attention to the structure of 2-cyanothioacetamides **1** and 3-amino-2-cyanoprop-2-enethioamides **2** [22] which combine in one molecule cyano- and thioamide groups, as well as a fragment of enamine, each in principle being capable of interaction with hydrazine (**3a**) (Figure 2).

**Scheme 2:**
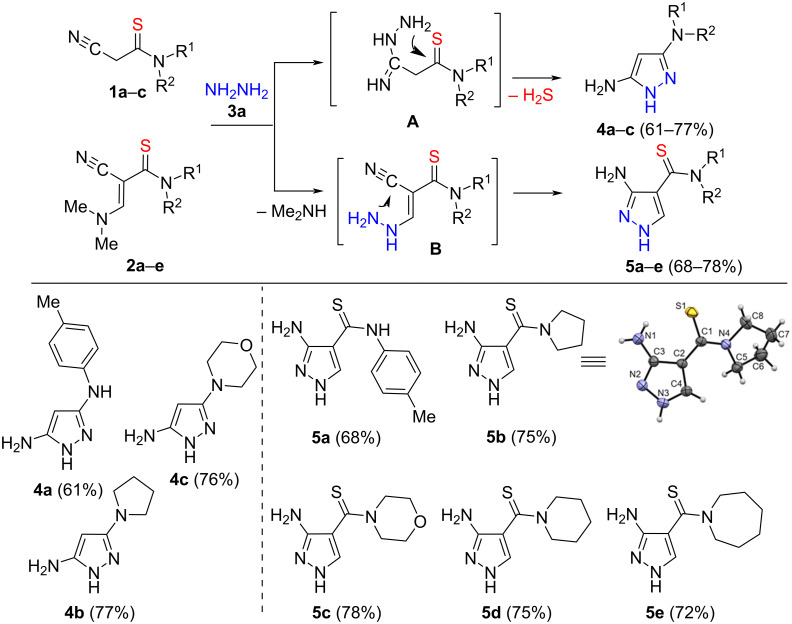
Synthesis of 3,5-diaminopyrazoles **4а**–**с** and thiocarbamoyl-*NH*-pyrazoles **5a–e**. Reaction conditions: **1a**–**c** or **2a**–**e** (1 mmol), **3a** (80% aq solution) (1.1 mmol), EtOH, 80 °С, 4‒6 h.

First, we have studied the reaction of thioamides **1a**–**c** with hydrazine (**3a**). It was found that the reaction proceeds smoothly in ethanol at 80 °C to form 3,5-diaminopyrazoles **4a**–**c** (Scheme 2).

The structures of compounds **4a**–**c** were confirmed by ^1^H and ^13^C NMR spectroscopy data, as well as high-resolution mass spectrometry (HRMS). Compound **4a** was previously obtained by another method [18]. The spectral characteristics of diaminopyrazole **4a** reported in [18] correspond to the data given in the current work.

The formation of 3,5-diaminopyrazoles **4a**–**c** occurs, presumably, as a result of a sequential attack of electrophilic carbon atoms of the cyano- and thioamide groups of thioamides **1a**–**c** by nucleophilic nitrogen atoms of hydrazine (**3a**) and is accompanied by the elimination of hydrogen sulfide (Scheme 2).

Thus, we have shown that when 2-cyanothioacetamides **1a**–**c** react with hydrazine hydrate (**3a**) in ethanol, both groups (thioamide and cyano) interact with hydrazine with the elimination of hydrogen sulfide and the formation of 3,5-diaminopyrazoles **4a**–**c** (Scheme 2). It should be noted that such a reaction has not been described in the literature so far.

On the contrary, the reaction of thioamides **2a**–**e** with hydrazine (**3a**) does not affect the thioamide group, and only the enamine and cyano groups participate in the construction of the pyrazole cycle. Thus, the desired 4(3)-thiocarbamoyl-*NH*-pyrazoles **5a**–**e** were obtained with a yield of 68–78% (Scheme 2).

These experiments allowed us to conclude that in the compounds **2a**–**e** under study, the enamine group has a higher reactivity towards hydrazine than the thioamide one, which leads to the preservation of the thioamide group during the reaction.

The structures of compounds **5a**–**e** were confirmed by ^1^H and ^13^C NMR spectroscopy and HRMS, as well as X-ray diffraction analysis of a single crystal of compound **5b**.

**Scheme 3:**
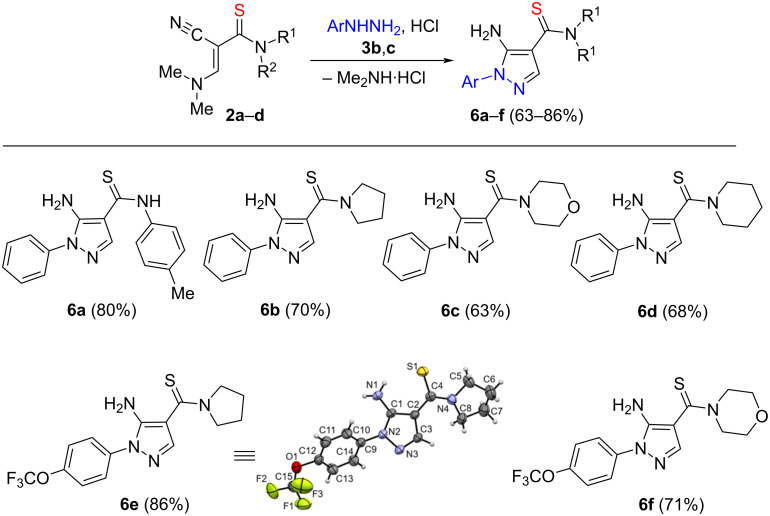
Synthesis of 1-aryl-5-amino-4-thiocarbamoylpyrazoles **6a**–**f**. Reaction conditions: **2a**–**d** (1 mmol), **3b**,**c** (1.1 mmol), aq HCl (1.1 mmol), EtOH, 80 °С, 12‒16 h.

The involvement of arylhydrazines **3b**,**c** in the reaction with enamines **2a**–**d** similarly leads to the formation of 1-aryl-5-amino-4-thiocarbamoyl pyrazoles **6a**–**f** with yields of 63–86% (Scheme 3).

However, we have found that when replacing hydrazine hydrate with arylhydrazines and arylsulfonylhydrazines, the reaction in ethanol was not detected, neither at room temperature nor at reflux. The progress was achieved only upon addition of hydrochloric acid. This is probably due to the protonation of the dimethylamino moiety or/and that dimethylamine hydrochloride is a better leaving group than the free base.

The structures of compounds **6a**–**f** were confirmed by ^1^H and ^13^C NMR spectroscopy and HRMS, as well as X-ray diffraction analysis of a single crystal of compound **6e**.

**Scheme 4:**
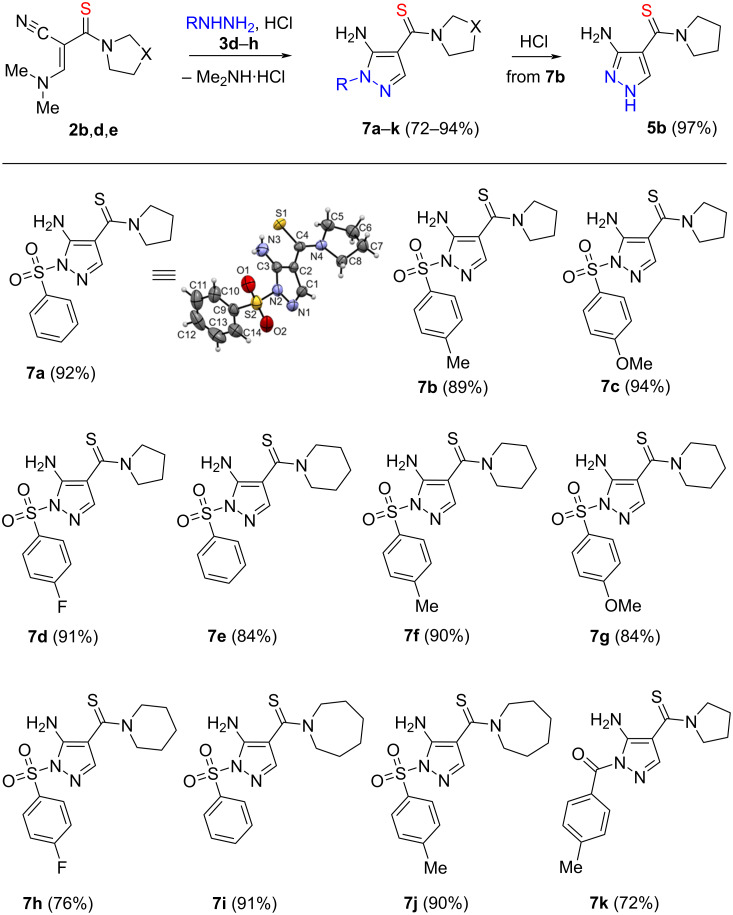
Synthesis of 1-sulfonylpyrazoles **7a**–**j** and 1-benzoylpyrazole **7k**. Conditions for **7a**–**j**: **2b**,**d**,**e** (1 mmol), **3d**–**g** (1.1 mmol), aq HCl (1.1 mmol), EtOH, rt, 12‒16 h. Conditions for **7k**: **2b** (1 mmol), **3h** (1.1 mmol), aq HCl (1.1 mmol), EtOH, 60 °C, 5 h. Conditions for **5b**: **7b** (1 mmol), aq HCl (1 mmol), EtOH, 80 °C, 24 h.

It is interesting to note that the replacement of hydrazine (**3a**) with arylsulfonylhydrazines **3d**–**g** and benzoylhydrazine (**3h**) in the reaction with enamines **2b**,**d** leads to the formation of 1-sulfonylpyrazoles **7a**–**j** and 1-benzoylpyrazole **7k** with a significantly higher yield (72–94%, Scheme 4).

We have noticed that 1-sulfonylpyrazole **7b** is unstable when heated in ethanol in the presence of hydrochloric acid and converts into 3-aminopyrazole-4-carbothioamide **5b**. It is worth noting that the two-stage method of obtaining 3-aminopyrazole-4-carbothioamide **5b** described above is more preferable (Scheme 4) than the one-stage method using hydrazine **3a** (Scheme 2), (yields 89 × 97 = 86% and 75%, respectively).

## Conclusion

In order to develop an effective method for the synthesis of functional derivatives of pyrazoles, the reactions of 2-cyanothioacetamides and their derivatives, 3-amino-2-cyanoprop-2-enethioamides, with hydrazine, (substituted)phenyl- and (substituted)phenylsulfonylhydrazines were studied. The reaction between these compounds proceeds smoothly in ethanol to form 3,4-diaminopyrazoles, 5-amino-4-thiocarbamoylpyrazoles, 1-(substituted)phenyl- or 1-(substituted)phenylsulfonyl-4-thiocarbamoylpyrazoles in moderate to high yields. It was concluded that in 2-cyanothioacetamides, cyano and enamino groups are more active in the reaction with hydrazines than the thiocarbamoyl function.

## Experimental

### X-ray structure determination of **5b**, **6a**, **7a**

**5b**: Crystal data for C_8_H_12_N_4_S (*M* = 196.27 g/mol): monoclinic, space group *P*2_1_/*c* (no. 14), *a* = 10.449(2) Å, *b* = 9.6365(17) Å, *c* = 9.406(2) Å, β = 97.10(2)°, *V* = 939.8(3) Å^3^, *Z* = 4, μ(Mo Kα) = 0.302 mm^−1^, *D*_calc_ = 1.387 g/cm^3^, 4629 reflections measured (5.736° ≤ 2Θ ≤ 58.142°), 2229 unique (*R*_int_ = 0.0315, *R*_sigma_ = 0.0388) which were used in all calculations. The final *R*_1_ = 0.0403, w*R*_2_ = 0.0956 (*I* > 2σ(I)) and *R*_1_ = 0.0510, w*R*_2_ = 0.1042 (all data). Largest diff. peak/hole 0.281/−0.285 ēÅ^−3^.

**6e**: Crystal data for C_15_H_15_F_3_N_4_OS (*M* = 356.37 g/mol): triclinic, space group *P*-1 (no. 2), *a* = 6.1592(10) Å, *b* = 12.0568(18) Å, *c* = 12.1206(19) Å, α = 109.032 (14)°, β = 101.540 (13)°, γ = 100.648 (13), *V* = 802.7(2) Å^3^, *Z* = 2, μ(Mo Kα) = 0.244 mm^−1^, *D*_calc_ = 1.474 g/cm^3^, 5870 reflections measured (6.154° ≤ 2Θ ≤ 58.47°), 3676 unique (*R*_int_ = 0.0326, *R*_sigma_ = 0.0537) were used in all calculations. The final *R*_1_ = 0.0513, w*R*_2_ = 0.1241 (*I* > 2σ(I)) and *R*_1_ = 0.0685, w*R*_2_ = 0.1431 (all data). Largest diff. peak/hole 0.256/−0.392 ēÅ^−3^.

**7a**: Crystal data for C_14_H_16_N_4_O_2_S_2_ (*M* = 336.43 g/mol): monoclinic, space group *P*2_1_/*c* (no. 14), *a* = 15.431(4) Å, *b* = 10.307(3) Å, *c* = 9.946(2) Å, β = 103.45(2)°, *V* = 1538.5(7) Å^3^, *Z* = 4, μ(Mo Kα) = 0.358 mm^−1^, *D*_calc_ = 1.452 g/cm^3^, 7847 reflections measured (4.794° ≤ 2Θ ≤ 58.972°), 3663 unique (*R*_int_ = 0.0461, *R*_sigma_ = 0.0641) were used in all calculations. The final *R*_1_ = 0.0580, w*R*_2_ = 0.1295 (*I* > 2σ(I)) and *R*_1_ = 0.0993, w*R*_2_ = 0.1673(all data). Largest diff. peak/hole 0.244/−0.450 ēÅ^−3^.

The experiments were accomplished on the automated X-ray diffractometer «Xcalibur 3» with CCD detector following standard procedures (Mo Kα-irradiation, graphite monochromator, ω-scans with 1^o^ step at *T* = 295(2) K). Empirical absorption correction was applied. The structure was solved using the intrinsic phases in ShelXT program [23] and refined by ShelXL [24] using full-matrix least-squared method for non-hydrogen atoms. The H-atoms were placed in the calculated positions and were refined in isotropic approximation. The solution and refinement of the structures were accomplished with the Olex program package [25].

CCDC 2250448 (**5b**), CCDC 2250451 (**6e**) and CCDC 2250453 (**7a**) contains the supplementary crystallographic data for this paper. These data can be obtained free of charge via http://www.ccdc.cam.ac.uk/conts/retrieving.html (or from the CCDC, 12 Union Road, Cambridge CB2 1EZ, UK; Fax: +44 1223 336033; E-mail: deposit@ccdc.cam.ac.uk).

## Supporting Information

File 1 Full experimental details and characterization data of all new compounds.

File 2 Copies of NMR spectra of all new compounds.

## References

[1] Dadiboyena S, Nefzi A (2011). Eur J Med Chem.

[2] Mahesh P, Akshinthala P, Katari N K, Gupta L K, Panwar D, Sharma M K, Jonnalagadda S B, Gundla R (2023). ACS Omega.

[3] Sabnis R W (2023). ACS Med Chem Lett.

[4] Mykhailiuk P K (2021). Chem Rev.

[5] Kang J, Yue X L, Chen C S, Li J H, Ma H J (2016). Molecules.

[6] Dong L, Chang W, Yang W, Xu Z, Cheng J, Shao X, Xu X, Li Z (2023). J Agric Food Chem.

[7] Ansari A, Ali A, Asif M, Shamsuzzaman (2017). New J Chem.

[8] Ebenezer O, Shapi M, Tuszynski J A (2022). Biomedicines.

[9] Takano H K, Ovejero R F L, Belchior G G, Maymone G P L, Dayan F E (2021). Sci Agric (Piracicaba, Braz).

[10] Deng W, Yang Q, Chen Y, Yang M, Xia Z, Zhu J, Chen Y, Cai J, Yuan S (2020). J Agric Food Chem.

[11] Das P C, Cao Y, Cherrington N, Hodgson E, Rose R L (2006). Chem-Biol Interact.

[12] Narahashi T, Zhao X, Ikeda T, Salgado V L, Yeh J Z (2010). Pestic Biochem Physiol.

[13] Crouwel F, Buiter H J C, de Boer N K (2022). J Crohn's Colitis.

[14] Dyachenko V D, Dyachenko I V, Nenajdenko V G (2018). Russ Chem Rev.

[15] Fustero S, Sánchez-Roselló M, Barrio P, Simón-Fuentes A (2011). Chem Rev.

[16] Fustero S, Simón-Fuentes A, Sanz-Cervera J F (2009). Org Prep Proced Int.

[17] Gopalsamy A, Ciszewski G, Shi M, Berger D, Hu Y, Lee F, Feldberg L, Frommer E, Kim S, Collins K (2009). Bioorg Med Chem Lett.

[18] Lim F P L, Tan K C, Luna G, Tiekink E R T, Dolzhenko A V (2019). Tetrahedron.

[19] Bovara R, Largaiolli R, Meroni G (1971). J Labelled Compd.

[20] Khosropour A R, Noei J, Mirjafari A (2010). J Iran Chem Soc.

[21] Naito Y, Akahoshi F, Takeda S, Okada T, Kajii M, Nishimura H, Sugiura M, Fukaya C, Kagitani Y (1996). J Med Chem.

[22] Lugovik K I, Eltyshev A K, Benassi E, Belskaya N P (2017). Chem – Asian J.

[23] Sheldrick G M (2015). Acta Crystallogr, Sect A: Found Adv.

[24] Sheldrick G M (2015). Acta Crystallogr, Sect C: Struct Chem.

[25] Dolomanov O V, Bourhis L J, Gildea R J, Howard J A K, Puschmann H (2009). J Appl Crystallogr.

