# Supplementary material for: Cyanothioacetamides as a synthetic platform for the synthesis of aminopyrazole derivatives
Source: Beilstein J Org Chem. 2023 Aug 8;19:1191–7. doi: 10.3762/bjoc.19.87 (PMC10428587; doi:10.3762/bjoc.19.87)
Supplement: File 1 — Full experimental details and characterization data of all new compounds. [file Beilstein_J_Org_Chem-19-1191-s001.pdf]

BEILSTEIN JOURNAL OF ORGANIC CHEMISTRY

Supporting Information

for

Cyanothioacetamides as a synthetic platform for the synthesis of aminopyrazole derivatives

Valeriy O. Filimonov, Alexandra I. Topchiy, Vladimir G. Ilkin, Tetyana V. Beryozkina and Vasiliy A. Bakulev

Beilstein J. Org. Chem. **2023,** *19,* 1191–1197. doi:10.3762/bjoc.19.87

Full experimental details and characterization data of all new compounds

License and Terms: This is a supporting information file under the terms of the Creative Commons Attribution License (https://creativecommons.org/licenses/by/4.0). Please note that the reuse, redistribution and reproduction in particular requires that the author(s) and source are credited and that individual graphics may be subject to special legal provisions.
The license is subject to the *Beilstein Journal of Organic Chemistry* terms and conditions: (https://www.beilstein-journals.org/bjoc/terms)

Experimental part

General

All chemicals were purchased from commercial sources and were used without further purification. Thioamides **1a** [1], **1b**,**c** [2] and **2b**–**d** [3] were obtained as previously described. The reaction progress and purity of the obtained compounds were controlled by TLC method on Sorbfil UV-254 plates (Imid, Krasnodar, Russia), with visualization under UV light. Flash column chromatography was performed using KSKG GOST 3956-76 silica gel (0.063–0.125 mm) (ChromLab, Lyubertsy, Russia). Melting points were determined on a melting point apparatus Stuart SMP10 (Cole-Parmer Ltd, Staffordshire, UK) and are uncorrected. All NMR spectra were recorded with a Bruker Avance II spectrometer (Karlsruhe, Germany) at 400 MHz, (^{1}H NMR), 101 MHz (^{13}C NMR) and 376 MHz (^{19}F NMR) in $CDCl_3$ and DMSO–d_6. The chemical shifts are given in ppm relative to the resonance of the residual solvents (^{1}H: δ ($CHCl_3$) = 7.26 ppm, ^{13}C: δ ($CHCl_3$) = 77.16 ppm; ^{1}H: δ (DMSO–d_5) = 2.50 ppm, ^{13}C: δ (DMSO–d_5) = 39.52 ppm). Multiplicities were given as: s (singlet); br. s (broad singlet); d (doublet); t (triplet); dd (double of doublets); q (quadruplet); m (multiplet). High-resolution mass spectra (HRMS) were recorded using ultrahigh resolution quadrupole time-of-flight mass spectrometer Bruker maXis impact HD (USA) with the electrospray ionization probe installed coupled with Agilent 1260 HPLC system. Mass spectra were recorded with a Shimadzu GCMS-QP 2010 Ultra (Kyoto, Japan) in electron ionization (EI) mode (electron energy 70 eV). Elemental analyses were performed with a Perkin-Elmer 2400Series II CHNS/O analyzer (Shelton, CT USA). X-ray analyses were performed using an Xcalibur R Mo diffractometer (Agilent technologies, UK).

Synthesis of 3-dimethylaminothioamides 2a,e. General procedure. Corresponding thioamide (2 mmol) and DMF-DMA (477 mg, 4 mmol) were mixed at stirring which lead to self-heating, then the reaction mixture was let to cool to room temperature (crystallization was observed), and the stirring was continued for 0.5 h. Ethanol (2 mL) was added and the resulting suspension was stirred at room temperature for 0.5 h more. The precipitate thus formed was filtered off and washed with ethanol.

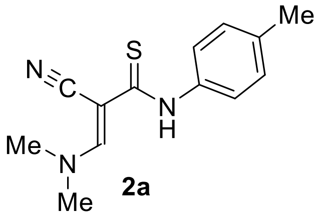

2a

*2-Cyano-3-(dimethylamino)-N-(p-tolyl)prop-2-enethioamide (**2a**)*. Compound **2a** was obtained in 90% yield (442 mg) according to the general procedure (DMF-DMA: 477 mg, 535 µL, 4.0 mmol; thioamide **1a**: 381 mg, 2.0 mmol) as a light yellow crystal powder, mp 208–209 °C. ^{1}H NMR (DMSO-d_6): *δ* 2.29 (s,

3H, CH_3), 3.26 (s, 3H, CH_3), 3.30 (s, 3H, CH_3), 7.14 (d, *J* 7.8 Hz, CH_{Ar}), 7.35–7.39 (m, 2H, CH_{Ar}), 8.30 (s, 1H, CH), 10.20 (s, 1H, NH). ^{13}C NMR (DMSO-d_6, the minor isomer signals are highlighted with an asterisk (*)): 20.6, 38.7, 47.4, 81.5, 118.8, 125.0/125.1*, 128.6, 134.6, 137.7/137.8*, 158.4*/158.5, 191.4*/191.5. EI-MS *m/z* (%): 247 $[M+2]^+$ (6), 246 $[M+1]^+$ (19), 245 $[M]^+$ (100), 244 (75), 213 (12), 212 (71), 201 (7), 141 (5), 140 (9), 139 (97), 124 (19), 123 (11), 122 (21), 121 (6), 118 (5), 112 (6), 107 (10), 106 (16), 98 (5), 96 (11), 95 (68), 91 (25), 89 (5), 81 (7), 80 (5), 79 (7), 78 (5), 77 (8), 68 (9), 65 (13), 54 (6). Analysis calculated for $C_{13}H_{15}N_3S$ (Mw = 245.34): C 63.64, H 6.16, N 17.13, S 13.07; found C 63.31, H 6.54, N 17.39, S 13.38%.

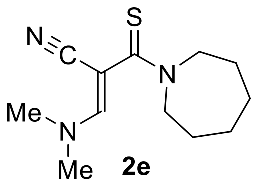

2e

2-(Azepane-1-carbonothioyl)-3-(dimethylamino)acrylonitrile (***2e***). Compound **2e** was obtained in 64% yield (304 mg) according to the general procedure (DMF-DMA: 477 mg, 535 µL, 4.0 mmol; 3-(azepan-1-yl)-3-thioxopropanenitrile: 365 mg, 2.0 mmol) as a light yellow crystal powder, mp 92–93 °C. ^{1}H NMR (DMSO-d_6): δ 1.41–1.57 (m, 4H, CH_2), 1.73–1.86 (m, 4H, CH_2), 3.18 (s, 6H, CH_3), 3.79–3.96 (m, 4H, NCH_2), 7.76 (s, 1H, CH). ^{13}C NMR (DMSO-d_6): 26.57, 27.11, 54.01, 79.69, 119.12, 158.47, 194.31. EI-MS m/z (%): 239 $[M+2]^+$ (5), 238 $[M+1]^+$ (12), 237 $[M]^+$ (73), 222 (29), 221 (8), 208 (7), 204 (14), 195 (8), 194 (25), 193 (100), 192 (8), 166 (12), 161 (5), 160 (29), 159 (16), 140 (6), 139 (30), 137 (9), 125 (7), 124 (5), 123 (7), 122 (30), 112 (5), 107 (16), 106 (7), 98 (21), 97 (6), 96 (10), 95 (31), 93 (5), 84 (5), 83 (5), 82 (5), 81 (11), 80 (7), 79 (5), 72 (9), 71 (5), 70 (5), 69 (9), 68 (10), 67 (5), 59 (7), 58 (6), 55 (25), 54 (7), 53 (5), 45 (11), 44 (15), 43 (11), 42 (47), 41 (35), 39 (11). Analysis calculated for $C_{12}H_{19}N_3S$ (Mw = 237.37): C 60.72, H 8.07, N 17.70, S 13.51; found C 60.94, H 8.31, N 17.92, S 13.54%.

Synthesis of 3(5)-aminopyrazoles 4a−c, 5a−e. General procedure. Hydrazine hydrate (80%) (**3a**) (1.1 mmol) was added to a solution of corresponding thioamide **1a**–**c** or **2a**–**e** (1.0 mmol) in ethanol (1–3 mL) and the resulting mixture was stirred at 80 °C (oil bath) during 4–6 h. The end of the reaction was determined by TLC (eluent DCM/EtOAc 12:1). The solvent was evaporated to dryness under reduced pressure, and the residue was purified by column chromatography over silica gel (eluent EtOAc, then EtOAc/EtOH, gradient from 6:1 to 3:1 (for compounds **4a**–**c**) or DCM, DCM/EtOAc (6:1), then EtOAc (for compounds **5a**–**e**)).

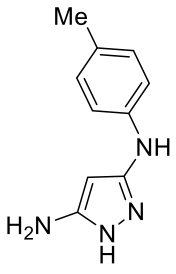

4a

*N^3-(p-Tolyl)-1H-pyrazole-3,5-diamine (**4a**).* Compound **4a** was obtained in 61% yield (115 mg) according to the general procedure (hydrazine **3a**: 69 mg, 67 µL, 1.1 mmol; thioamide **1a**: 190 mg, 1.0 mmol; EtOH (1 mL), 4 h) as a light gray crystal powder, mp 161–162 °C (lit. [12] 166–167 °C). ^{1}H NMR (DMSO-*d*$_6$): *δ* 2.17 (s, 3H, CH_3), 4.81 (br. s, 2H, NH_2), 4.90 (s, 1H, CH), 6.93 (d, *J* 8.1 Hz, CH_{Ar}), 7.15 (d, *J* 7.8 Hz, CH_{Ar}), 7.86 (s, 1H, NHAr), 10.43 (br. s, 1H, NH). ^{13}C NMR (DMSO-*d*$_6$): *δ* 20.2, 77.4, 114.8, 125.8, 129,0, 141.8, 148.6, 150.6. HRMS (ESI-TOF) *m/z*: [M + H]$^+$ Calcd. for $C_{10}H_{13}N_4^+$, 189.1135; Found: 189.1137.

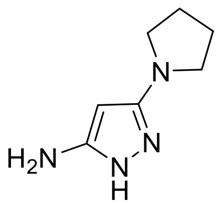

4b

*3-(Pyrrolidin-1-yl)-1H-pyrazol-5-amine (**4b**).* Compound **4b** was obtained in 77% yield (117 mg) according to the general procedure (hydrazine **3a**: 69 mg, 67 µL, 1.1 mmol; thioamide **1b**: 154 mg, 1.0 mmol; EtOH (1 mL), 6 h) as a brown oil. ^{1}H NMR (DMSO-*d*$_6$): *δ* 1.76–1.88 (m, 4H, CH_2), 3.01–3.10 (m, 4H, NCH_2), 4.58 (br. s, 1H, CH). ^{13}C NMR (DMSO-*d*$_6$): *δ* 24.7, 48.2, 74.5, 151.5, 154.2. HRMS (ESI-TOF) *m/z*: [M + H]$^+$ Calcd. for $C_7H_{13}N_4^+$, 153.1135; Found: 153.1137.

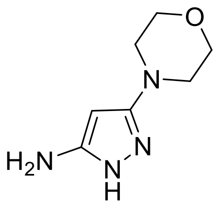

4c

*3-Morpholino-1H-pyrazol-5-amine (**4c**).* Compound **4c** was obtained in 76% yield (128 mg) according to the general procedure (hydrazine **3a**: 69 mg, 67 µL, 1.1 mmol; thioamide **1c**: 170 mg, 1.0 mmol; EtOH (1 mL), 4 h) as a light gray crystal powder mp 134–135 °C. ^{1}H NMR (DMSO-*d*$_6$): *δ* 2.90–2.98 (m, 4H, NCH_2), 3.60–3.67 (m, 4H, OCH_2), 4.78 (s, 1H, CH), 6.46 (br. s, 2H, NH_2). ^{1}H NMR ($CDCl_3$): *δ* 3.07–3.15 (m, 4H, NCH_2), 3.73–3.85 (m, 4H, OCH_2), 4.98 (s, 1H, CH), 5.64 (br. s, 3H, NH+NH_2). ^{13}C NMR (DMSO-*d*$_6$): *δ* 48.1, 65.8, 75.1, 150.2, 157.2. HRMS (ESI-TOF) *m/z*: [M + H]$^+$ Calcd. for $C_7H_{13}N_4O^+$, 169.1084; Found: 169.1084.

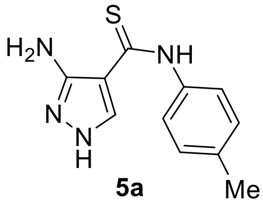

5a

*3-Amino-N-(p-tolyl)-1H-pyrazole-4-carbothioamide (**5a**).* Compound **5a** was obtained in 68% yield (158 mg) according to the general procedure (hydrazine **3a**: 69 mg, 67 µL, 1.1 mmol; thioamide **2a**: 245 mg, 1.0 mmol; EtOH (3 mL), 6 h) as a light yellow crystal powder, mp 250–251 °C. ^{1}H NMR (DMSO-*d*$_6$): *δ* 2.31 (s, 3H, CH_3), 6.98 (br. s, 2H, NH_2), 7.18 (d, *J* 7.9 Hz, CH_{Ar}), 7.37 (d, *J* 7.9 Hz, CH_{Ar}), 7.98 (s, 1H, CH), 10.49 (s, 1H, NH), 11.91 (br. s, 1H, NH). ^{13}C NMR (DMSO-*d*$_6$): *δ* 20.7, 103.8, 125.9, 128.8, 134.9, 136.8, 152.9, 186.7. HRMS (ESI-TOF) *m/z*: [M + H]$^+$ Calcd. for $C_{11}H_{13}N_4S^+$, 233.0855; Found: 233.0856.

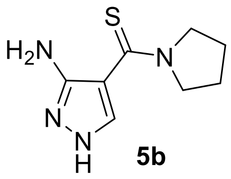 **5b**

(3-Amino-1H-pyrazol-4-yl)(pyrrolidin-1-yl)methanethione (**5b**). Compound **5b** was obtained in 75% yield (148 mg) according to the general procedure (hydrazine **3a**: 69 mg, 67 µL, 1.1 mmol; thioamide **2b**: 209 mg, 1.0 mmol; EtOH (1 mL), 4 h) as a colorless crystal powder, mp 236–237 °C. ^{1}H NMR (DMSO-*d*$_6$): *δ* 1.84–2.01 (m, 4H, CH_2), 3.69–3.88 (m, 4H, NCH_2), 6.63 (br. s, 2H, NH_2), 7.59 (br. s, 1H, CH), 11.88 (br. s, 1H, NH). ^{13}C NMR (DMSO-*d*$_6$): *δ* 23.8, 26.3, 53.3, 53.6, 103.6, 137.4, 152.3, 184.7. HRMS (ESI-TOF) *m/z*: $[M + H]^+$ Calcd. for $C_8H_{13}N_4S^+$, 197.0855; Found: 197.0856.

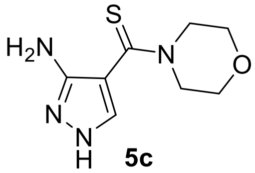 **5c**

(3-Amino-1H-pyrazol-4-yl)(morpholino)methanethione (**5c**). Compound **5c** was obtained in 78% yield (166 mg) according to the general procedure (hydrazine **3a**: 69 mg, 67 µL, 1.1 mmol; thioamide **2c**: 225 mg, 1.0 mmol; EtOH (1 mL), 5 h) as a colorless crystal powder, mp 187–188 °C. ^{1}H NMR (DMSO-*d*$_6$): *δ* 3.61–3.70 (m, 4H, NCH_2), 3.97–4.06 (m, 4H, OCH_2), 5.22 (br. s, 1H, N**H**H), 6.17 (br. s, 1H, NH**H**), 7.33 (br. s, 1H, CH), 11.85 (br. s, 1H, NH). ^{13}C NMR (DMSO-*d*$_6$): *δ* 50.9, 66.0, 104.9, 135.2, 151.3, 190.1. HRMS (ESI-TOF) *m/z*: $[M + H]^+$ Calcd. for $C_8H_{13}N_4OS^+$, 213.0805; Found: 213.0805.

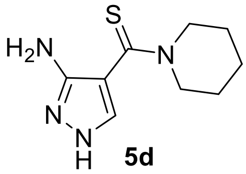 **5d**

(3-Amino-1H-pyrazol-4-yl)(piperidin-1-yl)methanethione (**5d**). Compound **5d** was obtained in 75% yield (157 mg) according to the general procedure (hydrazine **3a**: 69 mg, 67 µL, 1.1 mmol; thioamide **2d**: 223 mg, 1.0 mmol; EtOH (1 mL), 6 h) as a light yellow crystal powder, mp 194–195 °C. ^{1}H NMR (DMSO-*d*$_6$): *δ* 1.41–1.70 (m, 6H, CH_2), 3.87–4.07 (m, 4H, NCH_2), 5.94 (br. s, 2H, NH_2), 7.33 (br. s, 1H, CH), 11.91 (br. s, 1H, NH). ^{13}C NMR (DMSO-*d*$_6$): *δ* 23.9, 25.9, 51.4, 105.1, 134.6, 151.4, 189.1. HRMS (ESI-TOF) *m/z*: $[M + H]^+$ Calcd. for $C_9H_{15}N_4S^+$, 211.1012; Found: 211.1013.

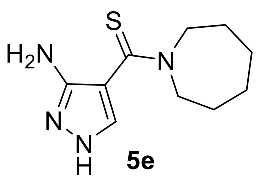 **5e**

(3-Amino-1H-pyrazol-4-yl)(azepan-1-yl)methanethione (**5e**). Compound **5e** was obtained in 72% yield (162 mg) according to the general procedure (hydrazine **3a**: 69 mg, 67 µL, 1.1 mmol; thioamide **2e**: 237 mg, 1.0 mmol; EtOH (1 mL), 6 h) as a colorless crystal powder, mp 137–138 °C. ^{1}H NMR (DMSO-*d*$_6$): *δ* 1.42–1.58 (m, 4H, CH_2), 1.67–1.89 (m, 4H, CH_2), 3.69–4.24 (m, 4H, NCH_2), 5.21 (br. s, 1H, N**H**H), 6.18 (br. s, 1H, NH**H**), 7.35 (br. s, 1H, CH), 11.83 (br. s, 1H, NH). ^{13}C NMR (DMSO-*d*$_6$): *δ* 26.6, 53.0, 105.3, 133.5, 151.8, 189.6. HRMS (ESI-TOF) *m/z*: $[M + H]^+$ Calcd. for $C_{10}H_{17}N_4S^+$, 225.1168; Found: 225.1169.

Synthesis of 5-aminopyrazoles 6a–d. General procedure. Corresponding aryl hydrazine **3b,c** (1.1 mmol) and 36% aq HCl (112 mg, 95 µL, 1.1 mmol) was added to a solution of corresponding thioamide **2a**–**d** (1.0 mmol) in EtOH (3 mL) and the resulting mixture was stirred at 80 °C (oil bath) during 12–16 h. The end of the reaction was determined by TLC (eluent DCM/EtOAc 12:1). The solvent was evaporated to dryness under reduced pressure, and the residue was purified by column chromatography over silica gel (eluent DCM, then DCM/EtOAc (6:1), finally EtOAc) to afford corresponding 5-aminopyrazole **6a**–**d**. To obtain compounds **6e,f**, hydrazine hydrochloride **3c** was used. The precipitate formed as a result of the reaction, was filtered off and crystallized from ethanol.

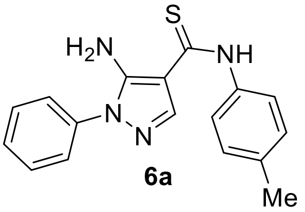

6a

*5-Amino-1-phenyl-N-(p-tolyl)-1H-pyrazole-4-carbothioamide (**6a**)*. Compound **6a** was obtained in 80% yield (247 mg) according to the general procedure (phenylhydrazine **3b**: 119 mg, 109 µL, 1.1 mmol); thioamide **1a**: 245 mg, 1.0 mmol; 16 h) as a light yellow crystal powder, mp 248–250 °C. ^{1}H NMR (DMSO-*d*$_6$): *δ* 2.33 (s, 3H, CH_3), 7.21 (br. s, 1H, N**H**H), 7.23 (br. s, 1H, NH**H**), 7.38–7.57 (m, 9H, CH_{Ar}), 8.25 (s, 1H, CH), 10.69 (s, 1H, NH). ^{13}C NMR (DMSO-*d*$_6$): 20.7, 103.7, 123.9, 126.0, 127.6, 128.8, 129.5, 135.2, 136.6, 136.7, 138.0, 150.5, 186.4. HRMS (ESI-TOF) *m/z*: [M + H]$^+$ Calcd. for $C_{17}H_{17}N_4S^+$, 309.1168; Found: 309.1166.

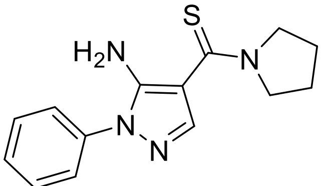

6b

*5-Amino-1-phenyl-1H-pyrazol-4-yl)(pyrrolidin-1-yl)methanethione (**6b**).* Compound **6b** was obtained in 70% yield (191 mg) according to the general procedure (phenylhydrazine **3b**: 119 mg, 109 µL, 1.1 mmol; thioamide **2b**: 209 mg, 1.0 mmol; 14 h) as a colorless crystal powder, mp 159–160 °C. ^{1}H NMR (DMSO-*d*$_6$): *δ* 1.84–2.07 (m, 4H, CH_2), 3.69–4.03 (m, 4H, NCH_2), 7.06 (br. s, 2H, NH_2), 7.38–7.46 (m, 1H, CH_{Ph}), 7.51–7.60 (m, 4H, CH_{Ph}), 7.81 (s, 1H, CH). ^{13}C NMR (DMSO-*d*$_6$): 23.7, 26.4, 53.4, 53.8, 103.8, 123.8, 127.4, 129.4, 138.1, 138.2, 149.7, 184.2. HRMS (ESI-TOF) *m/z*: [M + H]$^+$ Calcd. for $C_{14}H_{17}N_4S^+$, 273.1168; Found: 273.1170.

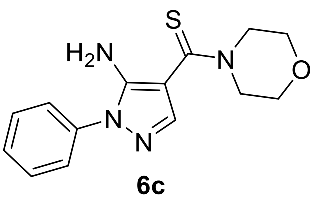

6c

*(5-Amino-1-phenyl-1H-pyrazol-4-yl)(morpholino)methanethione (**6c**).* Compound **6c** was obtained in 63% yield (182 mg) according to the general procedure (phenylhydrazine **3b**: 119 mg, 109 µL, 1.1 mmol; thioamide **2c**: 225 mg, 1.0 mmol; 12 h) as a colorless crystal powder, mp 160–161 °C. ^{1}H NMR (DMSO-*d*$_6$): *δ* 3.66–3.74 (m, 4H, NCH_2), 4.03–4.13 (m, 4H, OCH_2), 6.43 (br. s, 1H, N**H**H), 6.45 (br. s, 1H, NH**H**), 7.40–7.43

(m, 1H, CH_{Ph}), 7.52–7.57 (m, 5H, CH_{Ph} + CH). ^{13}C NMR (DMSO-d_6): 51.1, 66.1, 104.4, 123.8, 127.5, 129.5, 138.1, 139.0, 147.9, 189.3. HRMS (ESI-TOF) *m/z*: $[M + H]^+$ Calcd. for $C_{14}H_{17}N_4OS^+$, 289.1118; Found: 289.1118.

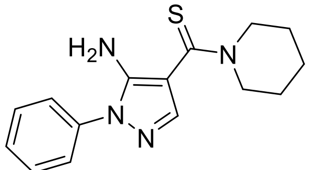

6d

*(5-Amino-1-phenyl-1H-pyrazol-4-yl)(piperidin-1-yl)methanethione (**6d**).* Compound **6d** was obtained in 68% yield (195 mg) according to the general procedure (phenylhydrazine **3b**: 119 mg, 109 µL, 1.1 mmol; thioamide **2d**: 223 mg, 1.0 mmol; 16 h) as a light yellow crystal powder, mp 171–173 °C. ^{1}H NMR (DMSO-d_6): δ 1.57–1.74 (m, 6H, CH_2), 3.97–4.08 (m, 4H, NCH_2), 6.38 (br. s, 2H, NH_2), 7.39–7.42 (m, 1H, CH_{Ph}), 7.51–7.68 (m, 5H, CH_{Ph} + CH). ^{13}C NMR (DMSO-d_6): 23.8, 25.9, 51.5, 104.7, 123.6, 127.3, 129.4, 138.2, 138.6, 147.7, 188.3. HRMS (ESI-TOF) *m/z*: $[M + H]^+$ Calcd. for $C_{15}H_{19}N_4S^+$, 287.1325; Found: 287.1325.

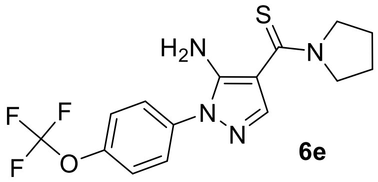

6e

*(5-Amino-1-(4-(trifluoromethoxy)phenyl)-1H-pyrazol-4-yl)(pyrrolidin-1-yl)methanethione (**6e**).* Compound **6e** was obtained in 86% yield (306 mg) according to the general procedure (arylhydrazine hydrochloride **3c**: 252 mg, 1.1 mmol; thioamide **2b**: 209 mg, 1.0 mmol; 14 h) as a colorless crystal powder, mp 159–160 °C. ^{1}H NMR (DMSO-d_6): δ 1.90–1.99 (m, 4H, CH_2), 3.75–3.93 (m, 4H, NCH_2), 7.12 (br. s, 1H, N**H**H), 7.14 (br. s, 1H, NH**H**), 7.54 (d, *J* 8.4 Hz, CH_{Ar}), 7.70 (d, *J* 8.8 Hz, CH_{Ar}), 7.84 (s, 1H, CH). ^{13}C NMR (DMSO-d_6): 30.7, 53.6, 104.1, 120.1 (q, *J* 256.7 Hz), 122.1, 125.6, 137.1, 138.6, 146.9, 149.8, 184.1. ^{19}F NMR (DMSO-d_6): -56.86. HRMS (ESI-TOF) *m/z*: $[M + H]^+$ Calcd. for $C_{15}H_{16}F_3N_4OS^+$, 357.0991; Found: 357.0992.

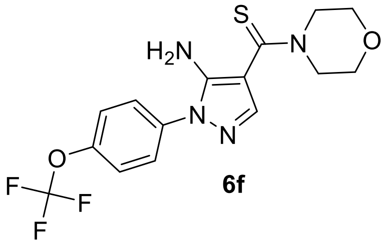

6f

*(5-Amino-1-(4-(trifluoromethoxy)phenyl)-1H-pyrazol-4-yl)(morpholino)methanethione (**6f**).* Compound **6f** was obtained in 71% yield (264 mg) according to the general procedure (arylhydrazine hydrochloride **3c**: 252 mg, 1.1 mmol; thioamide **2c**: 225 mg, 1.0 mmol; 16 h) as a colorless crystal powder, mp 144–145 °C. ^{1}H NMR (DMSO-d_6): δ 3.62–3.74 (m, 4H, NCH_2), 4.01–4.12 (m, 4H, OCH_2), 6.49 (br. s, 1H, N**H**H), 6.52 (br. s, 1H, NH**H**), 7.54 (d, *J* 8.5 Hz, CH_{Ar}), 7.59 (s, 1H, CH), 7.70 (d, *J* 8.6 Hz, CH_{Ar}). ^{13}C NMR (DMSO-d_6): 51.0, 66.0, 104.7 (d, *J* 5.1 Hz), 120.1 (q, *J* 256.6 Hz), 122.0, 125.6 (d, *J* 2.1 Hz), 137.1, 139.6 (d, *J* 2.5 Hz), 146.9, 147.7 (d, *J* 10.9 Hz), 189.2. ^{19}F NMR (DMSO-d_6): -56.86. HRMS (ESI-TOF) *m/z*: $[M + H]^+$ Calcd. for $C_{15}H_{16}F_3N_4O_2S^+$, 373.0940; Found: 373.0940.

Synthesis of 5-aminopyrazoles 7a–j. General procedure. Corresponding arylsulfonylhydrazine **3d**–**g** (1.1 mmol) and 36% aq. HCl (112 mg, 95 µL, 1.1 mmol) was added to a solution of corresponding thioamide **2b,d,e** (1.0 mmol) in EtOH (6 mL) and the resulting mixture was stirred at room temperature during 12–16 h. The end of the reaction was determined by TLC (eluent DCM/EtOAc 12:1). The formed precipitate was filtered off, washed with EtOH and crystallized from EtOH to afford corresponding 5-aminopyrazole **7a**–**j**.

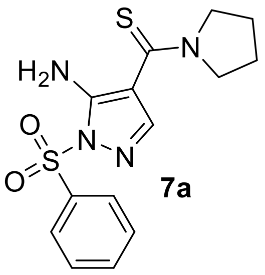
7a

*(5-Amino-1-(phenylsulfonyl)-1H-pyrazol-4-yl)(pyrrolidin-1-yl)methanethione (**7a**)*. Compound **7a** was obtained in 92% yield (310 mg) according to the general procedure (sulfonylhydrazine **3d**: 189 mg, 1.1 mmol; thioamide **2b**: 209 mg, 1.0 mmol; 12 h) as a colorless crystal powder, mp 159–160 °C. ^{1}H NMR (DMSO-d_6): δ 1.82–1.93 (m, 4H, CH_2), 3.63–3.76 (m, 4H, NCH_2), 7.69 (t, *J* 7.5 Hz, CH_{Ph}), 7.76 (br. s, 2H, NH_2), 7.79–7.82 (m, 1H, CH_{Ph}), 7.90 (s, 1H, CH), 7.98 (d, *J* 7.5 Hz, CH_{Ph}). ^{13}C NMR (DMSO-d_6): δ 23.6, 26.2, 53.5, 102.4, 127.4, 129.9, 135.2, 136.4, 143.3, 151.9, 182.8. HRMS (ESI-TOF) *m/z*: [M + H]$^+$ Calcd. for $C_{14}H_{17}N_4O_2S_2^+$, 337.0787; Found: 337.0789.

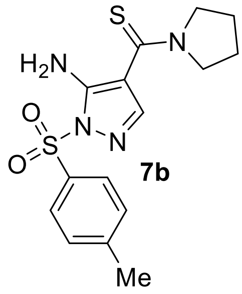
7b

*(5-Amino-1-tosyl-1H-pyrazol-4-yl)(pyrrolidin-1-yl)methanethione (**7b**)*. Compound **7b** was obtained in 89% yield (312 mg) according to the general procedure (tosylhydrazine **3e**: 205 mg, 1.1 mmol; thioamide **2b**: 209 mg, 1.0 mmol; 16 h) as a colorless crystal powder, mp 148–149 °C. ^{1}H NMR (DMSO-d_6): δ 1.84–1.93 (m, 4H, CH_2), 2.39 (s, 3H, CH_3), 3.66–3.74 (m, 4H, NCH_2), 7.48 (d, *J* 7.9 Hz, CH_{Ar}), 7.74 (br. s, 2H, NH_2), 7.85–7.87 (m, 3H, CH_{Ar} + CH). ^{13}C NMR (DMSO-d_6): δ 21.2, 23.6, 26.2, 53.5, 102.4, 127.5, 130.3, 133.5, 143.1, 146.4, 151.8, 182.9. HRMS (ESI-TOF) *m/z*: [M + H]$^+$ Calcd. for $C_{15}H_{19}N_4O_2S_2^+$, 351.0944; Found: 351.0950.

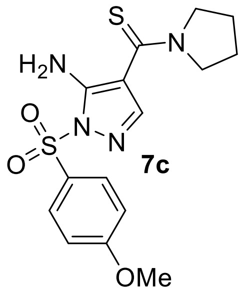
7c

*(5-Amino-1-((4-methoxyphenyl)sulfonyl)-1H-pyrazol-4-yl)(pyrrolidin-1-yl)methanethione (**7c**)*. Compound **7c** was obtained in 94% yield (346 mg) according to the general procedure (sulfonylhydrazine **3f**: 222 mg, 1.1 mmol; thioamide **2b**: 209 mg, 1.0 mmol; 16 h) as a colorless crystal powder, mp 151–152 °C. ^{1}H NMR (DMSO-d_6): δ 1.82–1.94 (m, 4H, CH_2), 3.66–3.76 (m, 4H, NCH_2), 3.85 (s, 3H, OCH_3), 7.19 (d, *J* 8.9 Hz, CH_{Ar}), 7.72 (s, 2H, NH_2), 7.87 (s, 1H, CH), 7.92 (d, *J* 8.8 Hz, CH_{Ar}). ^{13}C NMR (DMSO-d_6): δ 23.6, 26.2, 53.5, 53.5, 56.0, 102.3, 115.1, 127.5, 130.0,

142.8, 151.7, 164.3, 182.9. HRMS (ESI-TOF) *m/z*: $[M + H]^+$ Calcd. for $C_{15}H_{19}N_4O_3S_2^+$, 367.0893; Found: 367.0892.

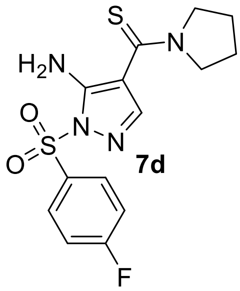
7d

*(5-Amino-1-((4-fluorophenyl)sulfonyl)-1H-pyrazol-4-yl)(pyrrolidin-1-yl)methanethione (**7d**)*. Compound **7d** was obtained in 91% yield (323 mg) according to the general procedure (sulfonylhydrazine **3g**: 209 mg, 1.1 mmol; thioamide **2b**: 209 mg, 1.0 mmol; 16 h) as a colorless crystal powder, mp 191–192 °C. ^{1}H NMR (DMSO-d_6): δ 1.83–1.94 (m, 4H, CH_2), 3.66–3.77 (m, 4H, NCH_2), 7.53–7.57 (m, 2H, CH_{Ar}), 7.76 (s, 2H, NH_2), 7.91 (s, 1H, CH), 8.05–8.08 (m, 2H, CH_{Ar}). ^{13}C NMR (DMSO-d_6): δ 23.6, 26.2, 53.5, 102.4, 117.3 (d, *J* 23.2 Hz), 130.7 (d, *J* 10.3 Hz), 132.6 (d, *J* 2.7 Hz), 143.5, 151.8, 165.7 (d, *J* 255.4 Hz), 182.8. ^{19}F NMR (DMSO-d_6): -101.82. HRMS (ESI-TOF) *m/z*: $[M + H]^+$ Calcd. for $C_{14}H_{16}FN_4O_2S_2^+$, 355.0693; Found: 355.0694.

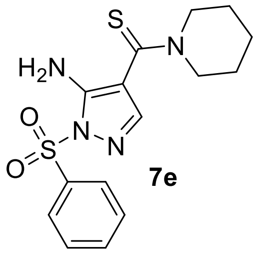
7e

*(5-Amino-1-(phenylsulfonyl)-1H-pyrazol-4-yl)(piperidin-1-yl)methanethione (**7e**)*. Compound **7e** was obtained in 84% yield (294 mg) according to the general procedure (sulfonylhydrazine **3d**: 189 mg, 1.1 mmol; thioamide **2d**: 223 mg, 1.0 mmol; 14 h) as a colorless crystal powder, mp 145–146 °C. ^{1}H NMR (DMSO-d_6): δ 1.44–1.68 (m, 6H, CH_2), 3.69–3.98 (m, 4H, NCH_2), 6.99 (s, 2H, NH_2), 7.60 (s, 1H, CH), 7.69 (t, *J* 7.6 Hz, CH_{Ph}), 7.80 (t, *J* 7.0 Hz, CH_{Ph}), 7.98 (d, *J* 7.8 Hz, CH_{Ph}). ^{13}C NMR (DMSO-d_6): δ 23.62, 25.73, 51.41, 103.6/103.7*, 127.40, 129.86, 135.18, 136.42, 144.6*/144.7, 148.8*/148.9, 186.54. EI-MS m/z (%): 352 $[M+2]^+$ (9), 351 $[M+1]^+$ (17), 350 $[M]^+$ (82), 266 (12), 249 (9), 209 (10), 181 (7), 164 (5), 154 (29), 141 (14), 128 (5), 127 (7), 126 (84), 125 (9), 121 (12), 120 (7), 98 (5), 86 (5), 85 (7), 84 (100), 78 (6), 77 (54), 71 (7), 69 (21), 55 (6), 54 (8), 53 (5), 52 (6), 51 (12), 42 (8), 41 (31), 39 (6). Analysis calculated for $C_{15}H_{18}N_4O_2S_2$ (Mw = 350.45): C 51.41, H 5.18, N 15.99, S 18.30; found C 51.51, H 5.09, N 16.09, S 17.92%.

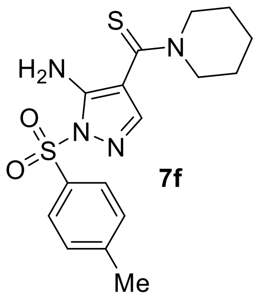
7f

*(5-Amino-1-tosyl-1H-pyrazol-4-yl)(piperidin-1-yl)methanethionemethanethione (**7f**)*. Compound **7f** was obtained in 90% yield (328 mg) according to the general procedure (tosylhydrazine **3e**: 205 mg, 1.1 mmol; thioamide **2d**: 223 mg, 1.0 mmol; 14 h) as a colorless crystal powder, mp 125–126 °C. ^{1}H NMR (DMSO-d_6): δ 1.44–1.67 (m, 6H, CH_2), 2.39 (s, 3H, CH_3), 3.74–3.94 (m, 4H, NCH_2), 6.97 (s, 2H, NH_2), 7.48 (d, *J* 7.9 Hz, CH_{Ar}), 7.58 (s, 1H, CH), 7.86 (d, *J* 8.0 Hz, CH_{Ar}). ^{13}C NMR (DMSO-d_6): δ 21.1, 23.6, 25.7, 51.4, 103.6, 127.5, 130.3, 133.5,

144.4, 146.2, 148.8, 186.6. HRMS (ESI-TOF) *m/z*: $[M + H]^+$ Calcd. for $C_{16}H_{21}N_4O_2S_2^+$, 365.1100; Found: 365.1100.

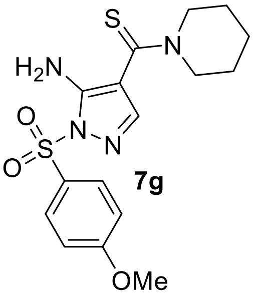

7g

*(5-Amino-1-((4-methoxyphenyl)sulfonyl)-1H-pyrazol-4-yl)(piperidin-1-yl)methanethione (**7g**)*. Compound **7g** was obtained in 84% yield (320 mg) according to the general procedure (sulfonylhydrazine **3f**: 222 mg, 1.1 mmol; thioamide **2d**: 223 mg, 1.0 mmol; 16 h) as a light yellow crystal powder, mp 156–157 °C. ^{1}H NMR (DMSO-*d*$_6$): *δ* 1.48–1.68 (m, 6H, CH_2), 3.75–3.93 (m, 7H, NCH_2 + OCH_3), 6.96 (br. s, 2H, NH_2), 7.19 (d, *J* 8.9 Hz, CH_{Ar}), 7.57 (br. s, 1H, CH), 7.92 (d, *J* 8.8 Hz, CH_{Ar}). ^{13}C NMR (DMSO-*d*$_6$): *δ* 23.6, 25.7, 51.4, 56.0, 103.5, 115.1, 127.6, 130.0, 144.1, 148.7, 164.3, 186.7. EI-MS m/z (%): 382 $[M+2]^+$ (9), 381 $[M+1]^+$ (17), 380 $[M]^+$ (80), 296 (8), 279 (6), 209 (14), 181 (9), 171 (22), 155 (6), 154 (33), 128 (5), 127 (6), 126 (72), 125 (5), 123 (11), 121 (12), 120 (7), 107 (25), 98 (5), 92 (18), 86 (5), 85 (7), 84 (100), 77 (26), 72 (6), 71 (8), 70 (5), 69 (23), 64 (8), 63 (5), 56 (8), 55 (8), 54 (7), 42 (8), 41 (31), 39 (6). Analysis calculated for $C_{16}H_{20}N_4O_3S_2$ (Mw = 380.48): C 50.51, H 5.30, N 14.73, S 16.85; found C 50.68, H 5.09, N 15.10, S 16.55%.

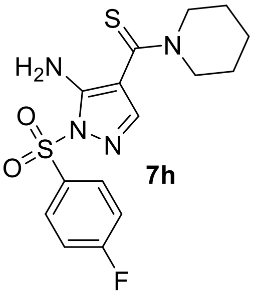

7h

*(5-Amino-1-((4-fluorophenyl)sulfonyl)-1H-pyrazol-4-yl)(piperidin-1-yl)methanethione (**7h**)*. Compound **7h** was obtained in 76% yield (280 mg) according to the general procedure (sulfonylhydrazine **3g**: 209 mg, 1.1 mmol; thioamide **2d**: 223 mg, 1.0 mmol; 16 h) as a light yellow crystal powder, mp 144–145 °C. ^{1}H NMR (DMSO-*d*$_6$): *δ* 1.49–1.67 (m, 6H, CH_2), 3.74–3.94 (m, 4H, NCH_2), 6.99 (s, 2H, NH_2), 7.55 (t, *J* 8.7 Hz, CH_{Ar}), 7.61 (s, 1H, CH), 8.06–8.08 (m, 2H, CH_{Ar}). ^{13}C NMR (DMSO-*d*$_6$): *δ* 23.62, 25.72, 51.44, 103.71 (d, *J* 2.4 Hz), 117.28 (d, *J* 23.2 Hz), 130.88 (d, *J* 10.2 Hz), 132.65 (d, *J* 2.4 Hz), 144.91, 148.69 (d, *J* 12.2 Hz), 165.68 (d, *J* 255.4 Hz), 186.48. EI-MS m/z (%): 370 $[M+2]^+$ (9), 369 $[M+1]^+$ (16), 368 $[M]^+$ (74), 325 (5), 284 (12), 267 (9), 209 (11), 181 (8), 164 (5), 159 (13), 154 (30), 128 (6), 127 (7), 126 (87), 125 (10), 121 (13), 120 (7), 98 (5), 96 (7), 95 (38), 86 (5), 85 (7), 84 (100), 75 (9), 72 (5), 71 (7), 69 (23), 56 (9), 55 (7), 54 (8), 53 (5), 52 (5), 42 (7), 41 (27). Analysis calculated for $C_{15}H_{17}FN_4O_2S_2$ (Mw = 368.45): C 48.90, H 4.65, N 15.21, S 17.40; found C 48.90, H 4.67, N 14.91, S 17.22%.

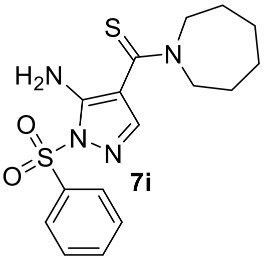
7i

*(5-Amino-1-(phenylsulfonyl)-1H-pyrazol-4-yl)(azepan-1-yl)methanethione (**7i**)*. Compound **7i** was obtained in 91% yield (332 mg) according to the general procedure (sulfonylhydrazine **3d**: 189 mg, 1.1 mmol; thioamide **2e**: 237 mg, 1.0 mmol; 12 h) as a colorless crystal powder, mp 149–150 °C. ^{1}H NMR (DMSO-d_6): δ 1.33–1.88 (m, 8H, CH_2), 3.57–4.04 (m, 4H, NCH_2), 7.02 (s, 2H, NH_2), 7.62 (s, 1H, CH), 7.68 (t, J 7.8 Hz, CH_{Ph}), 7.80 (t, J 7.5 Hz, CH_{Ph}), 7.97 (t, J 7.4 Hz, CH_{Ph}). ^{13}C NMR (DMSO-d_6): δ 25.70, 27.05, 28.06, 52.25, 53.78, 104.00, 127.36, 129.84, 135.14, 136.43, 144.11, 149.2, 187.38. EI-MS m/z (%): 366 [M + 2]$^+$ (11), 365 [M+1]$^+$ (20), 364 [M]$^+$ (94), 332 (5), 331 (23), 307 (5), 282 (5), 266 (27), 263 (9), 249 (16), 223 (23), 222 (5), 195 (21), 178 (8), 168 (40), 166 (5), 141 (28), 140 (6), 135 (13), 134 (12), 127 (5), 126 (61), 125 (14), 99 (8), 98 (100), 97 (8), 96 (9), 86 (5), 84 (5), 83 (6), 81 (5), 79 (5), 78 (8), 77 (73), 72 (5), 71 (8), 70 (7), 69 (19), 67 (5), 56 (8), 55 (46), 54 (11), 53 (8), 52 (8), 51 (14), 44 (7), 43 (7), 42 (8), 41 (35), 39 (7). Analysis calculated for $C_{16}H_{20}N_4O_2S_2$ (Mw = 364.48): C 52.73, H 5.53, N 15.37, S 17.59; found C 52.44, H 5.64, N 15.65, S 17.92%.

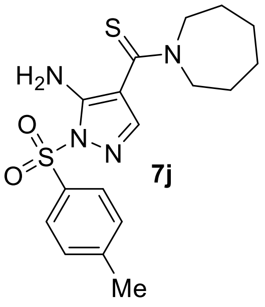
7j

*(5-Amino-1-tosyl-1H-pyrazol-4-yl)(azepan-1-yl)methanethione (**7j**)*. Compound **7j** was obtained in 90% yield (341 mg) according to the general procedure (tosylhydrazine **3e**: 205 mg, 1.1 mmol; thioamide **2e**: 237 mg, 1.0 mmol; 16 h) as a colorless crystal powder, mp 131–132 °C. ^{1}H NMR (DMSO-d_6): δ 1.32–1.88 (m, 8H, CH_2), 2.39 (s, 3H, CH_3), 3.57–4.05 (m, 4H, NCH_2), 7.00 (s, 2H, NH_2), 7.48 (d, J 7.1 Hz, CH_{Ar}), 7.60 (s, 1H, CH), 7.85 (d, J 7.1 Hz, CH_{Ar}). ^{13}C NMR (DMSO-d_6): δ 21.13, 25.62, 27.06, 28.03, 52.25, 53.77, 103.9, 127.43, 130.25, 133.54, 143.83, 146.13, 149.2, 187.42. EI-MS m/z (%): 380 [M+2]$^+$ (12), 379 [M+1]$^+$ (23), 378 [M]$^+$ (100), 346 (5), 345 (22), 321 (5), 280 (25), 277 (8), 263 (14), 223 (23), 222 (5), 195 (19), 178 (7), 168 (35), 155 (23), 141 (6), 140 (5), 135 (11), 134 (10), 127 (5), 126 (56), 125 (8), 99 (8), 98 (98), 97 (6), 96 (8), 92 (9), 91 (83), 86 (5), 83 (5), 81 (5), 79 (5), 71 (7), 70 (6), 69 (17), 65 (20), 56 (7), 55 (42), 54 (9), 53 (7), 52 (6), 44 (7), 43 (7), 42 (9), 41 (39), 39 (12). Analysis calculated for $C_{17}H_{22}N_4O_2S_2$ (Mw = 378.51): C 53.95, H 5.86, N 14.80, S 16.94; found C 53.66, H 5.86, N 15.19, S 17.15%.

*(5-Amino-4-(pyrrolidine-1-carbonothioyl)-1H-pyrazol-1-yl)(p-tolyl)methanone (**7k**)*. Benzoylhydrazine **3h** (165 mg, 1.1 mmol) and 36% aq. HCl (112 mg, 95 µL, 1.1 mmol) were added to a solution of thioamide **2b** (209 mg, 1.0 mmol) in ethanol (6 mL) and the resulting mixture was stirred at 60 ºC (oil bath) during 5 h. The formed precipitate was

filtered off, washed with ethanol and crystallized from ethanol to afford corresponding 5-

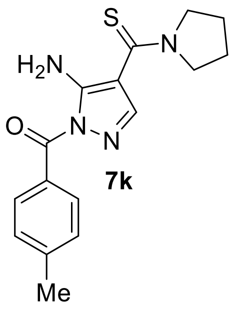

aminopyrazole **7k** as a colorless crystal powder in 72% yield (226 mg), mp 179–180 °C. ^{1}H NMR (DMSO-d_6): δ 1.88–1.99 (m, 4H, CH_2), 2.40 (s, 3H, CH_3), 3.72–3.89 (m, 4H, NCH_2), 7.34 (d, *J* 7.8 Hz, CH_{Ar}), 7.88–7.91 (m, 3H, CH_{Ar}+CH), 8.23 (s, 2H, NH_2). ^{13}C NMR (DMSO-d_6): δ 21.17, 23.68, 26.30, 53.40, 53.55, 102.19, 128.43, 129.71, 130.89, 141.67, 143.09, 153.24, 169.55, 183.20. HRMS (ESI-TOF) *m/z*: $[M + H]^+$ Calcd. for $C_{16}H_{19}N_4OS^+$, 315.1274; Found: 315.1276.

***Synthesis of 1-NH-3-aminopyrazole 5b from* 7b**. 5-Aminopyrazole **7b** (350 mg, 1 mmol) was dissolved in ethanol (6 mL). Then 36% aq. HCl (102 mg, 87 µL, 1 mmol) was added and the resulting solution was heated with stirring to 80 ºC for 24 h. The solvent was evaporated to dryness under reduced pressure, and the residue was purified by column chromatography over silica gel (eluent DCM, then DCM/MeOH (12:1)) to afford compound **5b** in 97% yield (190 mg) as a colorless crystal powder, mp 236–237 °C.

References

1. Dankova, E. F.; Bakulev, V. A.; Kolobov, M. Y.; Shishkina, V. I.; Yasman, Ya. B.; Lebedev, A. T. *Chem. Heterocycl. Compds.* **1988**, *24*, 1051–1055. DOI: 10.1007/BF00474054.

2. Kosterina, M. F.; Morzherin, Y. Y.; Tkachev, A. V.; Rybalova, T. V.; Gatilov, Yu. V.; Bakulev, V. A. *Russ. Chem. Bull.* **2002**, *51*, 653–658. DOI: 10.1023/A:1015868201847.

3. Lugovik, K. I.; Eltyshev, A. K.; Benassi, E.; Belskaya, N. P. *Chem. Asian J.* **2017**, *12*, 2410–2425. DOI: 10.1002/asia.201700721.
