# Supplementary material for: Cyanothioacetamides as a synthetic platform for the synthesis of aminopyrazole derivatives
Source: Beilstein J Org Chem. 2023 Aug 8;19:1191–7. doi: 10.3762/bjoc.19.87 (PMC10428587; doi:10.3762/bjoc.19.87)

BEILSTEIN JOURNAL OF ORGANIC CHEMISTRY

Supporting Information

for

Cyanothioacetamides as a synthetic platform for the synthesis of aminopyrazole derivatives

Valeriy O. Filimonov, Alexandra I. Topchiy, Vladimir G. Ilkin, Tetyana V. Beryozkina and Vasiliy A. Bakulev

Beilstein J. Org. Chem. **2023,** *19,* 1191–1197. doi:10.3762/bjoc.19.87

Copies of NMR spectra of all new compounds

License and Terms: This is a supporting information file under the terms of the Creative Commons Attribution License (https://creativecommons.org/licenses/by/4.0). Please note that the reuse, redistribution and reproduction in particular requires that the author(s) and source are credited and that individual graphics may be subject to special legal provisions.
The license is subject to the *Beilstein Journal of Organic Chemistry* terms and conditions: (https://www.beilstein-journals.org/bjoc/terms)

NMR spectra of compounds 2a,e, 4a–c, 5a–e, 6a–f, 7a–k

Figure S1. ^{1}H NMR spectrum (DMSO-d_6) of **2a**.

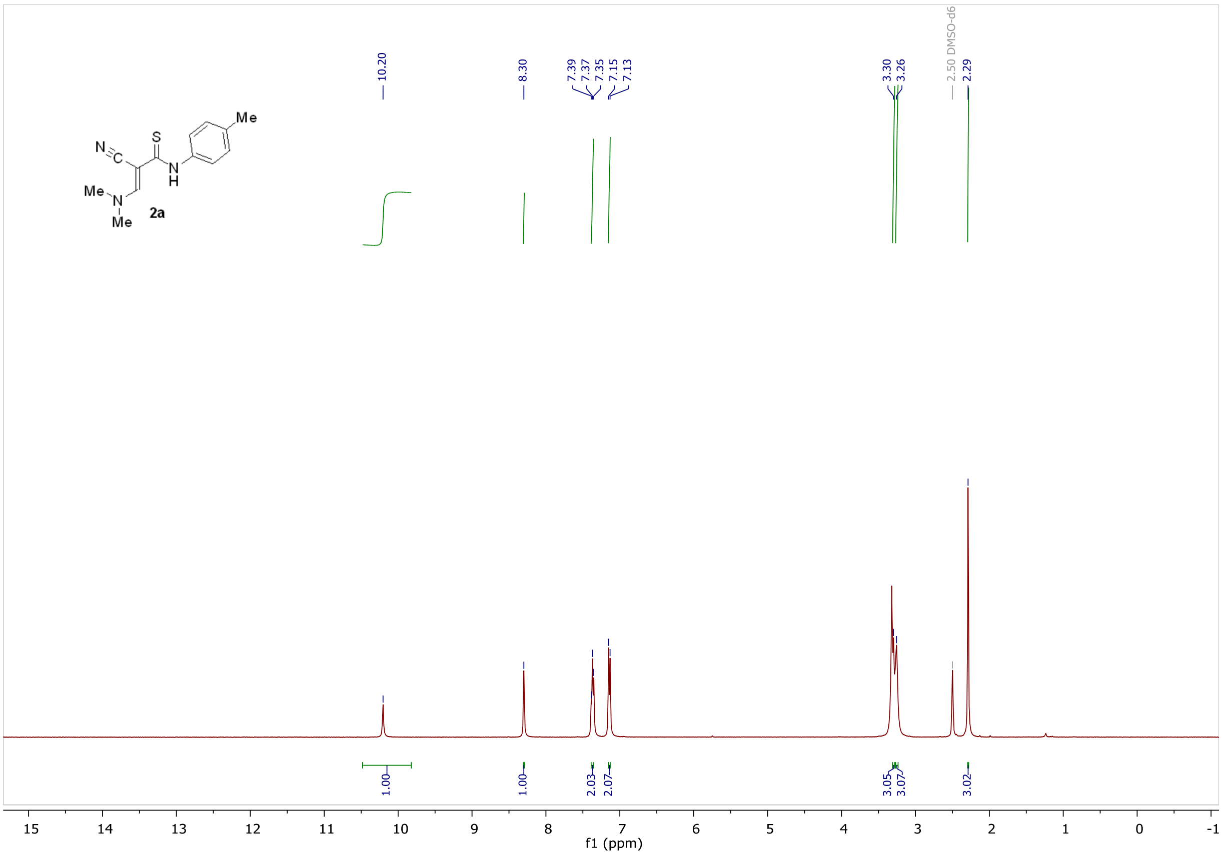

Figure S2. ^{13}C NMR spectrum (DMSO-d_6) of **2a**.

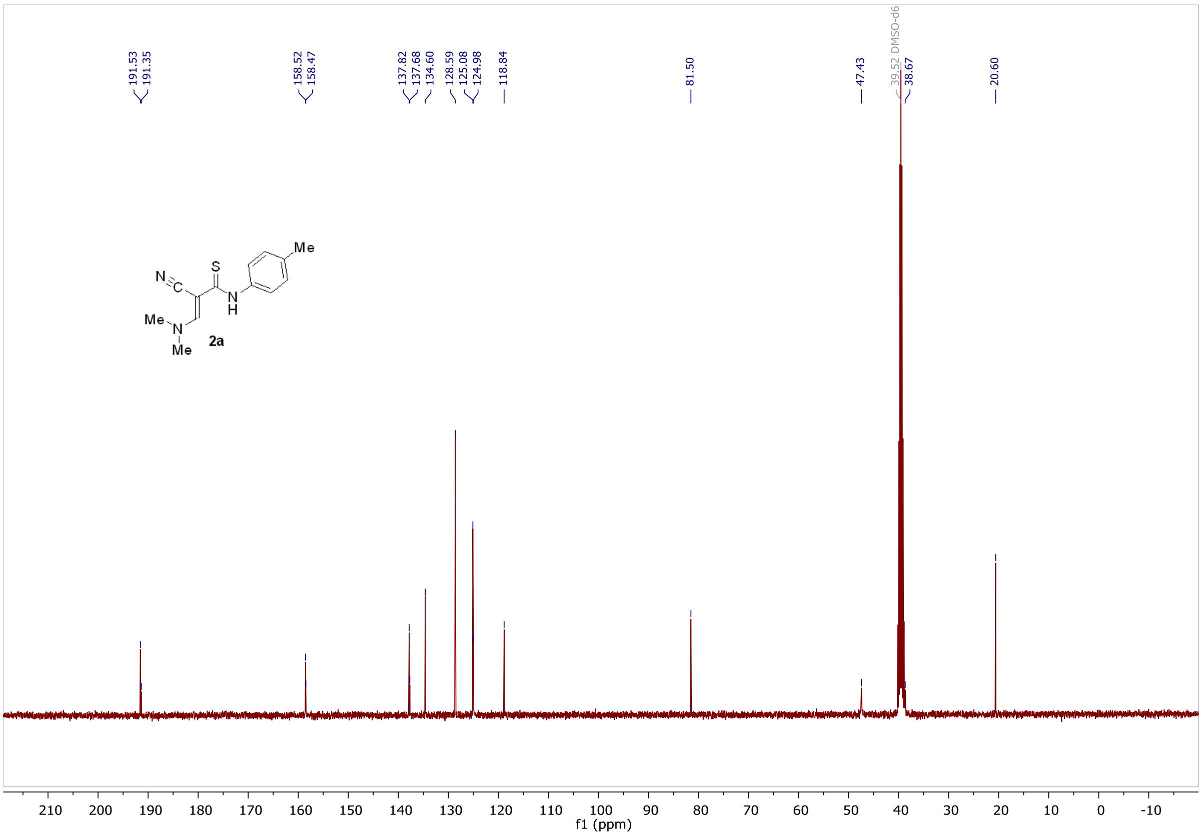

Figure S3. ^{1}H NMR spectrum (DMSO-d_6) of **2e**.

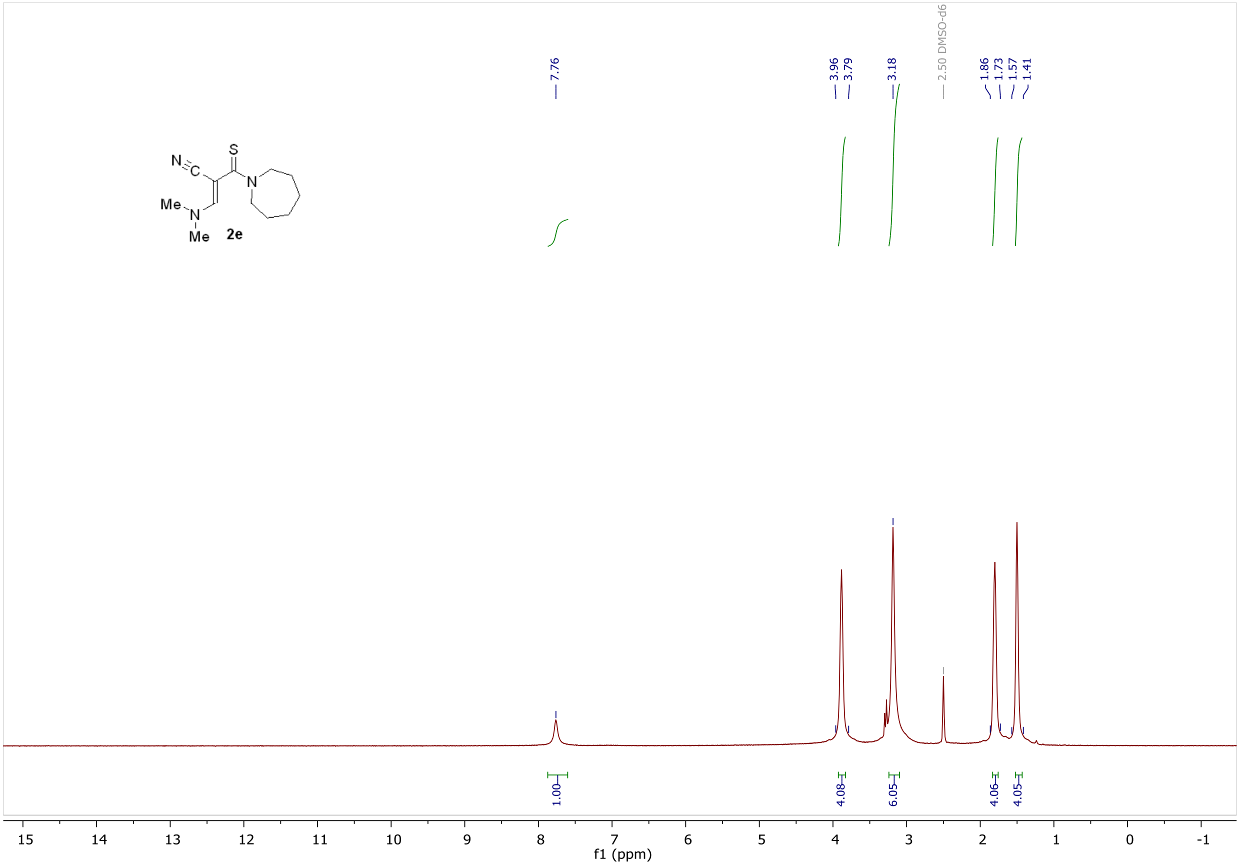

Figure S4. ^{13}C NMR spectrum (DMSO-d_6) of **2e**.

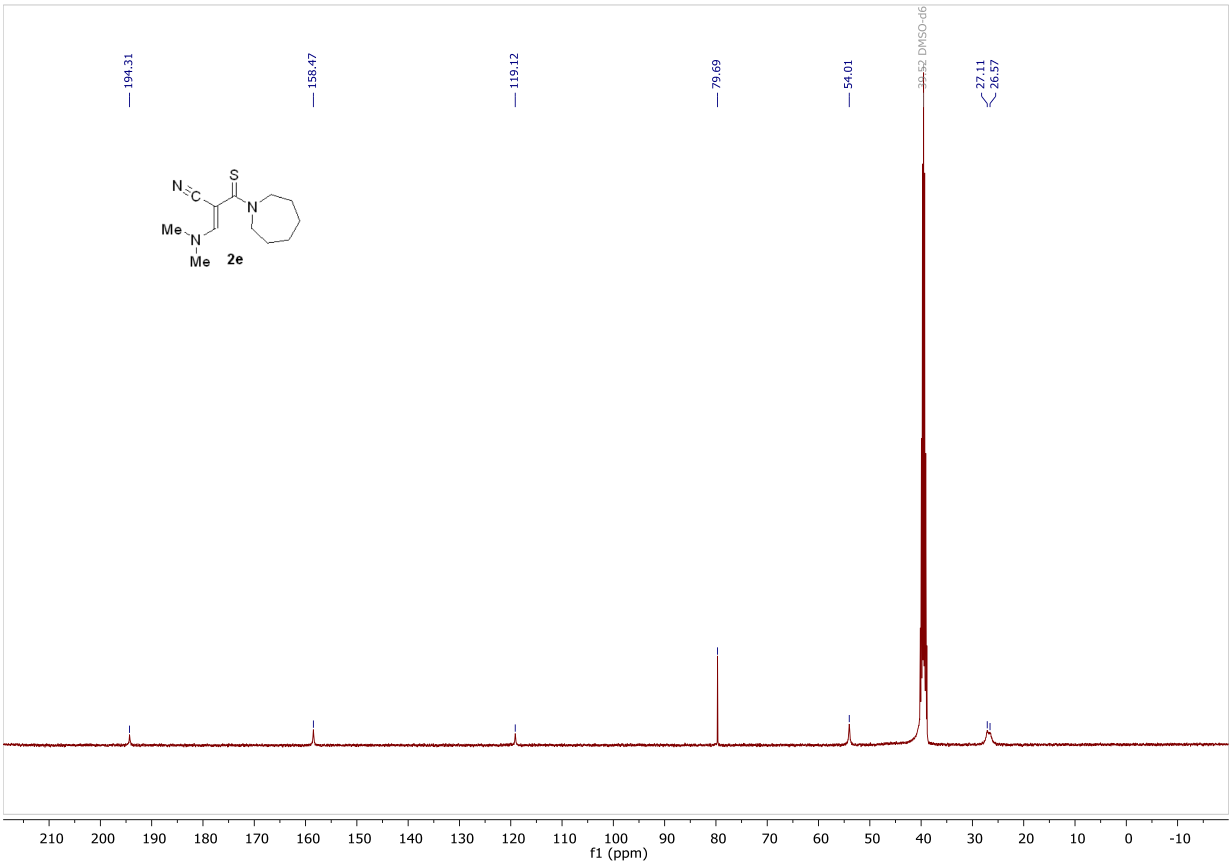

Figure S5. ^{1}H NMR spectrum (DMSO-d_6) of **4a**.

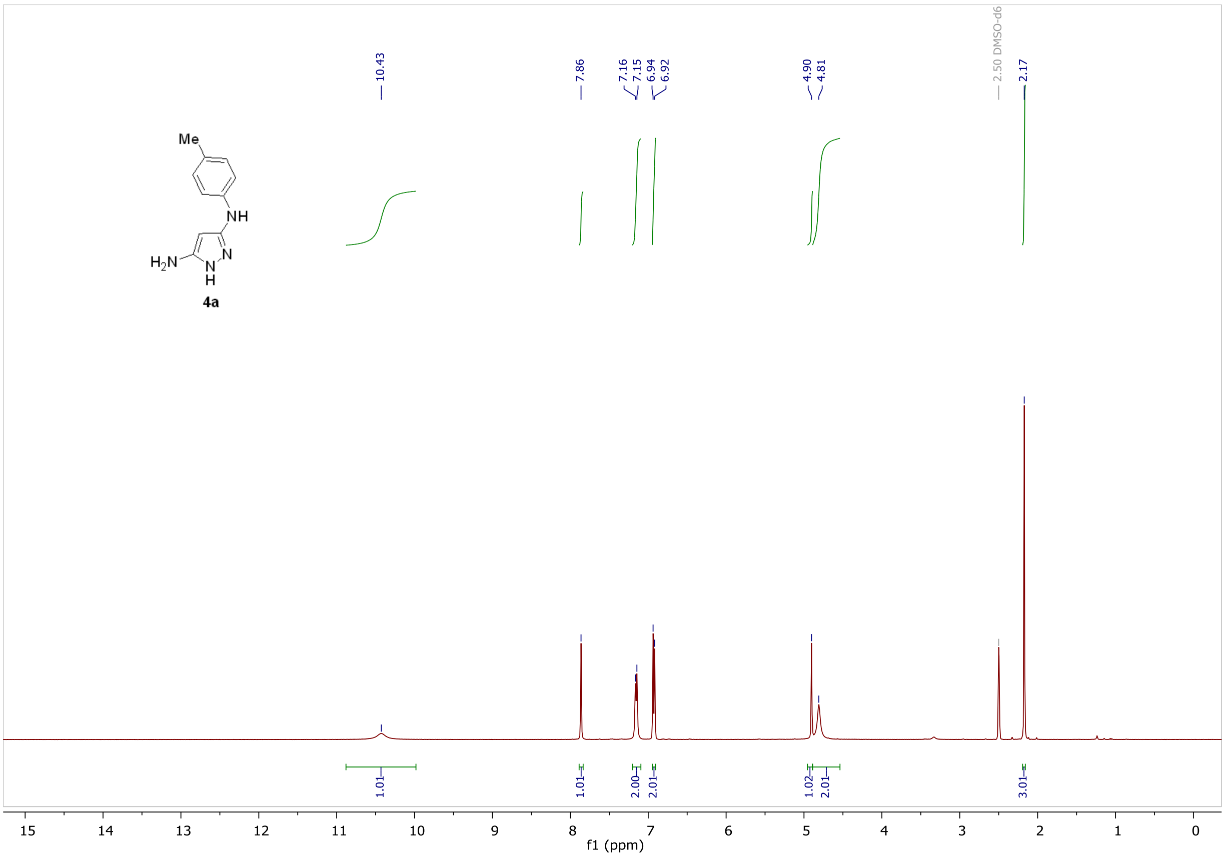

Figure S6. ^{13}C NMR spectrum (DMSO-d_6) of **4a**.

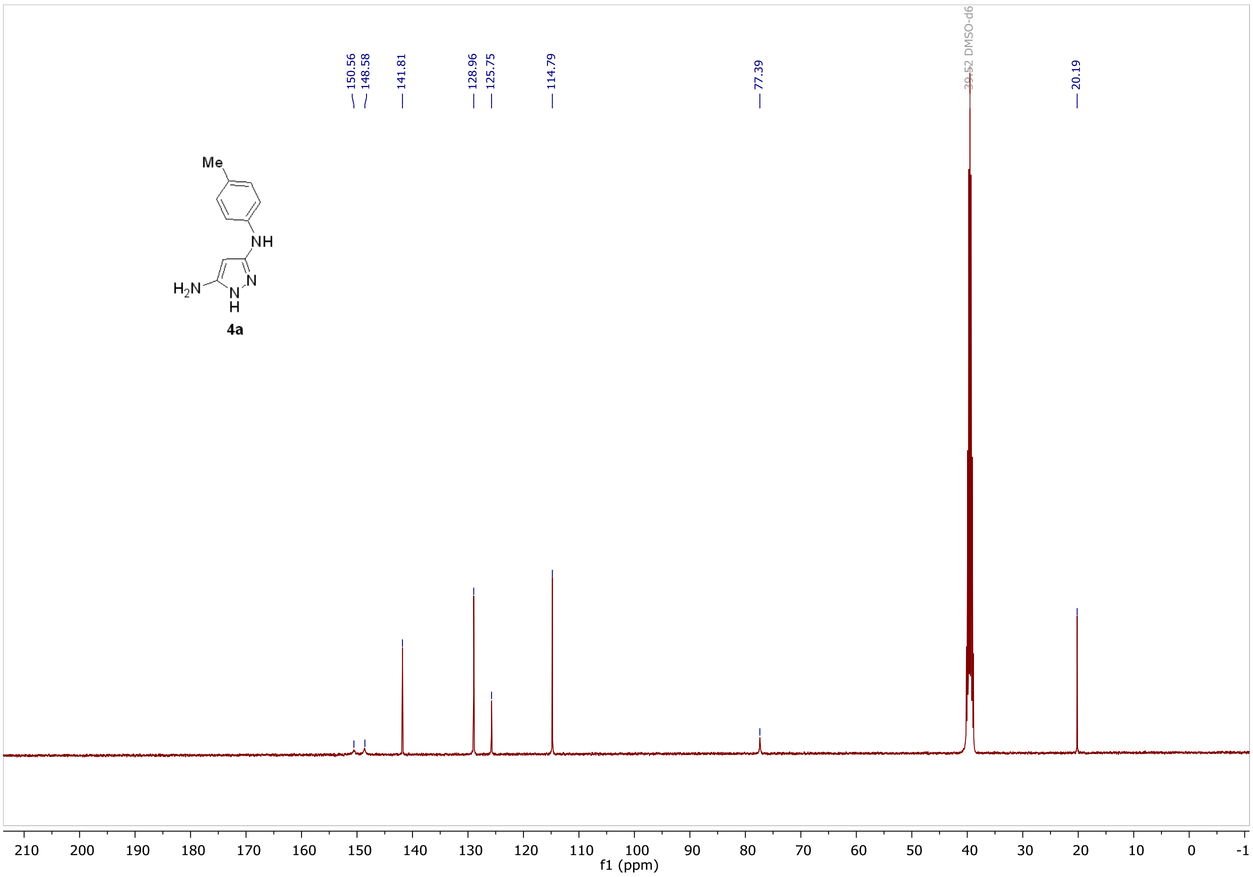

Figure S7. ^{1}H NMR spectrum (DMSO-d_6) of **4b**.

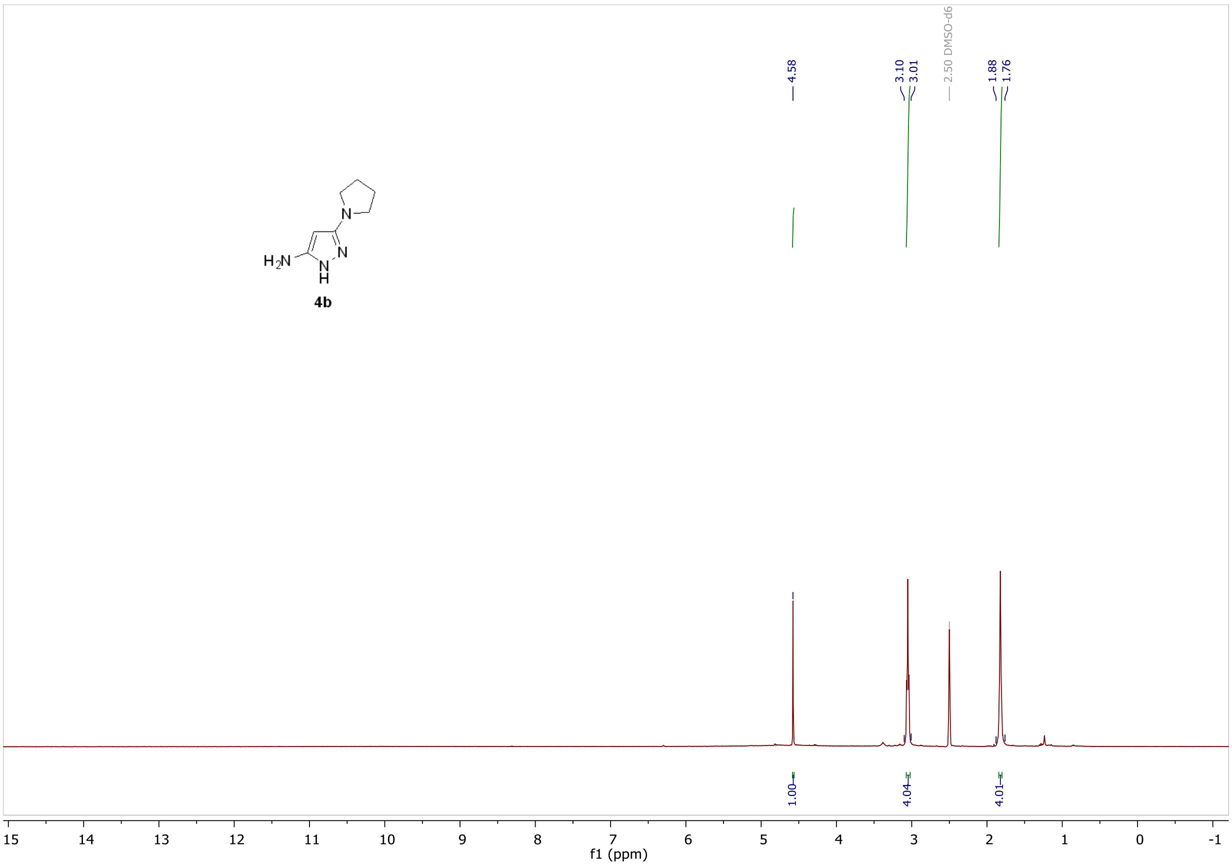

Figure S8. ^{13}C NMR spectrum (DMSO-d_6) of **4b**.

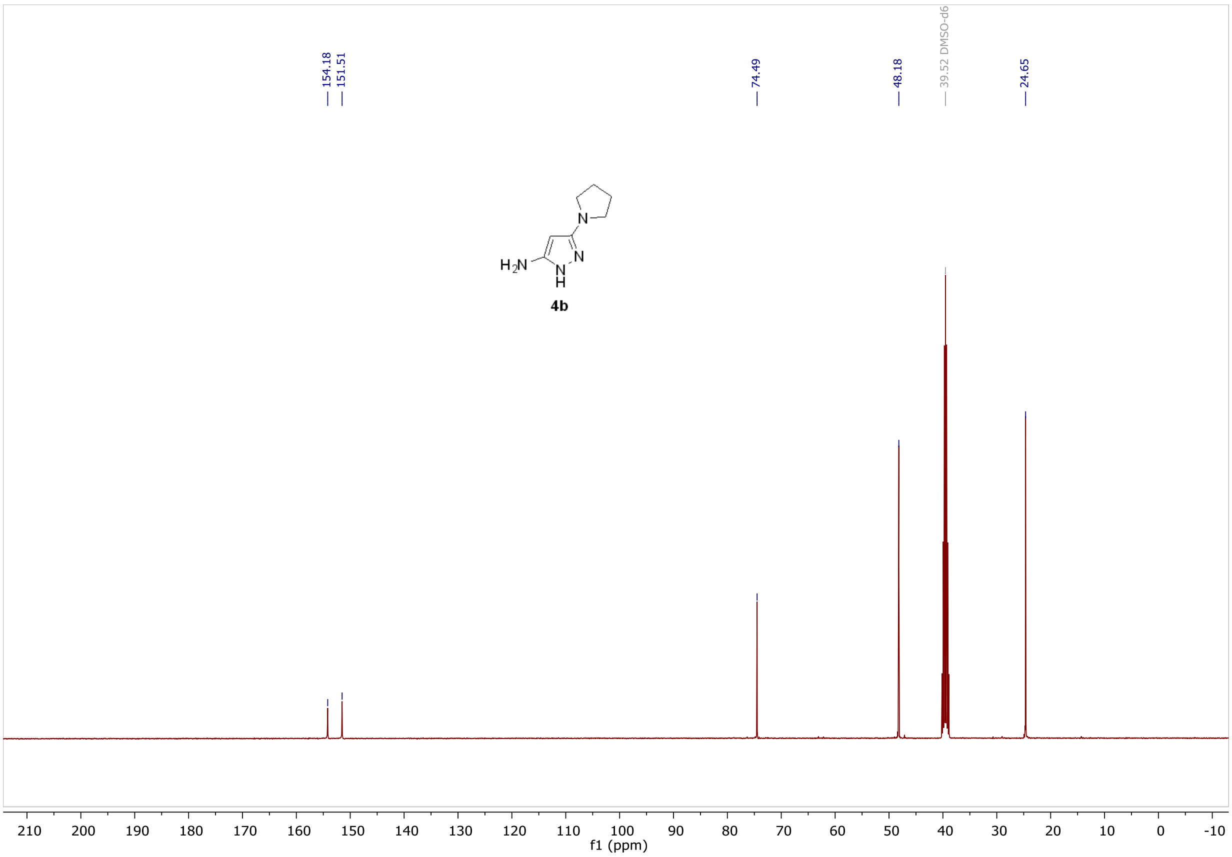

Figure S9. ^{1}H NMR spectrum (DMSO-d_6) of **4c**.

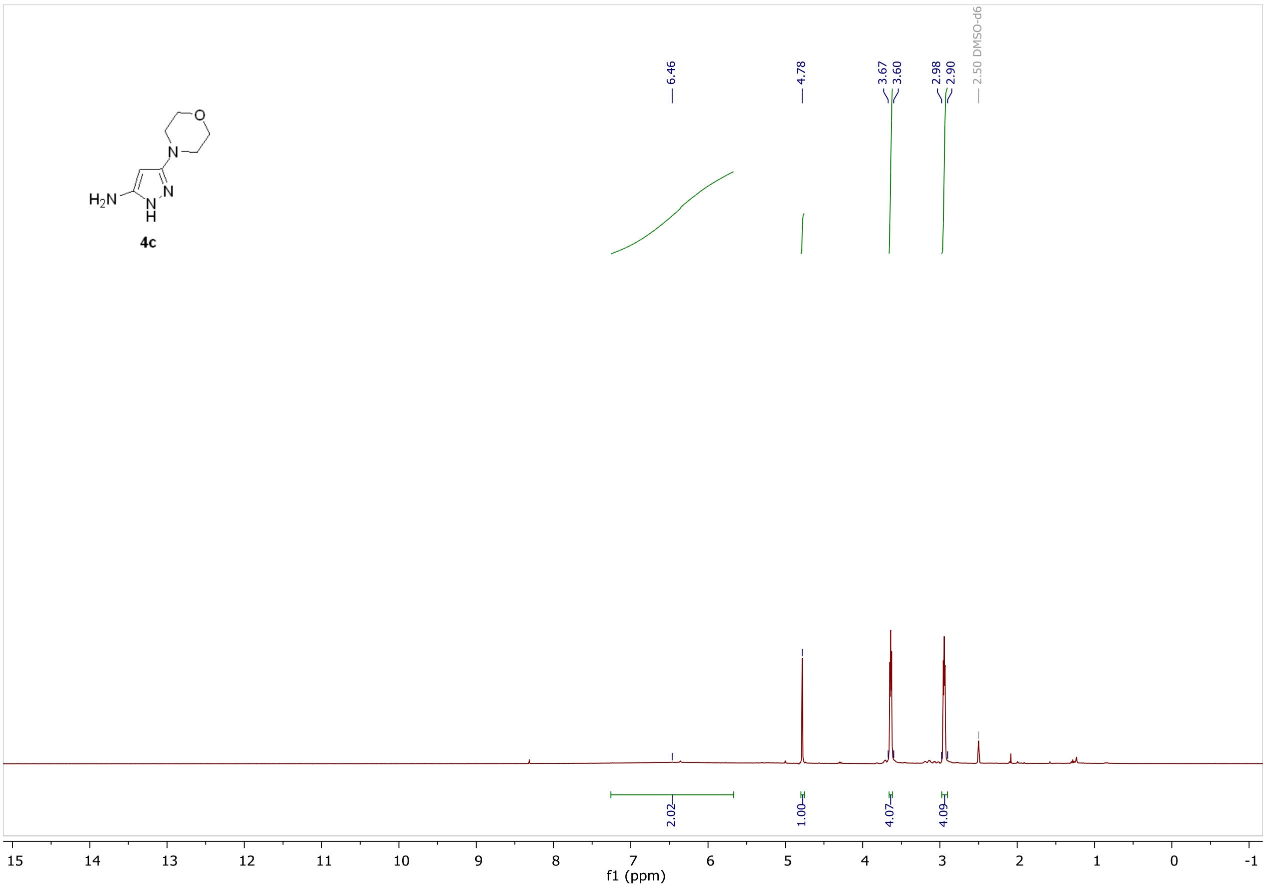

Figure S10. ^{1}H NMR spectrum ($CDCl_3$) of **4c**.

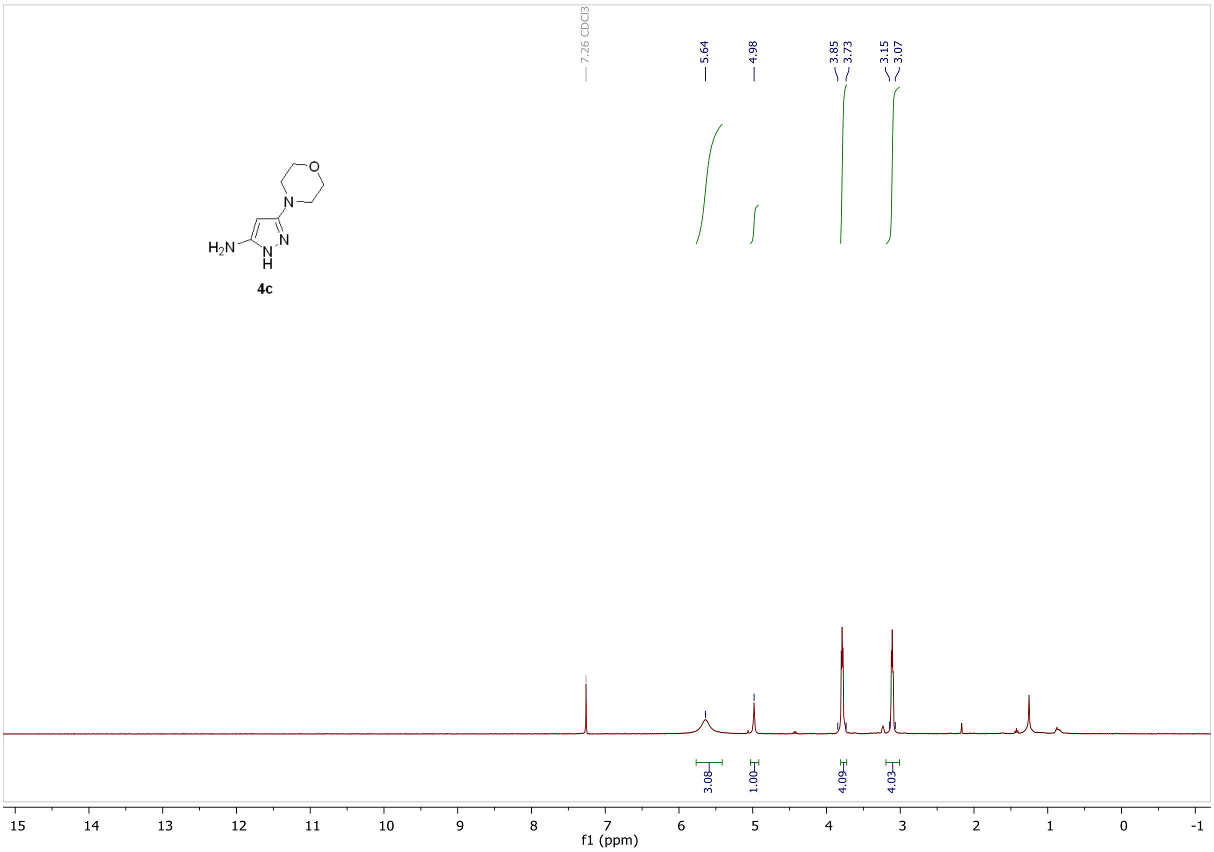

Figure S11. ^{13}C NMR spectrum (DMSO-d_6) of **4c**.

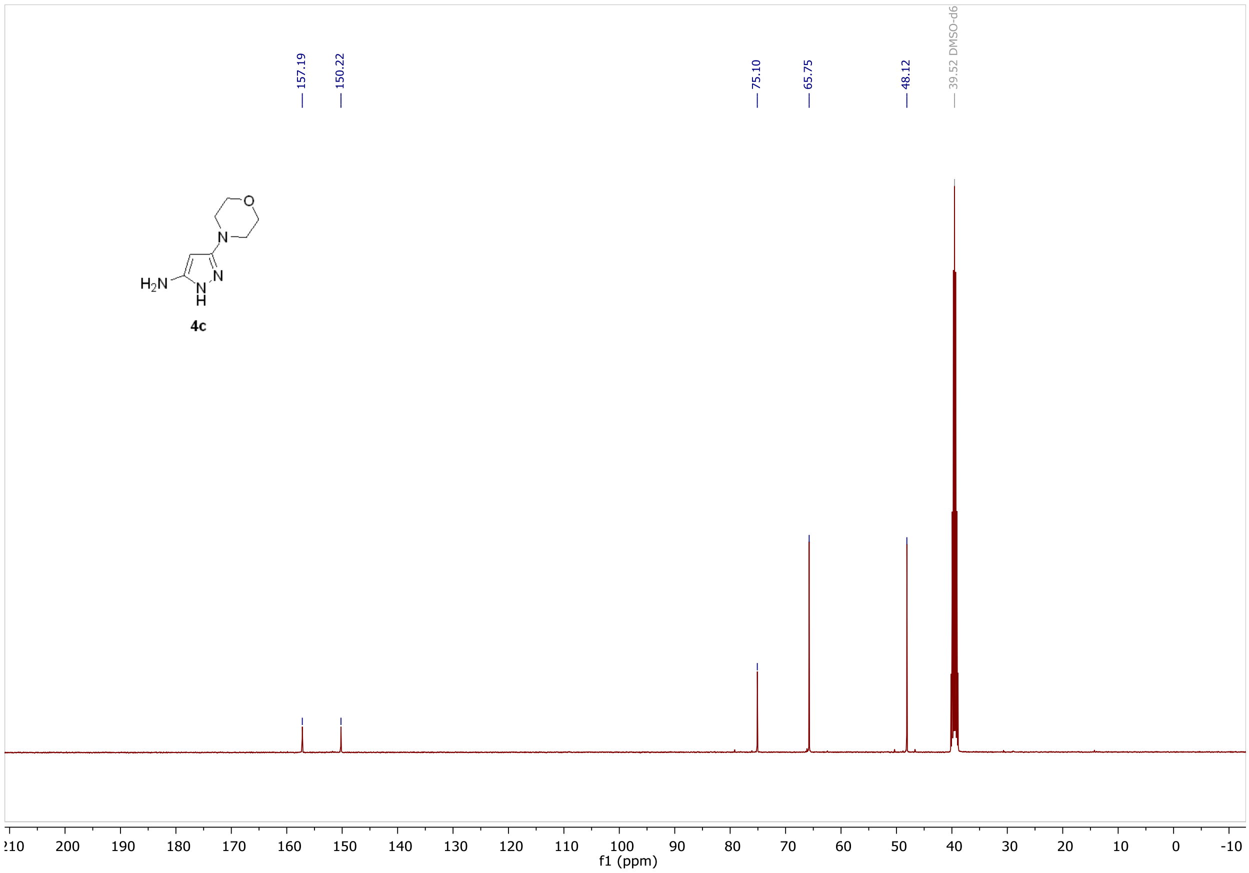

Figure S12. ^{1}H NMR spectrum (DMSO-d_6) of **5a**.

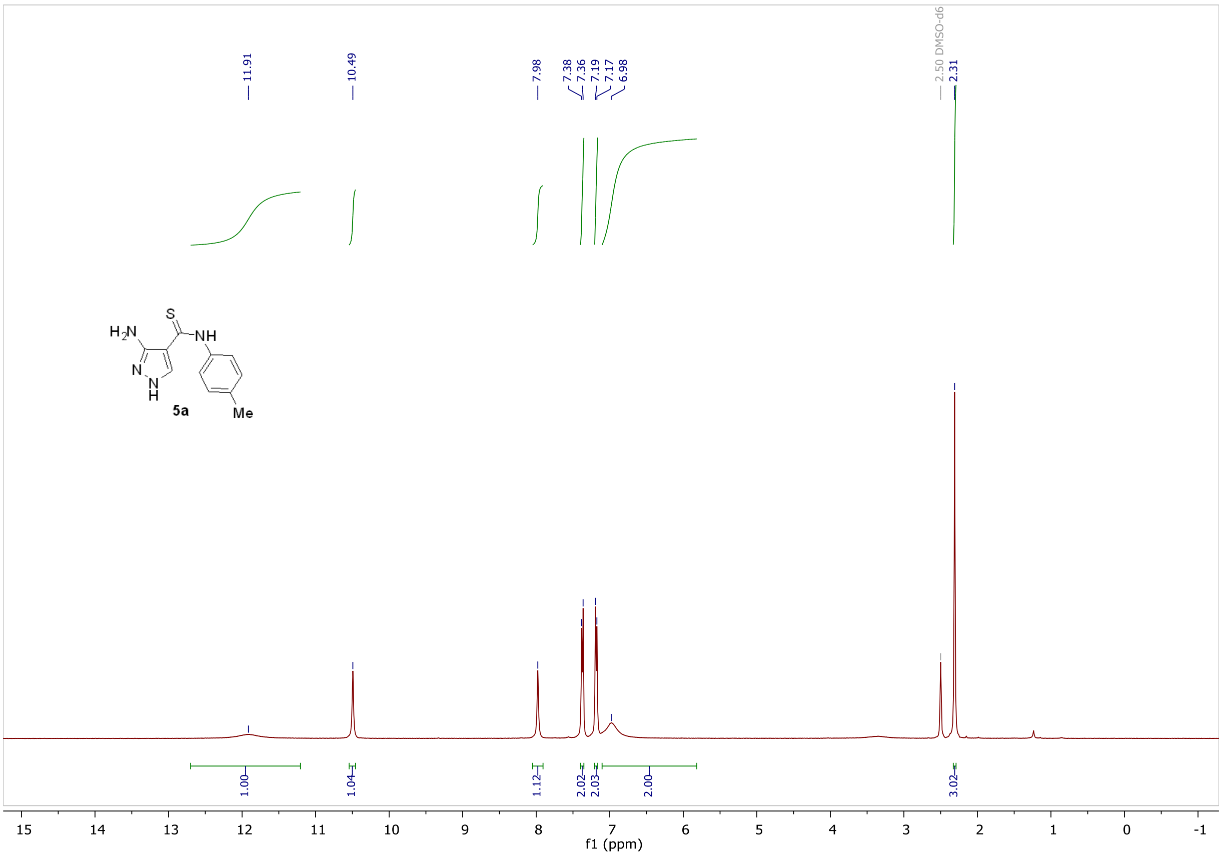

Figure S13. ^{13}C NMR spectrum (DMSO-d_6) of **5a**.

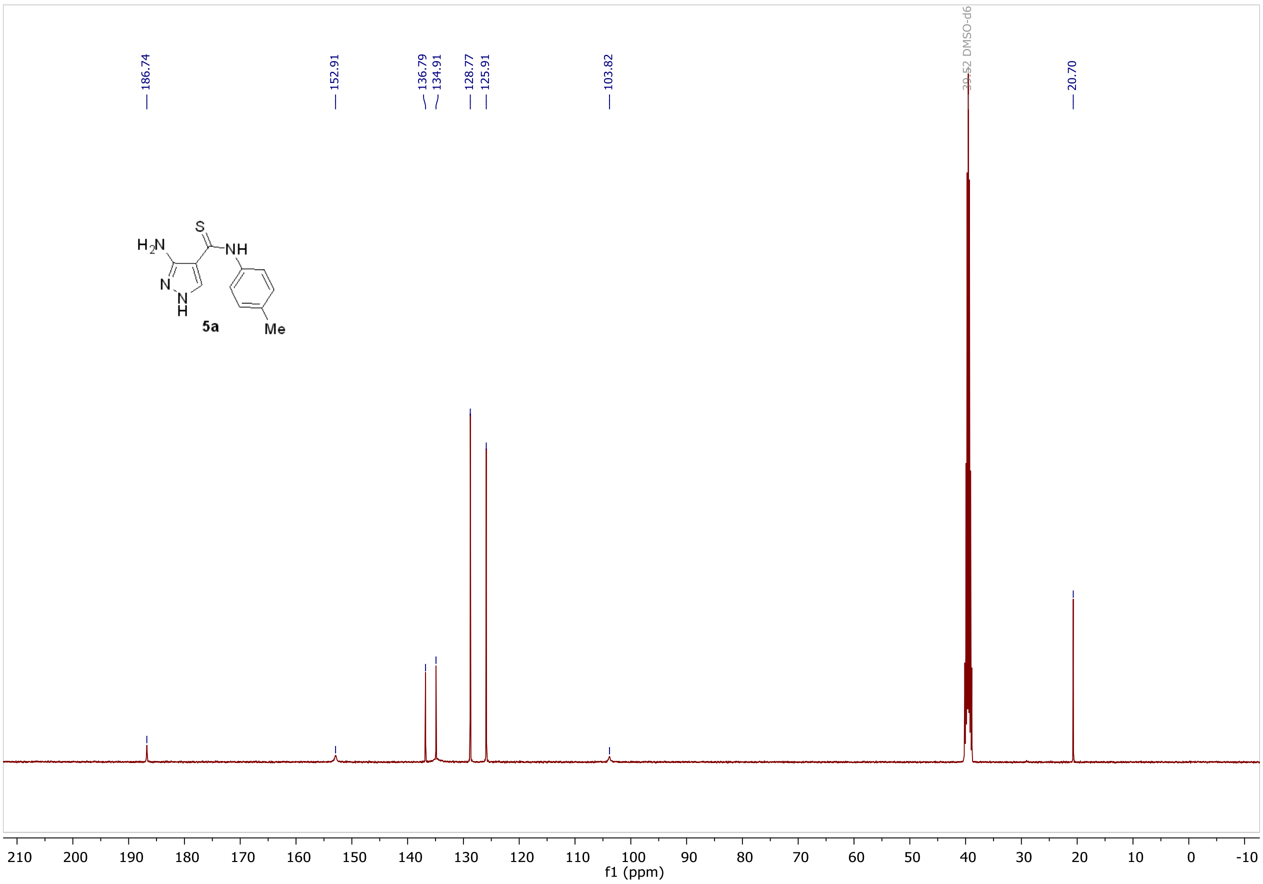

Figure S14. ^{1}H NMR spectrum (DMSO-d_6) of **5b**.

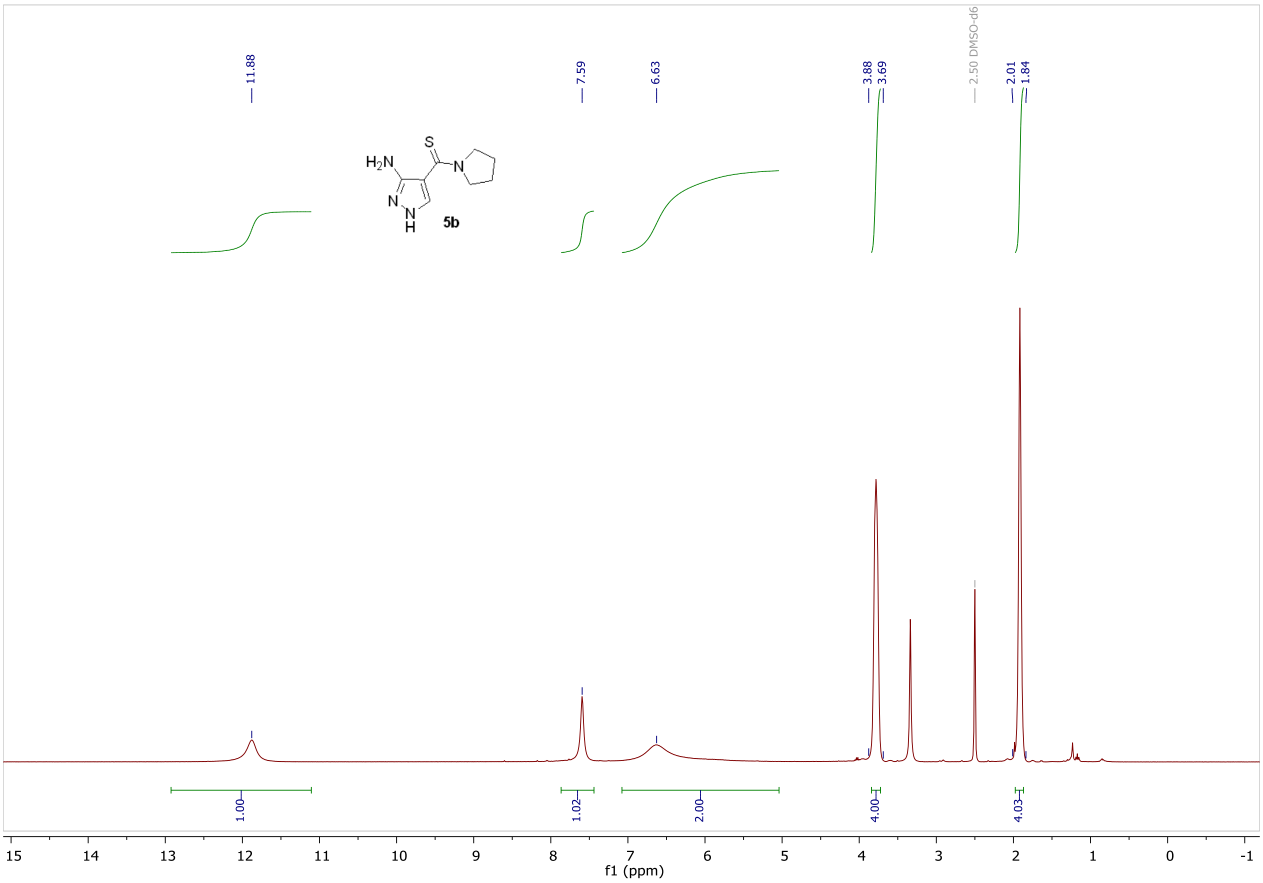

Figure S15. ^{13}C NMR spectrum (DMSO-d_6) of **5b**.

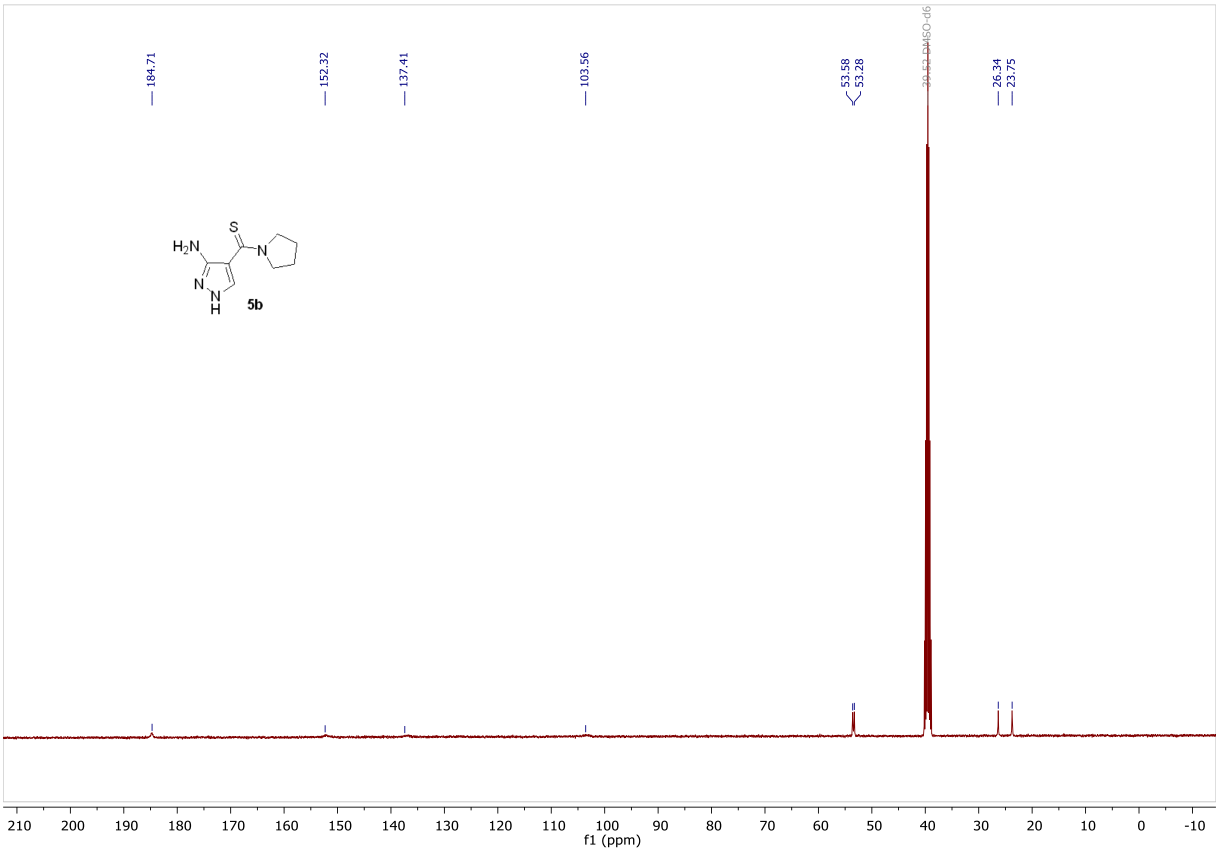

Figure S16. ^{1}H NMR spectrum (DMSO-d_6) of **5c**.

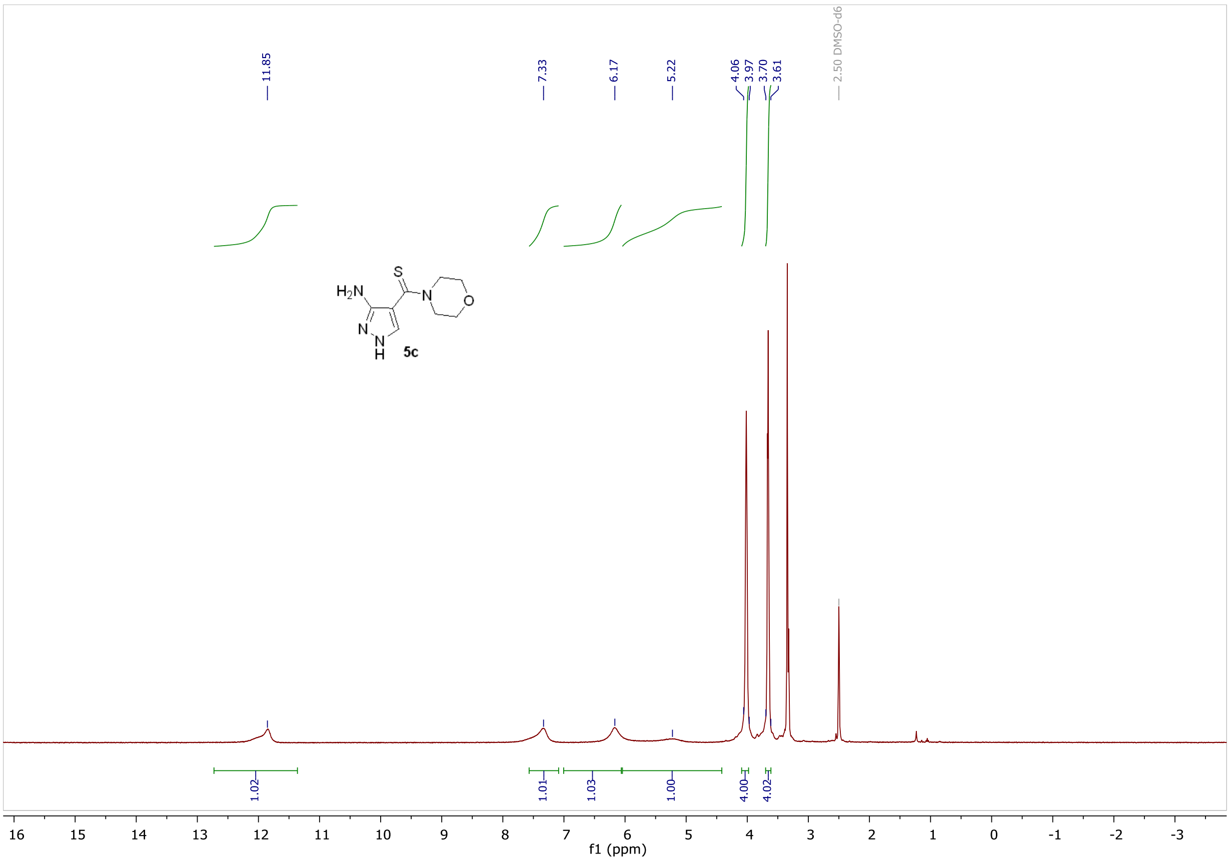

Figure S17. ^{13}C NMR spectrum (DMSO-d_6) of **5c**.

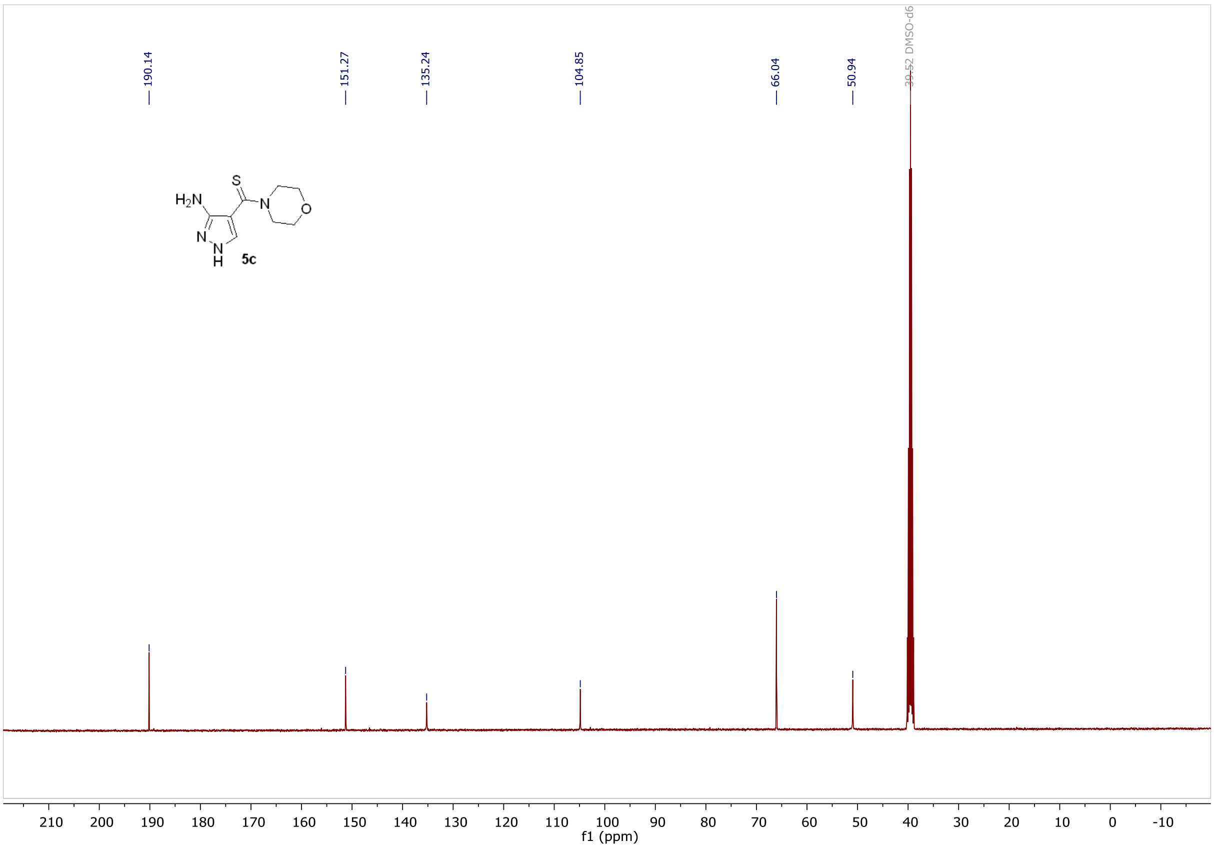

Figure S18. ^{1}H NMR spectrum (DMSO-d_6) of **5d**.

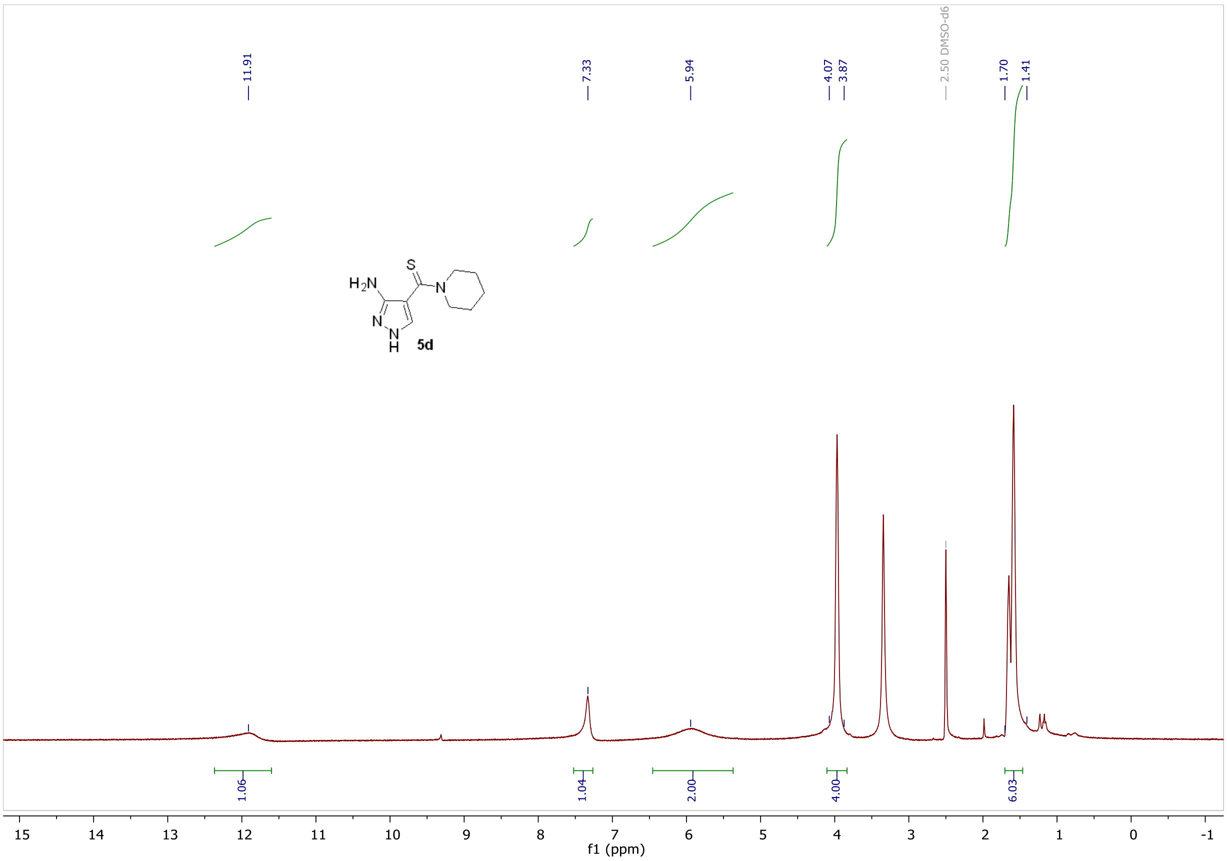

Figure S19. ^{13}C NMR spectrum (DMSO-d_6) of **5d**.

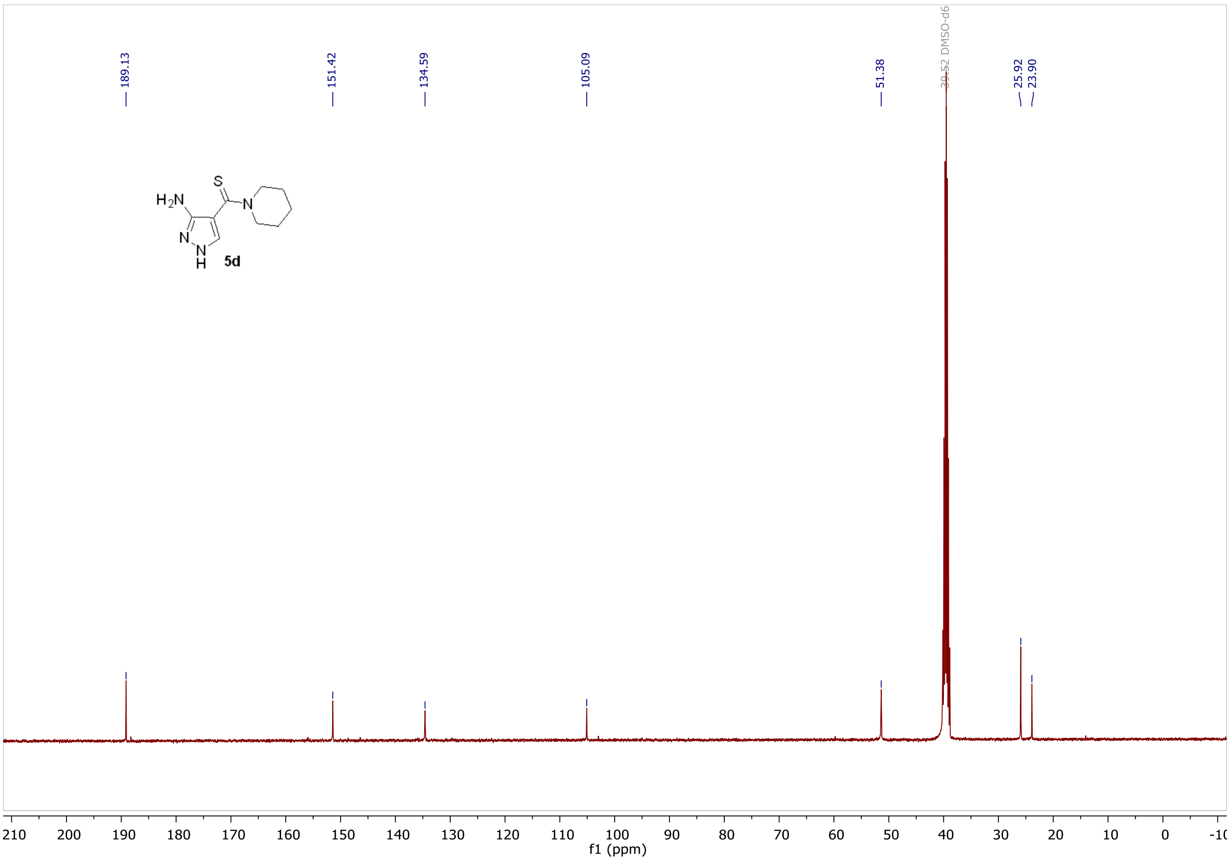

Figure S20. ^{1}H NMR spectrum (DMSO-d_6) of **5e**.

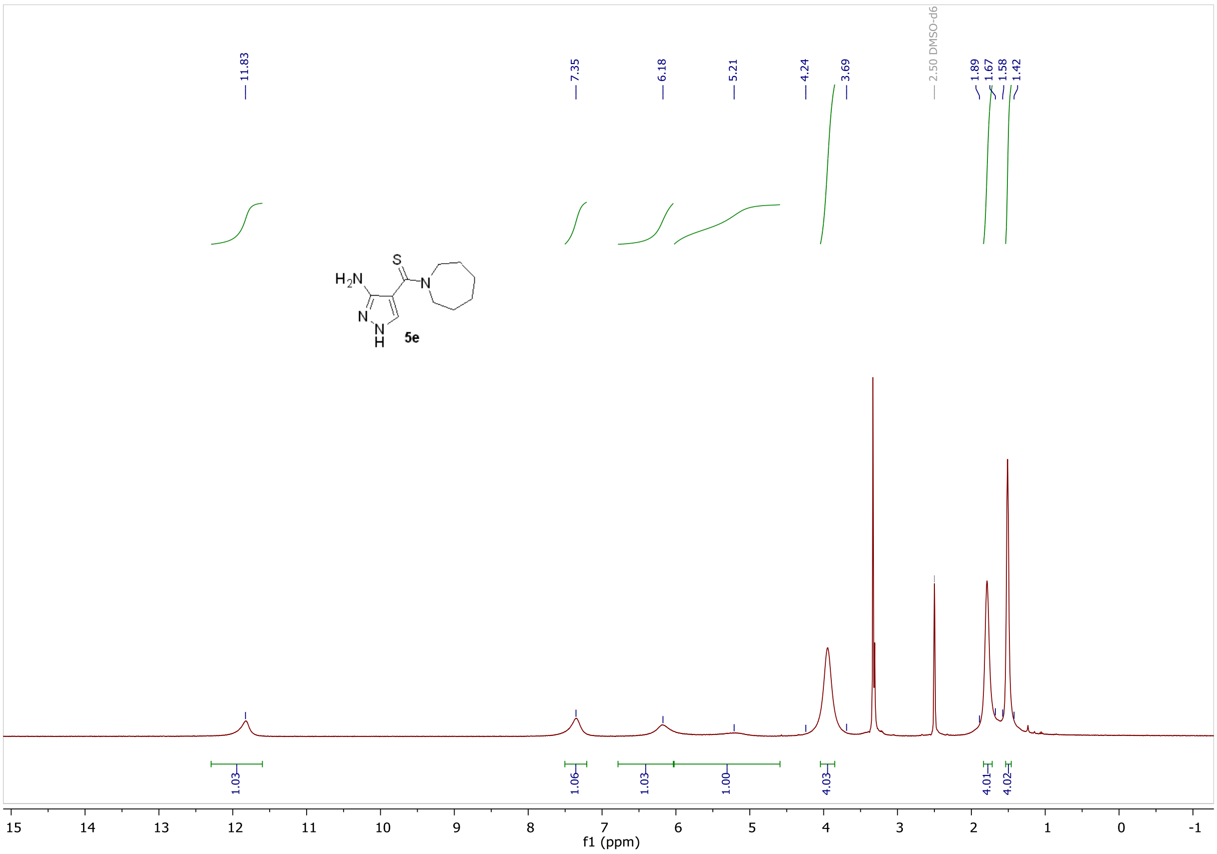

Figure S21. ^{13}C NMR spectrum (DMSO-d_6) of **5e**.

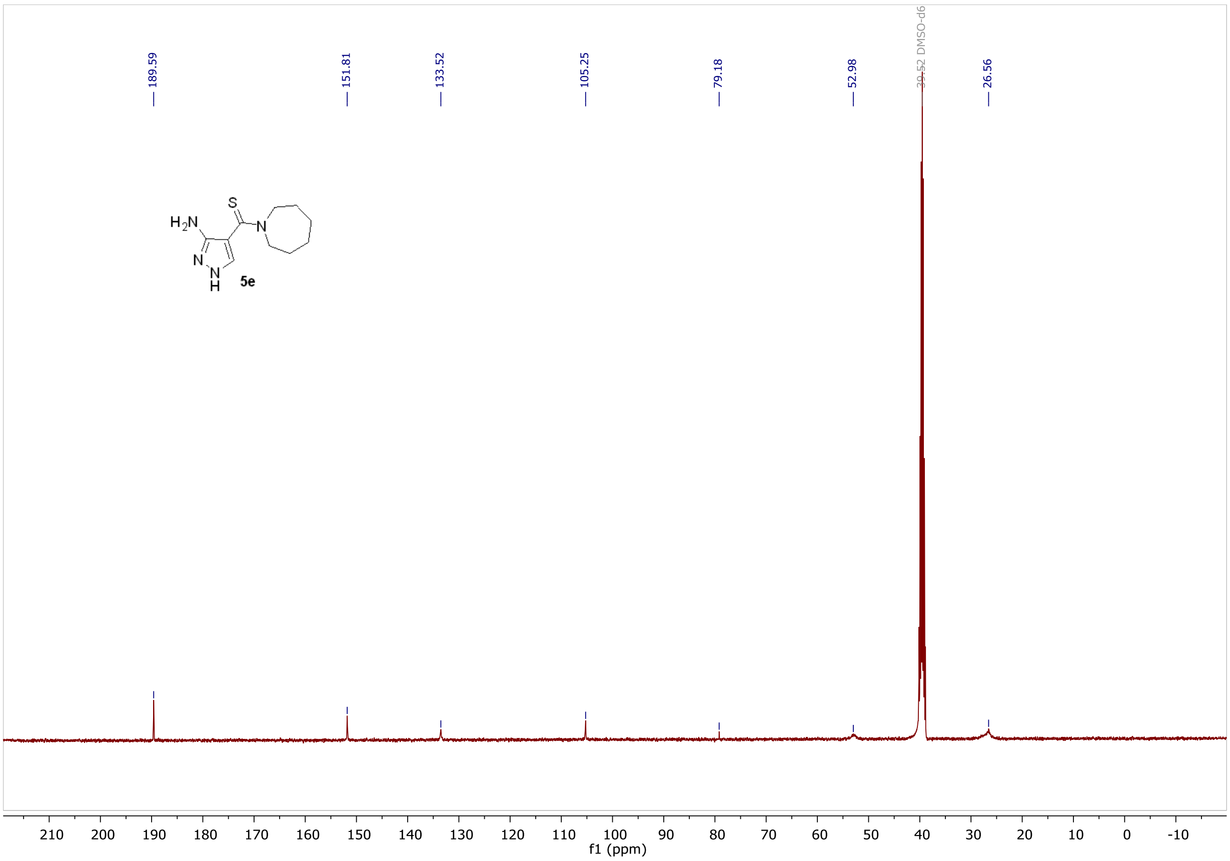

Figure S22. ^{1}H NMR spectrum (DMSO-d_6) of **6a**.

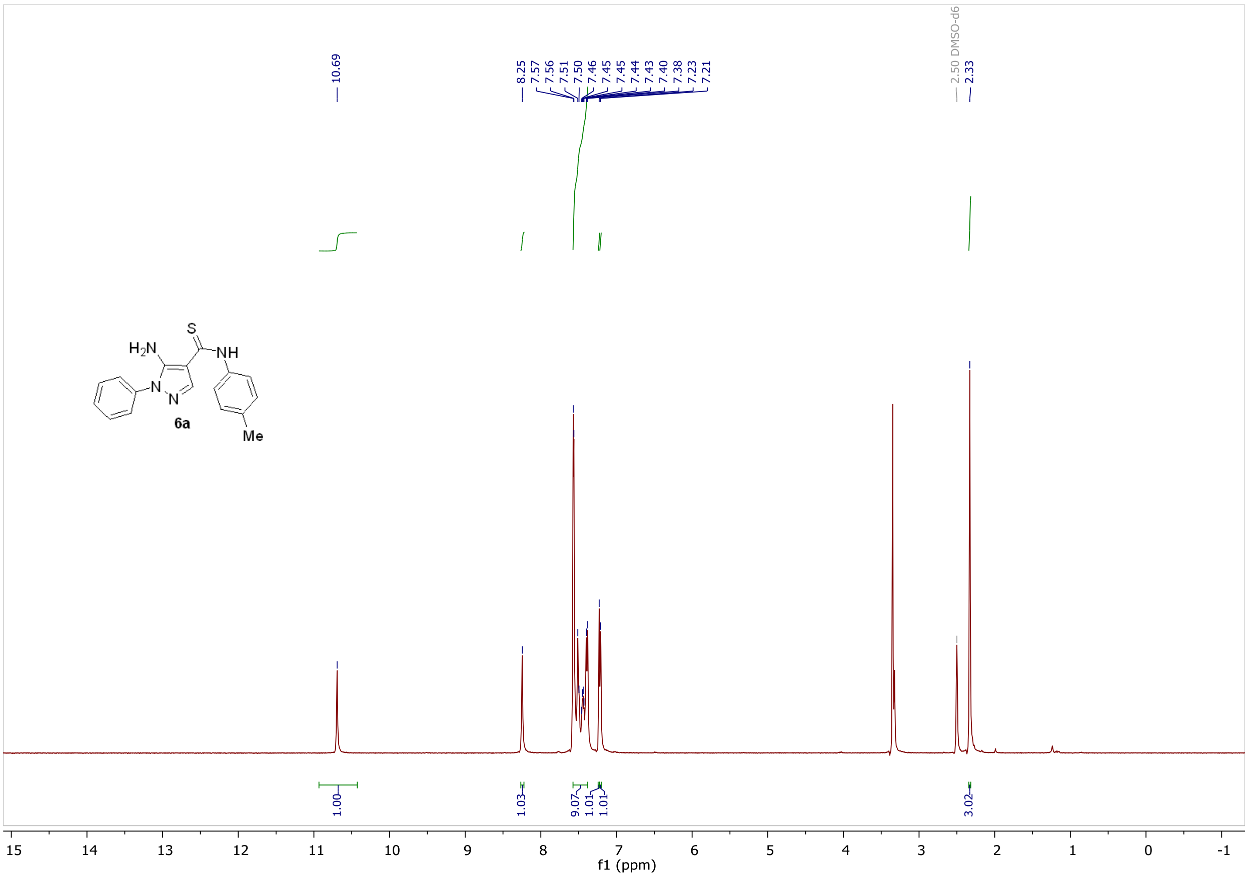

Figure S23. ^{13}C NMR spectrum (DMSO-d_6) of **6a**.

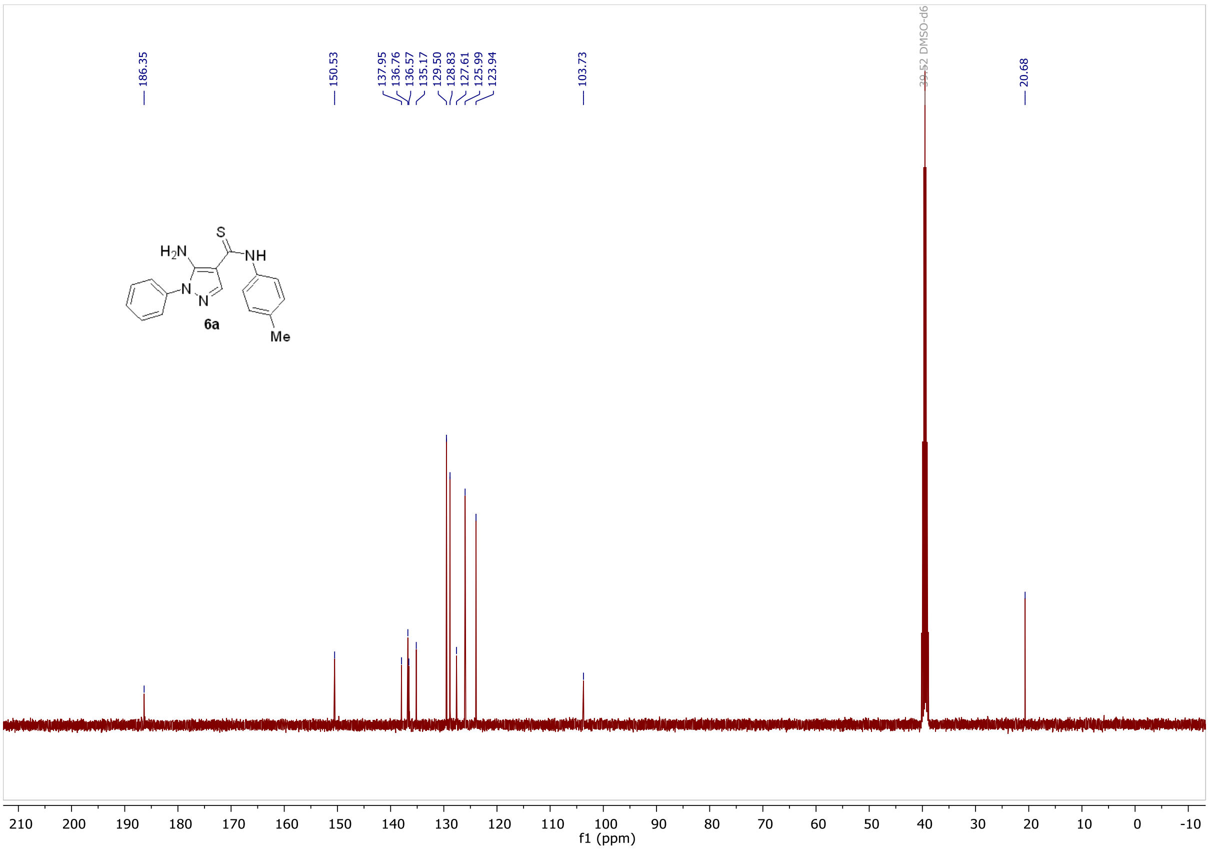

Figure S24. ^{1}H NMR spectrum (DMSO-d_6) of **6b**.

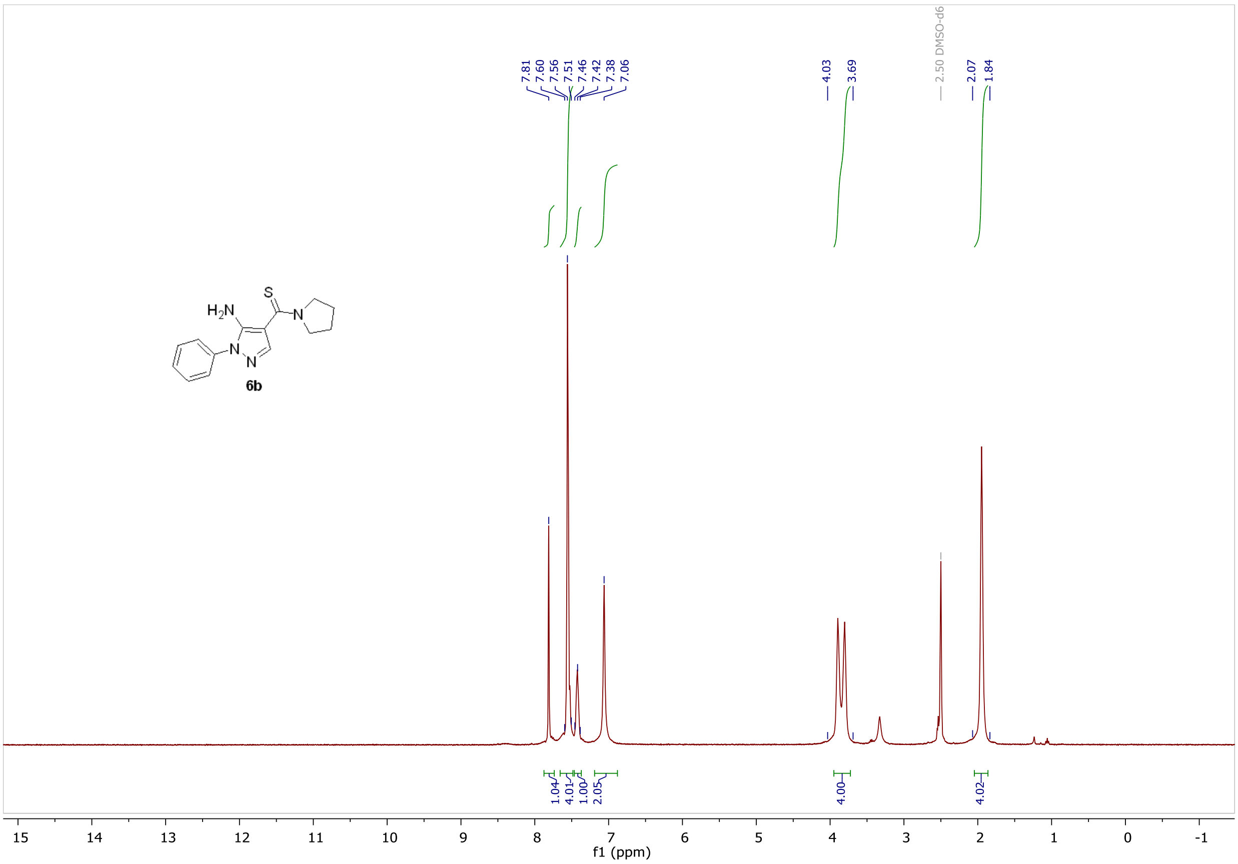

Figure S25. ^{13}C NMR spectrum (DMSO-d_6) of **6b**.

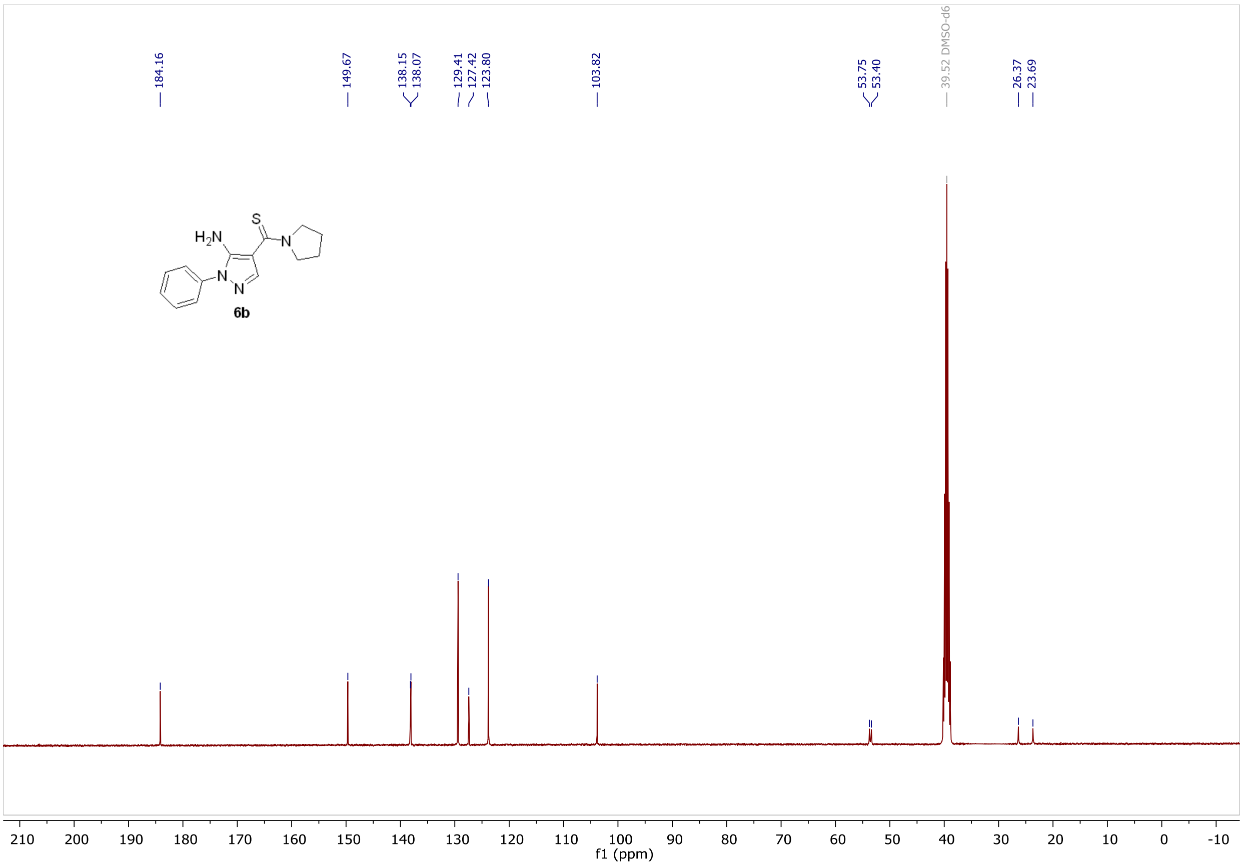

Figure S26. ^{1}H NMR spectrum (DMSO-d_6) of **6c**.

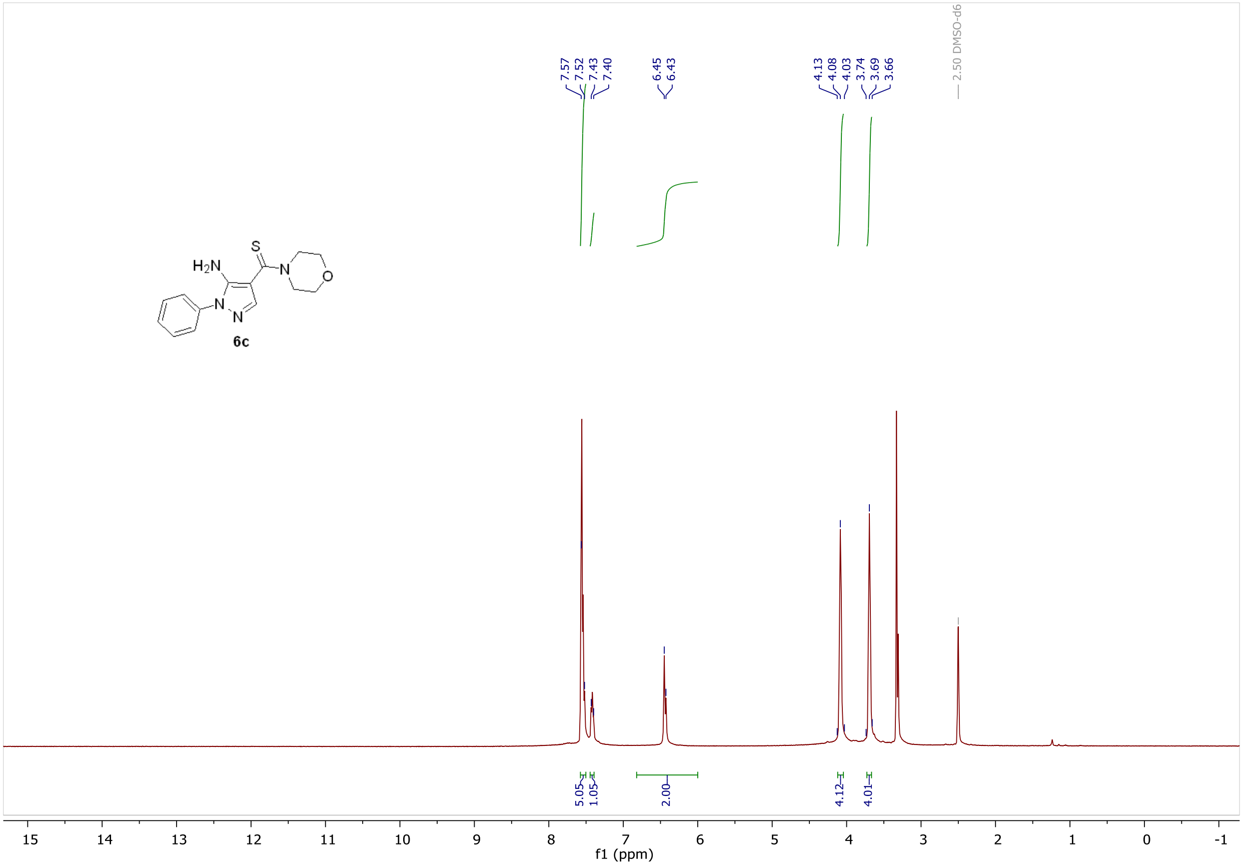

Figure S27. ^{13}C NMR spectrum (DMSO-d_6) of **6c**.

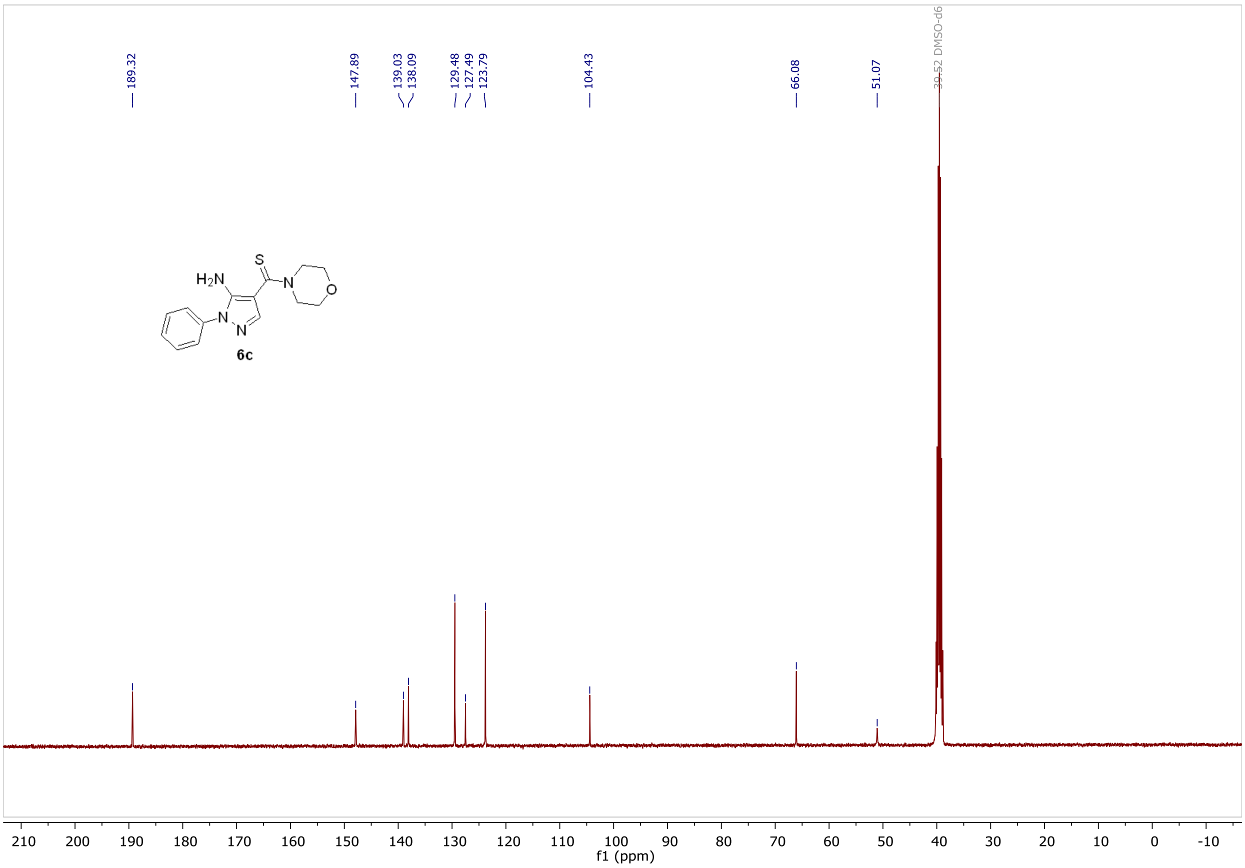

Figure S28. ^{1}H NMR spectrum (DMSO-d_6) of **6d**.

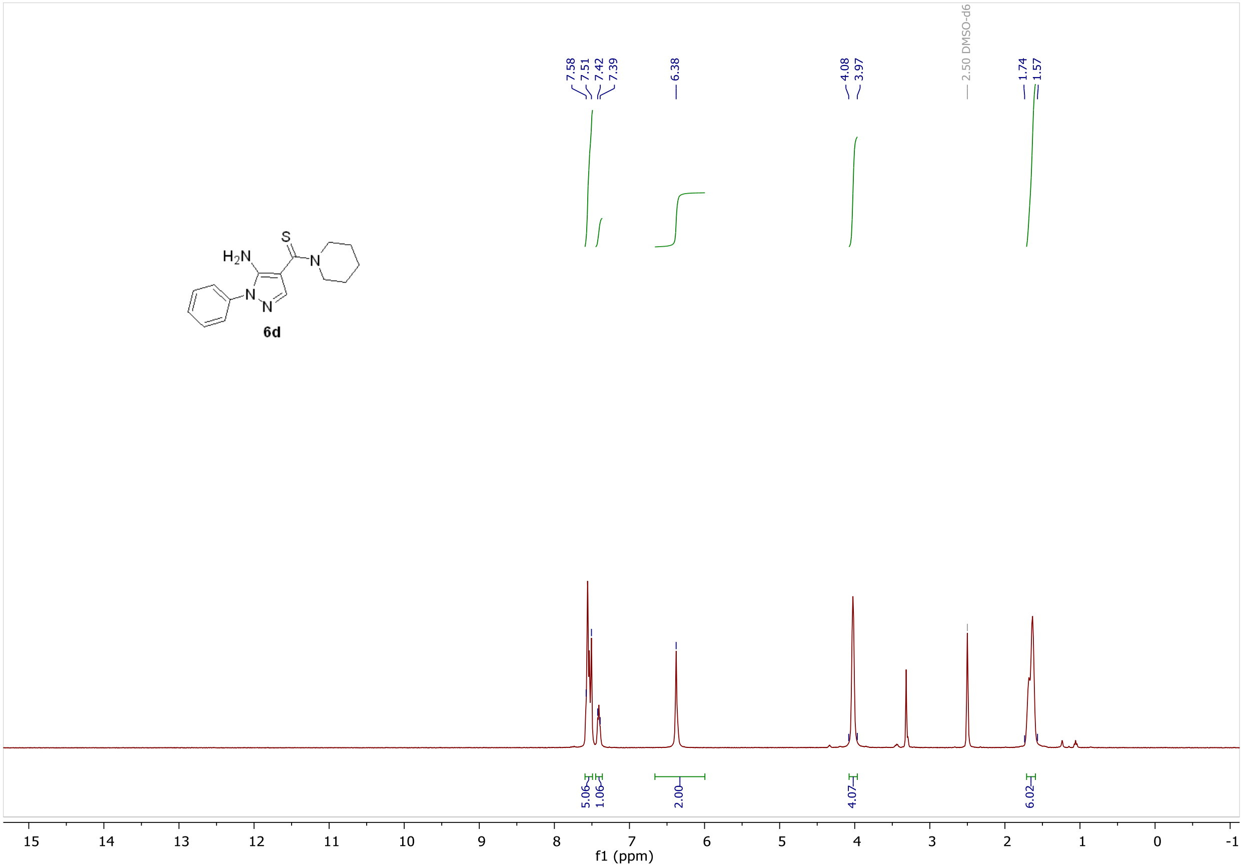

Figure S29. ^{13}C NMR spectrum (DMSO-d_6) of **6d**.

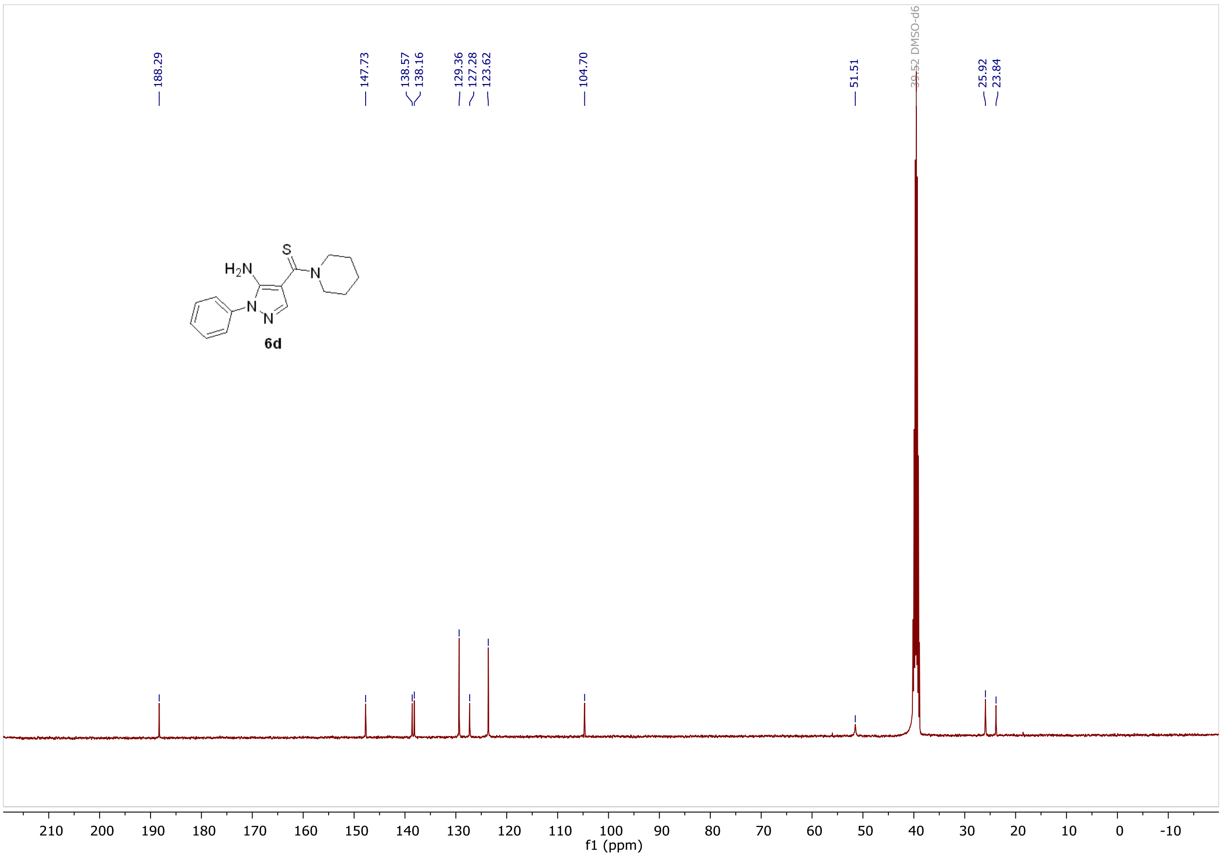

Figure S30. ^{1}H NMR spectrum (DMSO-d_6) of **6e**.

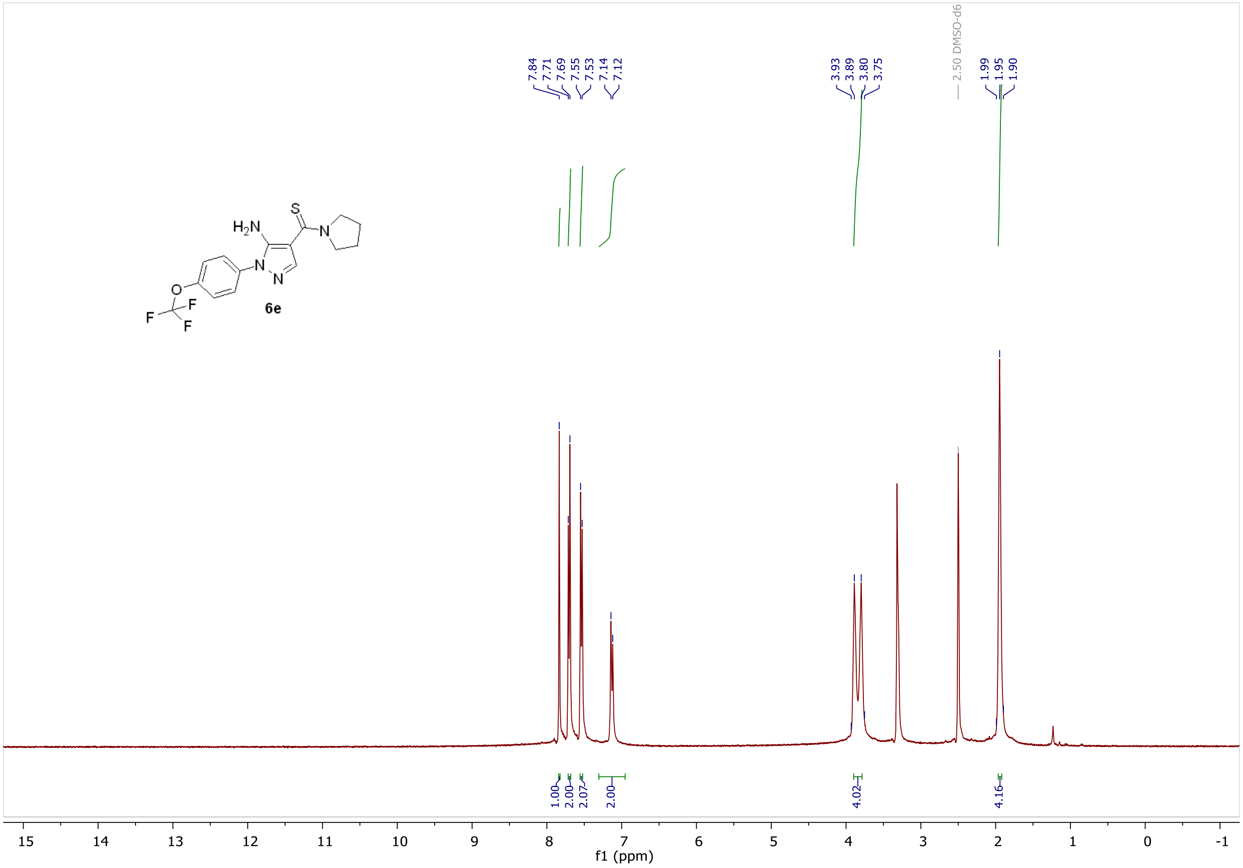

Figure S31. ^{13}C NMR spectrum (DMSO-d_6) of **6e**.

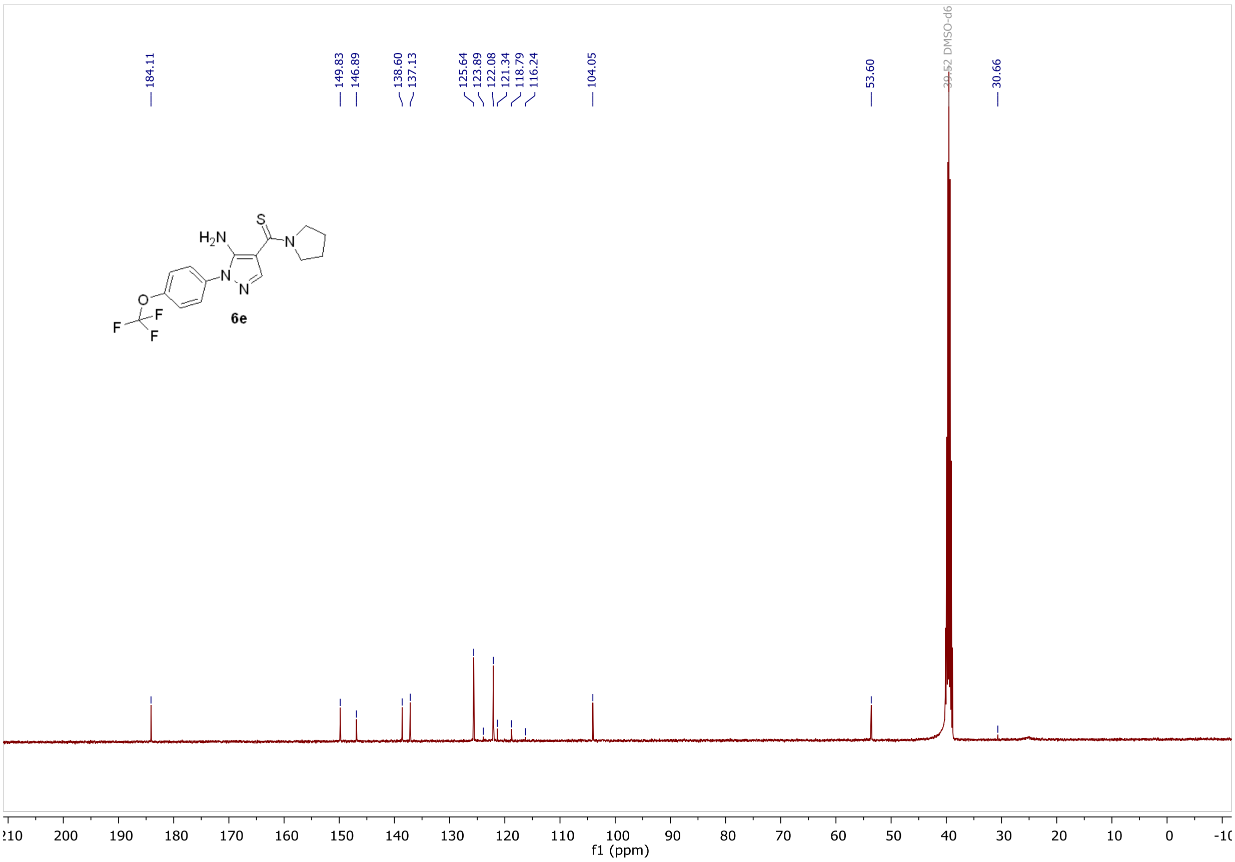

Figure S32. ^{19}F NMR spectrum (DMSO-d_6) of **6e**.

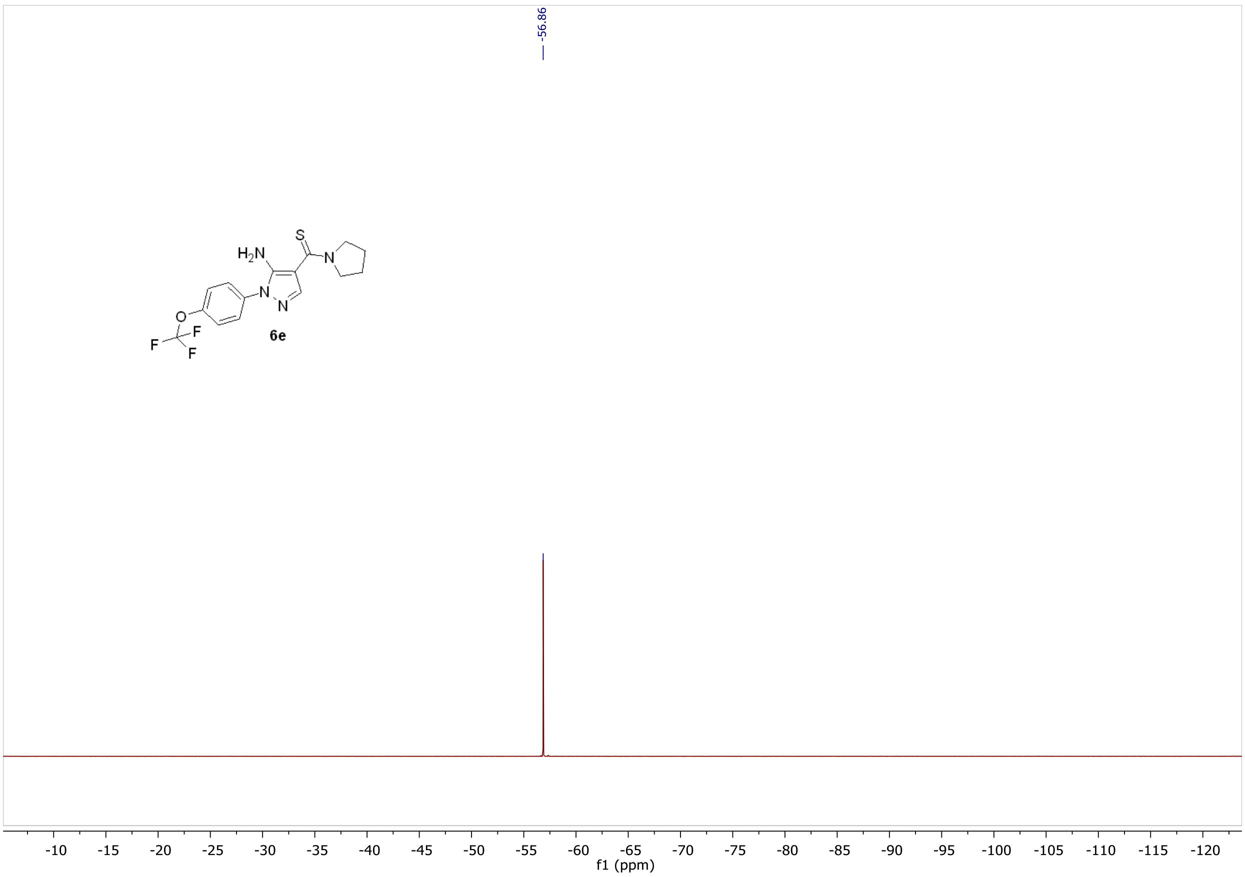

Figure S33. ^{1}H NMR spectrum (DMSO-d_6) of **6f**.

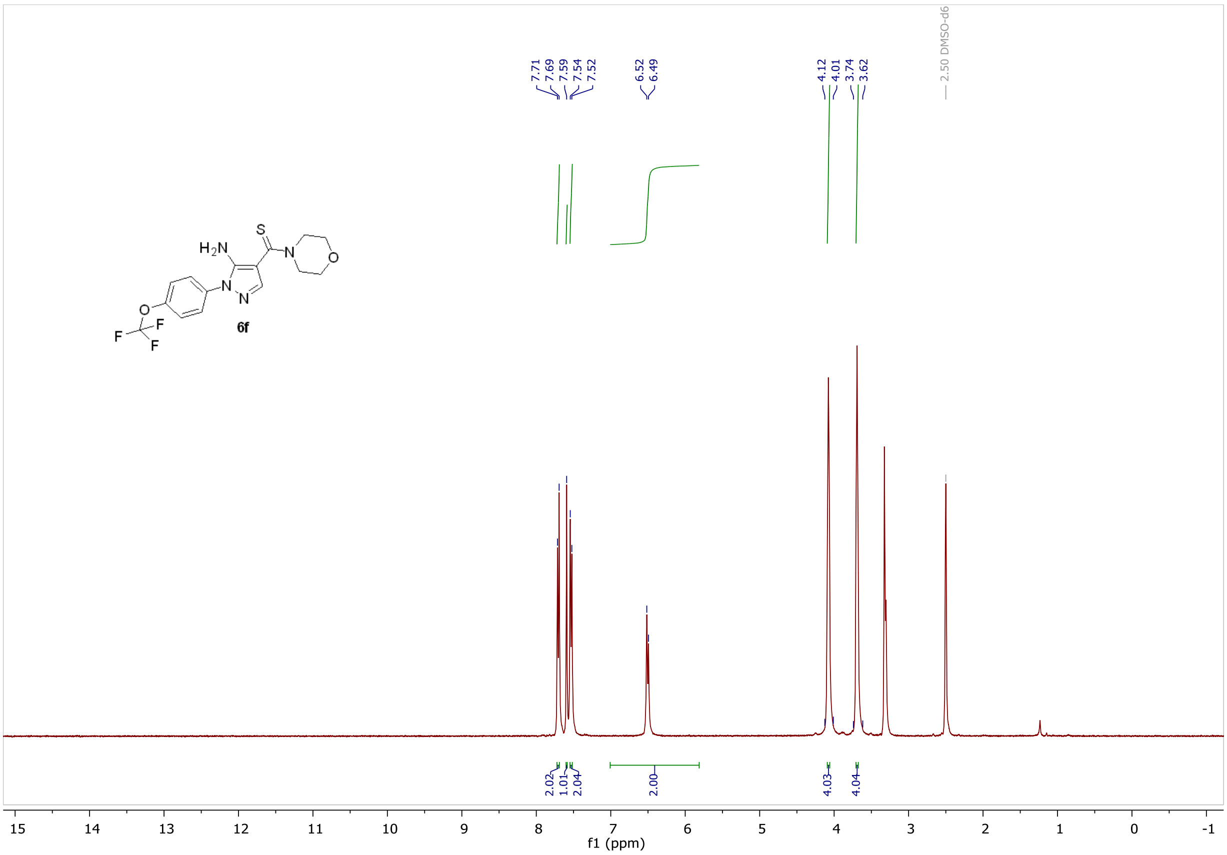

Figure S34. ^{13}C NMR spectrum (DMSO-d_6) of **6f**.

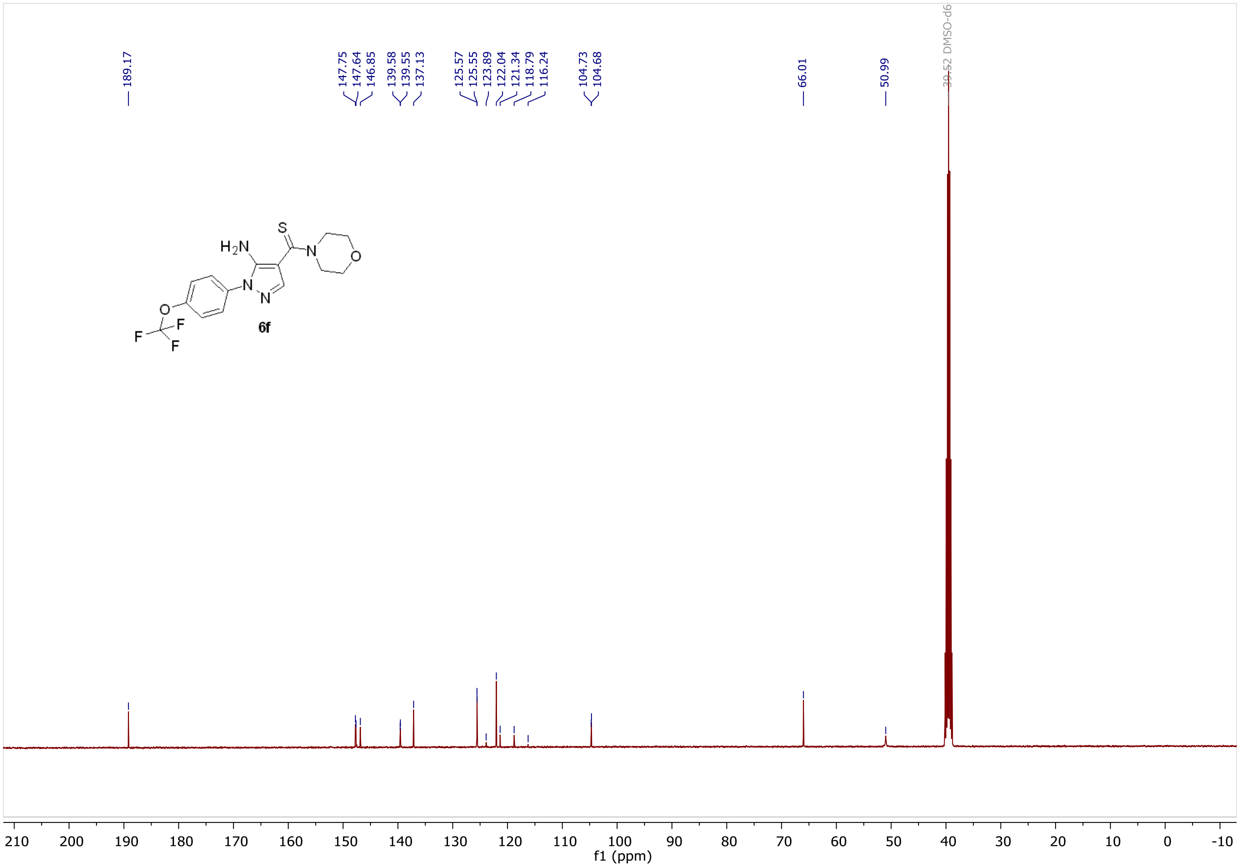

Figure S35. ^{19}F NMR spectrum (DMSO-d_6) of **6f**.

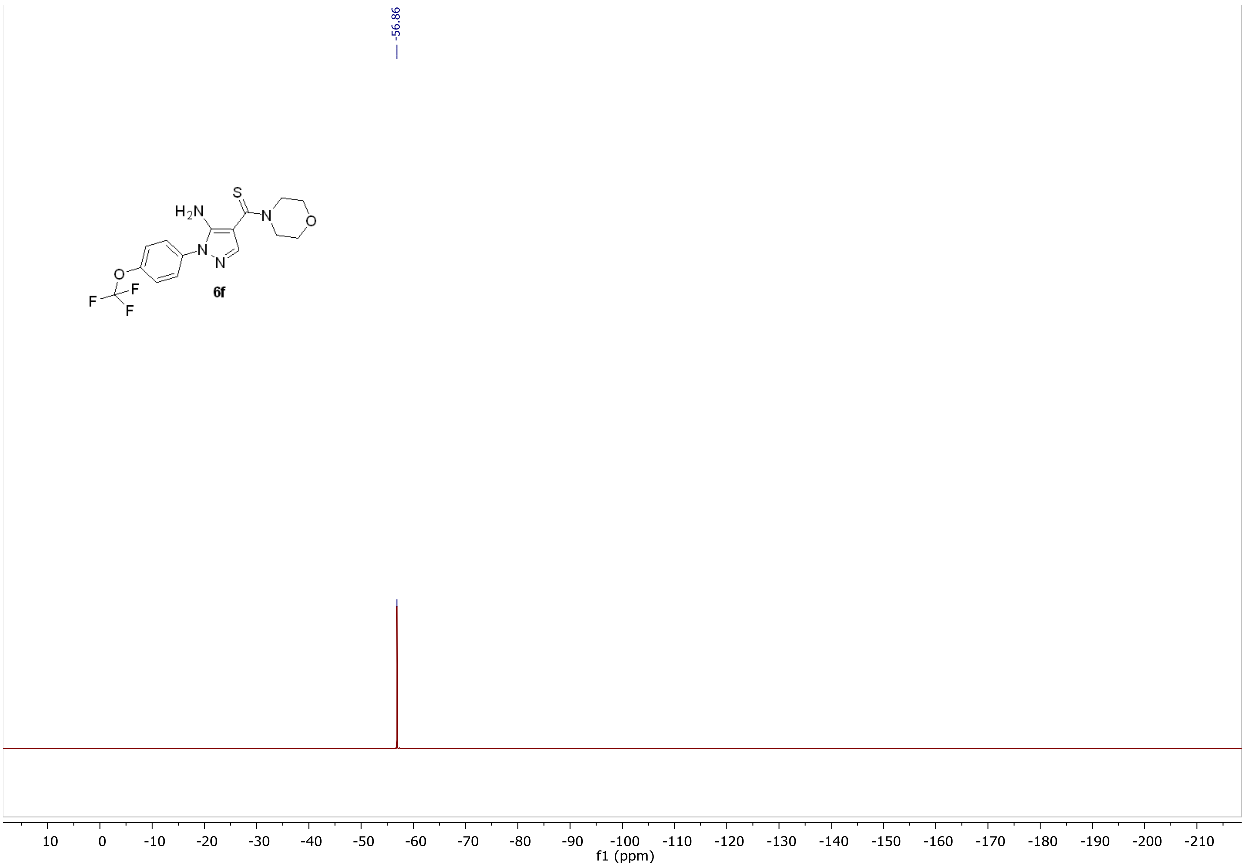

Figure S36. ^{1}H NMR spectrum (DMSO-d_6) of **7a**.

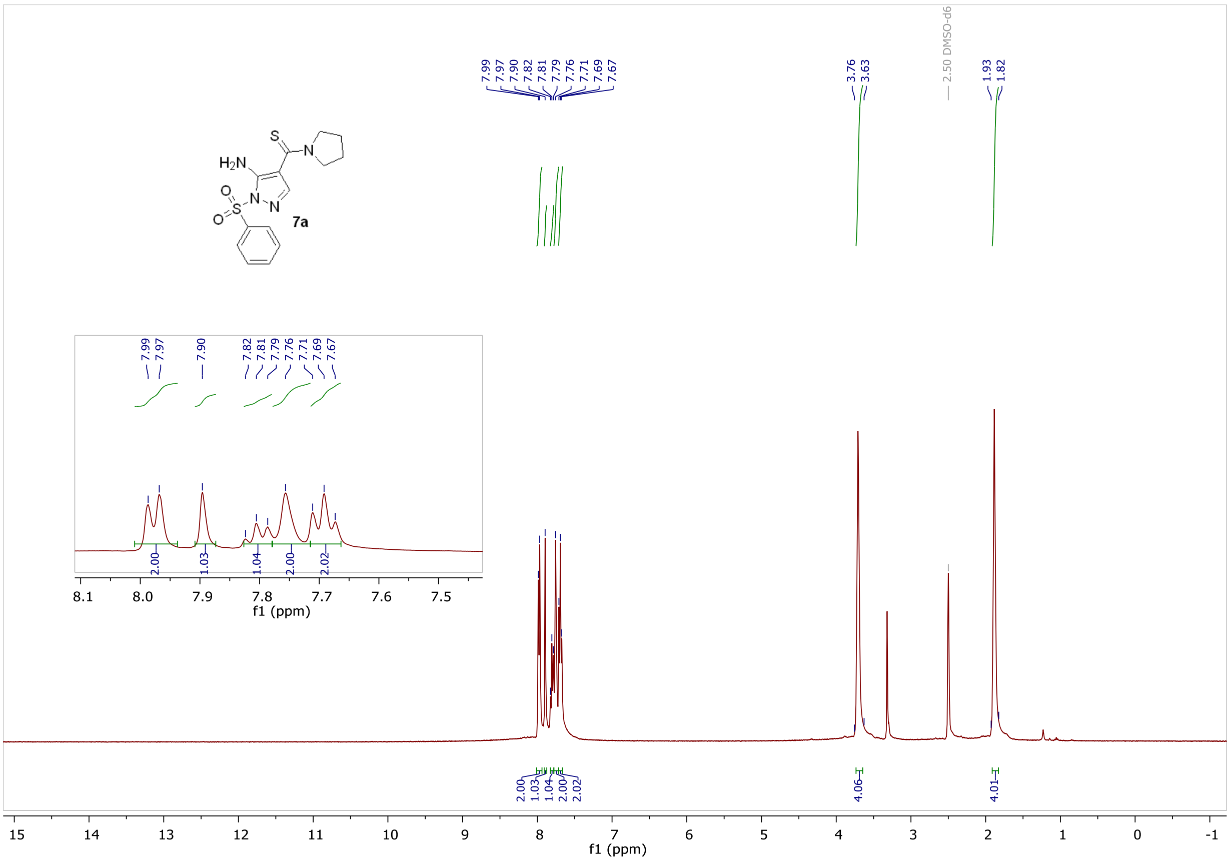

Figure S37. ^{13}C NMR spectrum (DMSO-d_6) of **7a**.

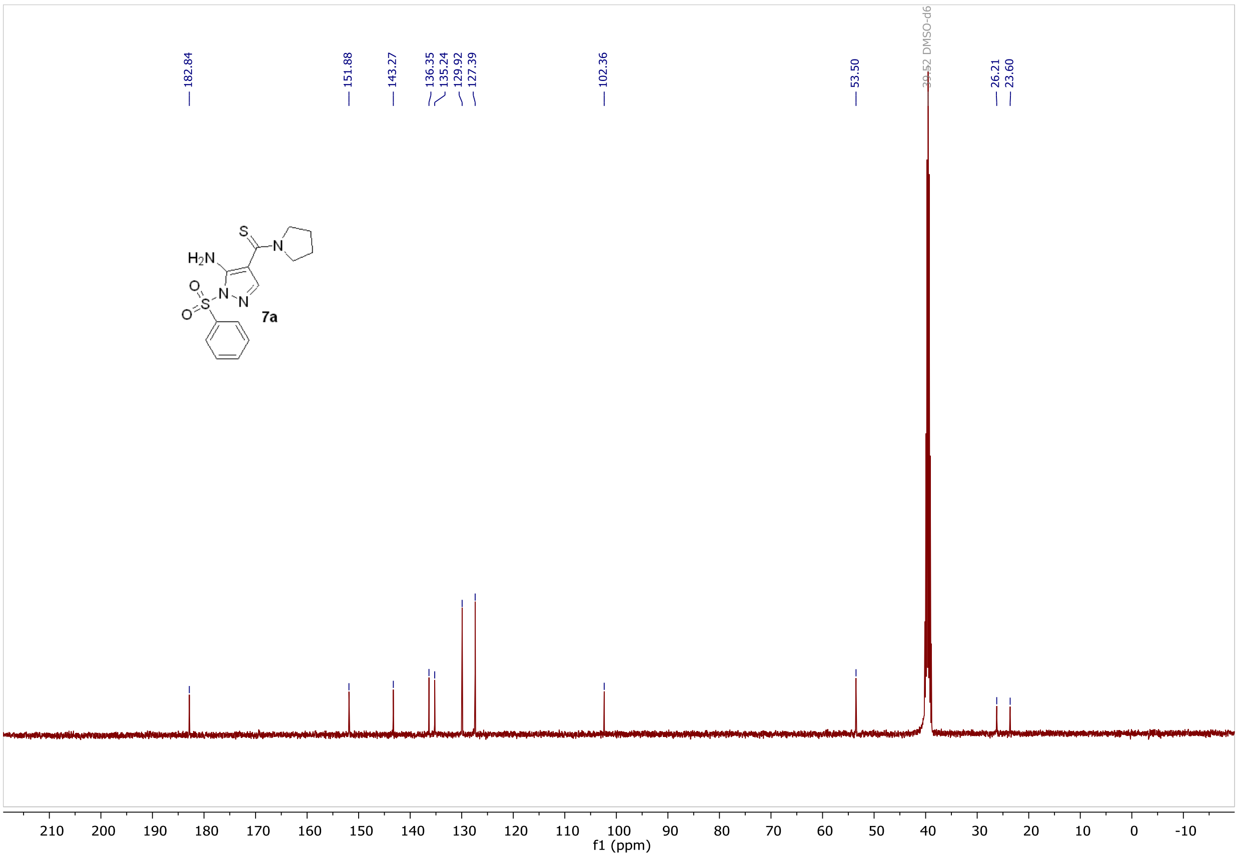

Figure S38. ^{1}H NMR spectrum (DMSO-d_6) of **7b**.

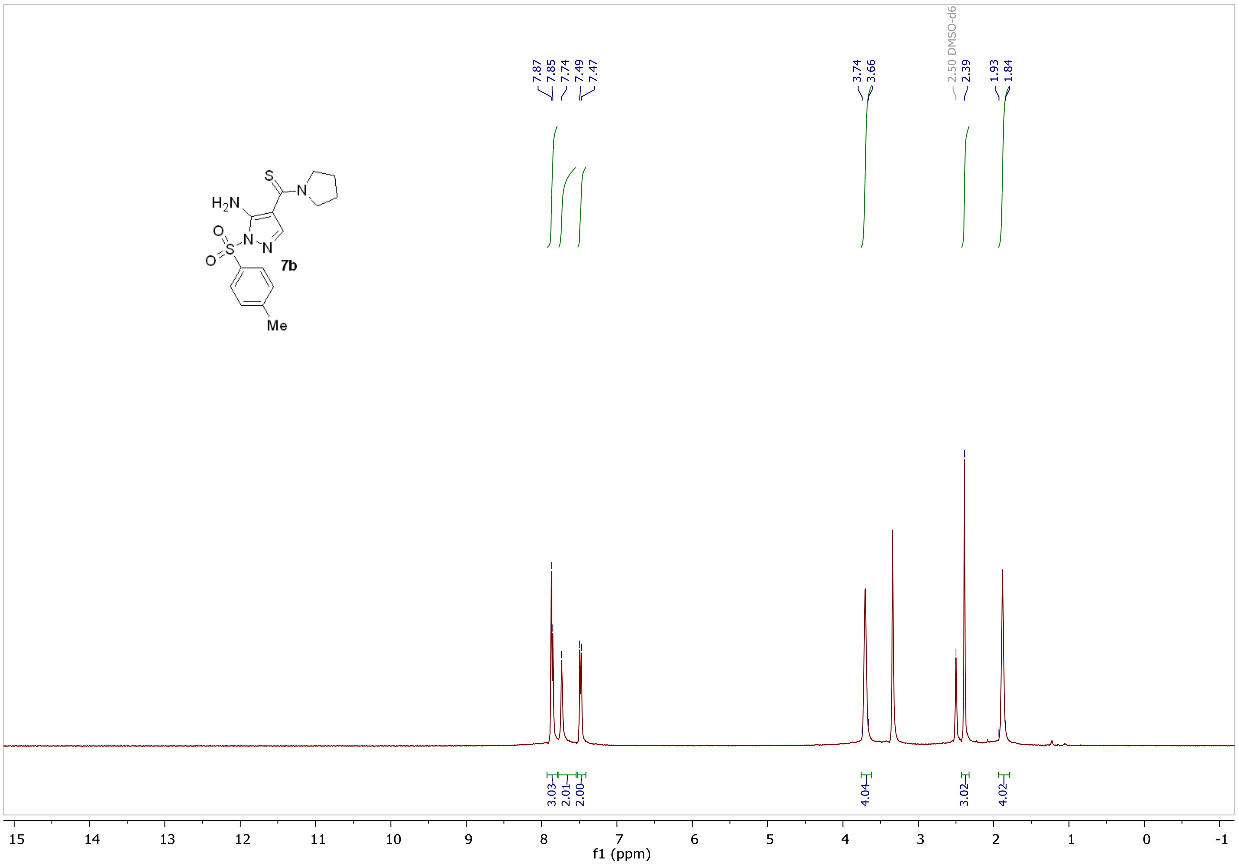

Figure S39. ^{13}C NMR spectrum (DMSO-d_6) of **7b**.

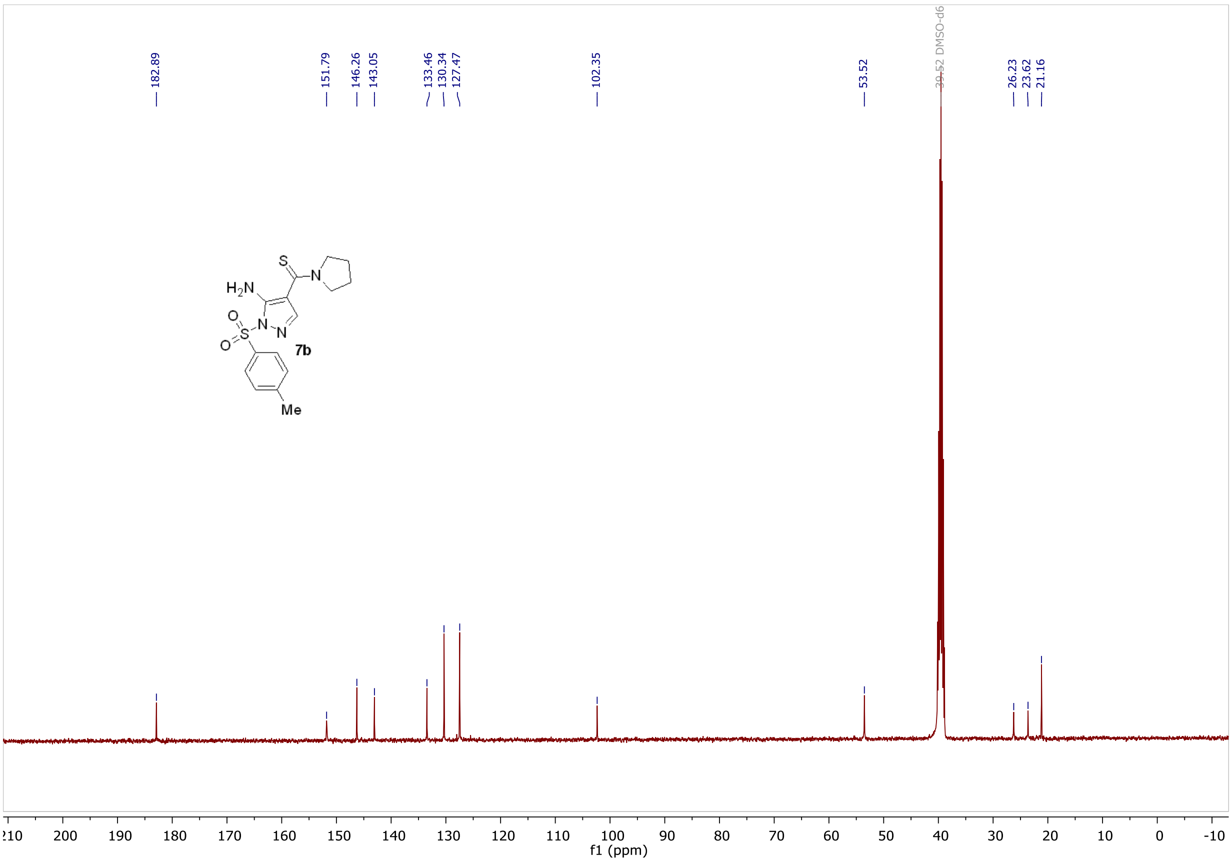

Figure S40. ^{1}H NMR spectrum (DMSO-d_6) of **7c**.

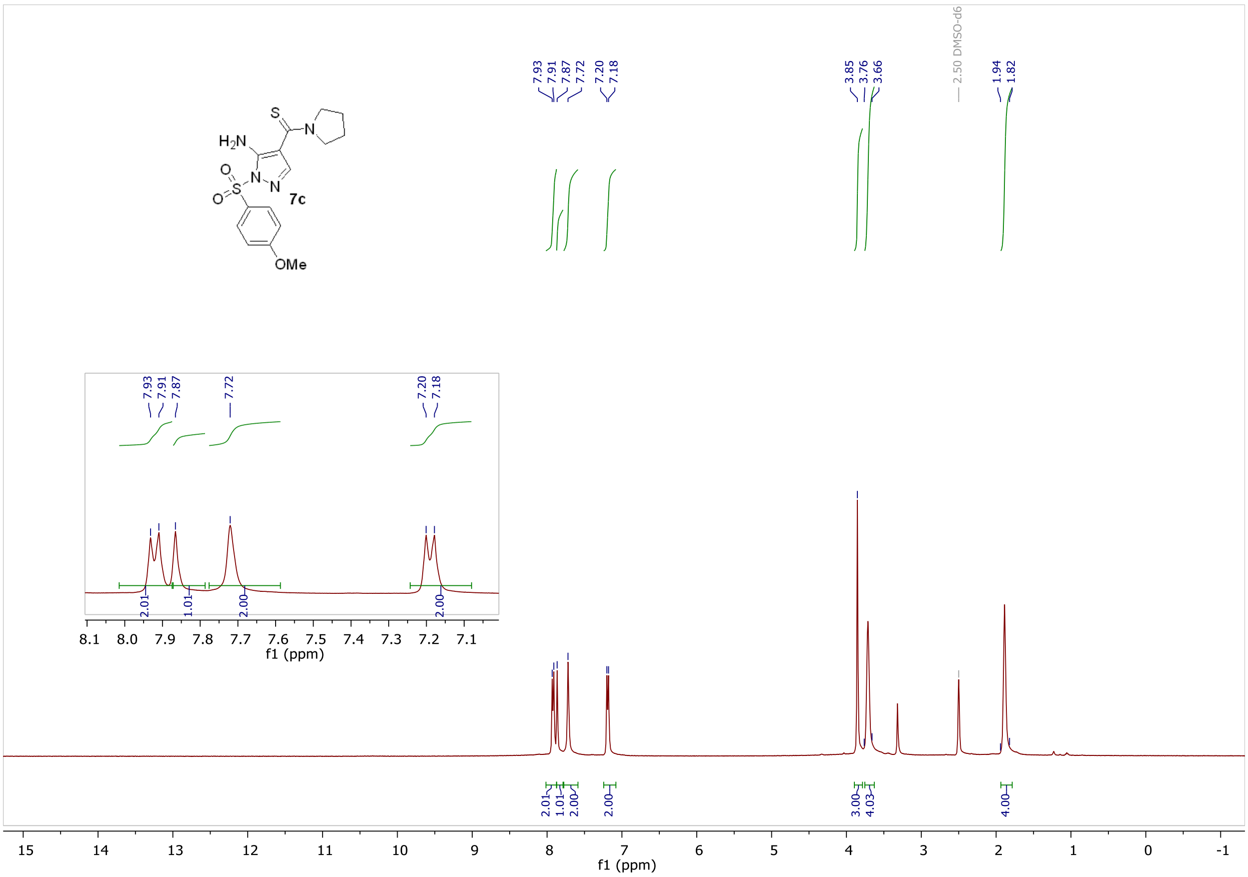

Figure S41. ^{13}C NMR spectrum (DMSO-d_6) of **7c**.

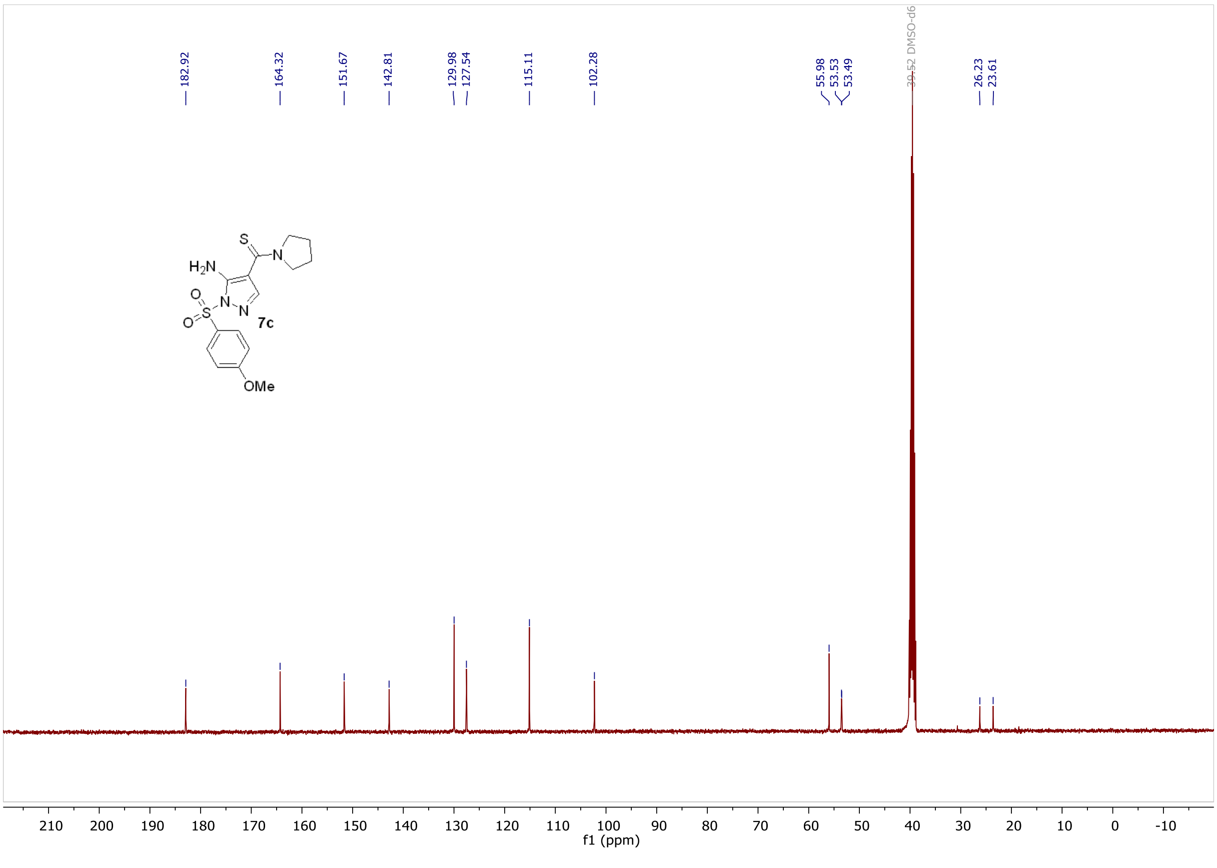

Figure S42. ^{1}H NMR spectrum (DMSO-d_6) of **7d**.

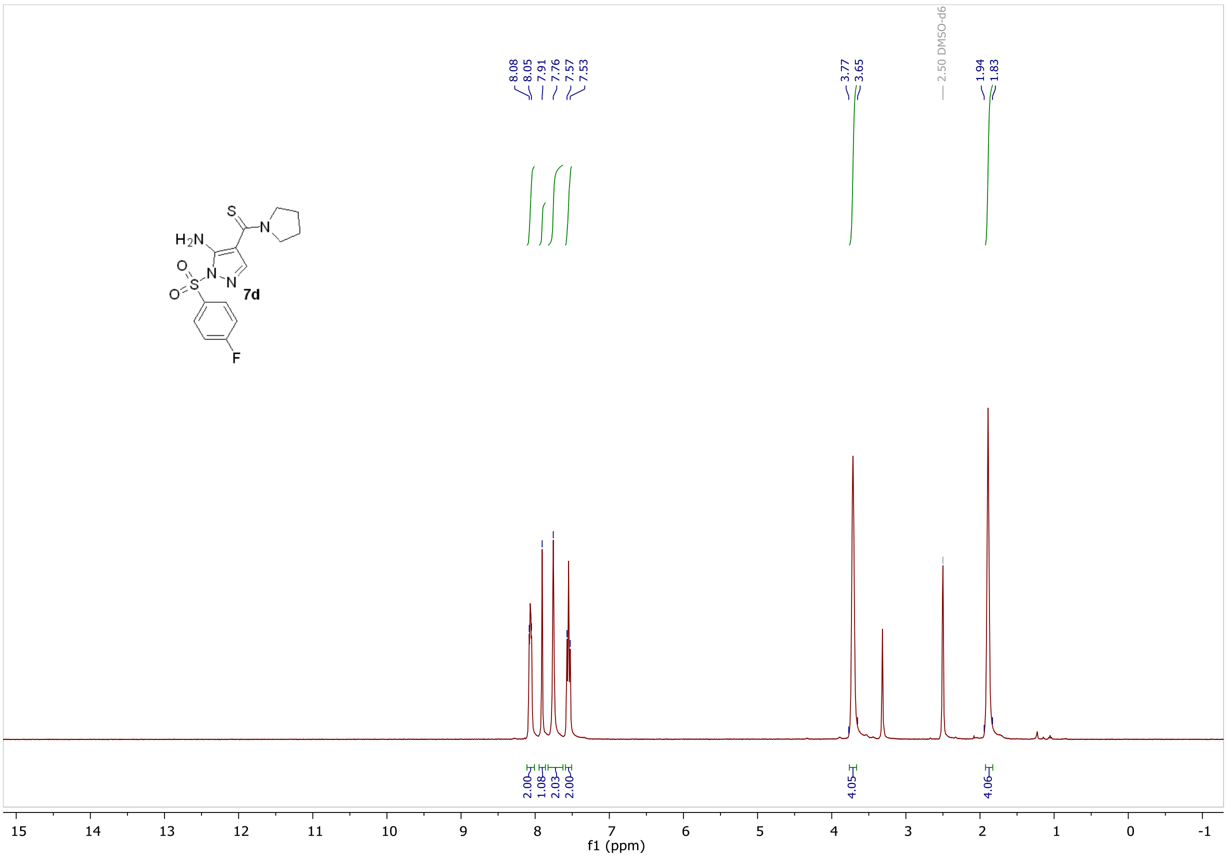

Figure S43. ^{13}C NMR spectrum (DMSO-d_6) of **7d**.

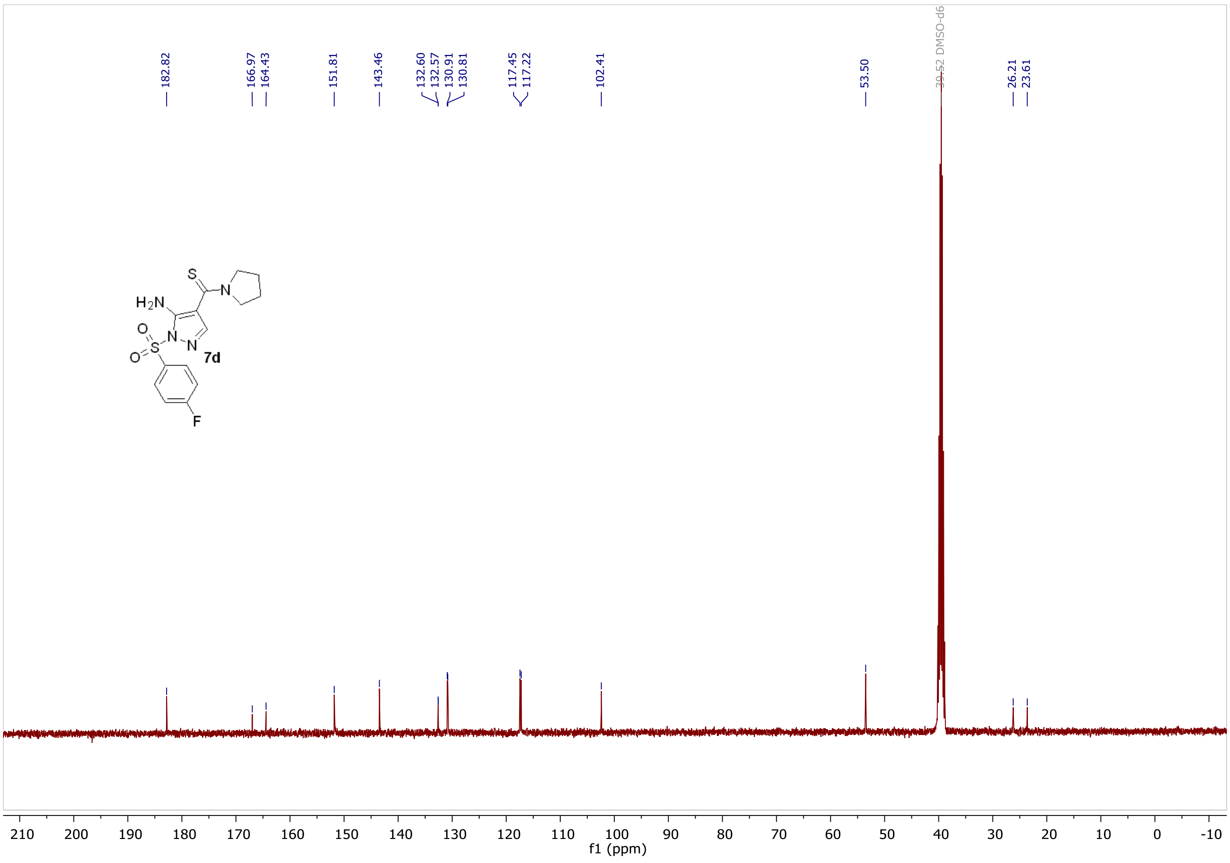

Figure S44. ^{19}F NMR spectrum (DMSO-d_6) of **7d**.

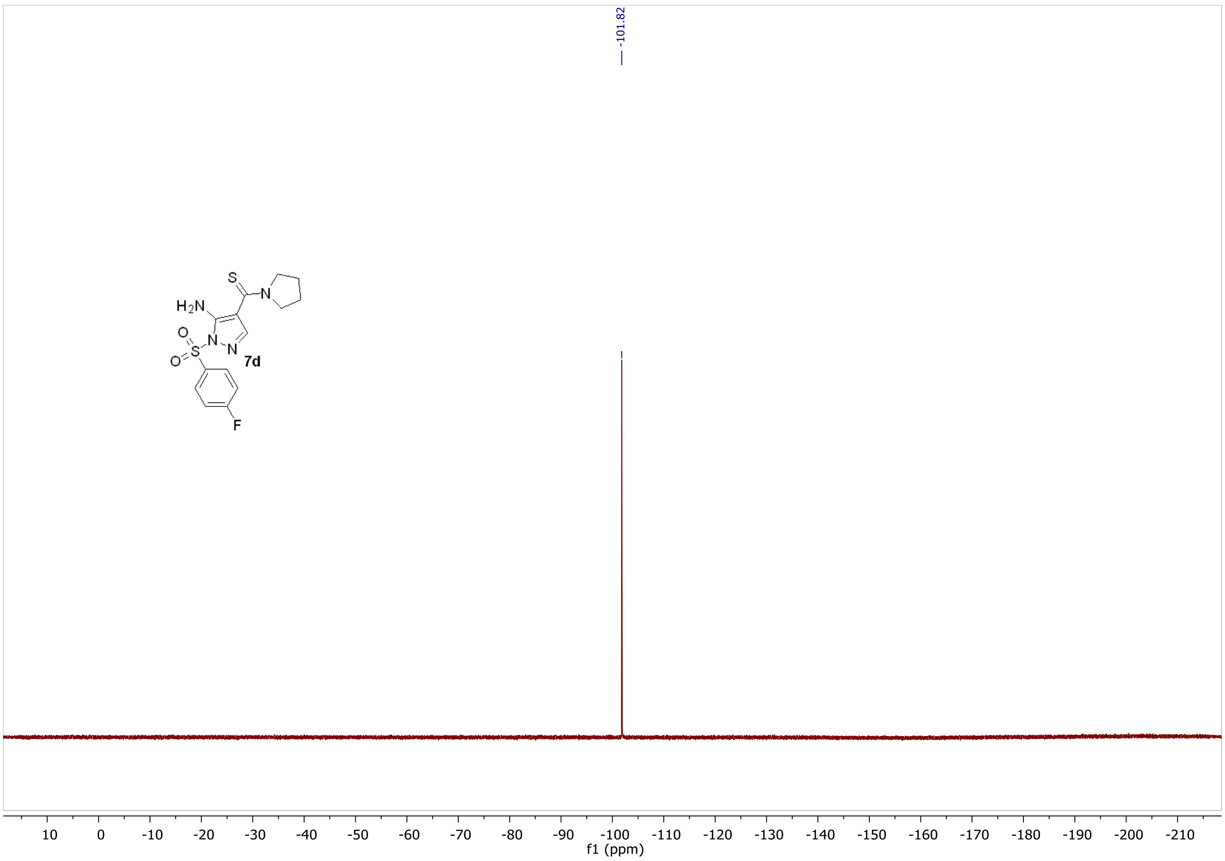

Figure S45. ^{1}H NMR spectrum (DMSO-d_6) of **7e**.

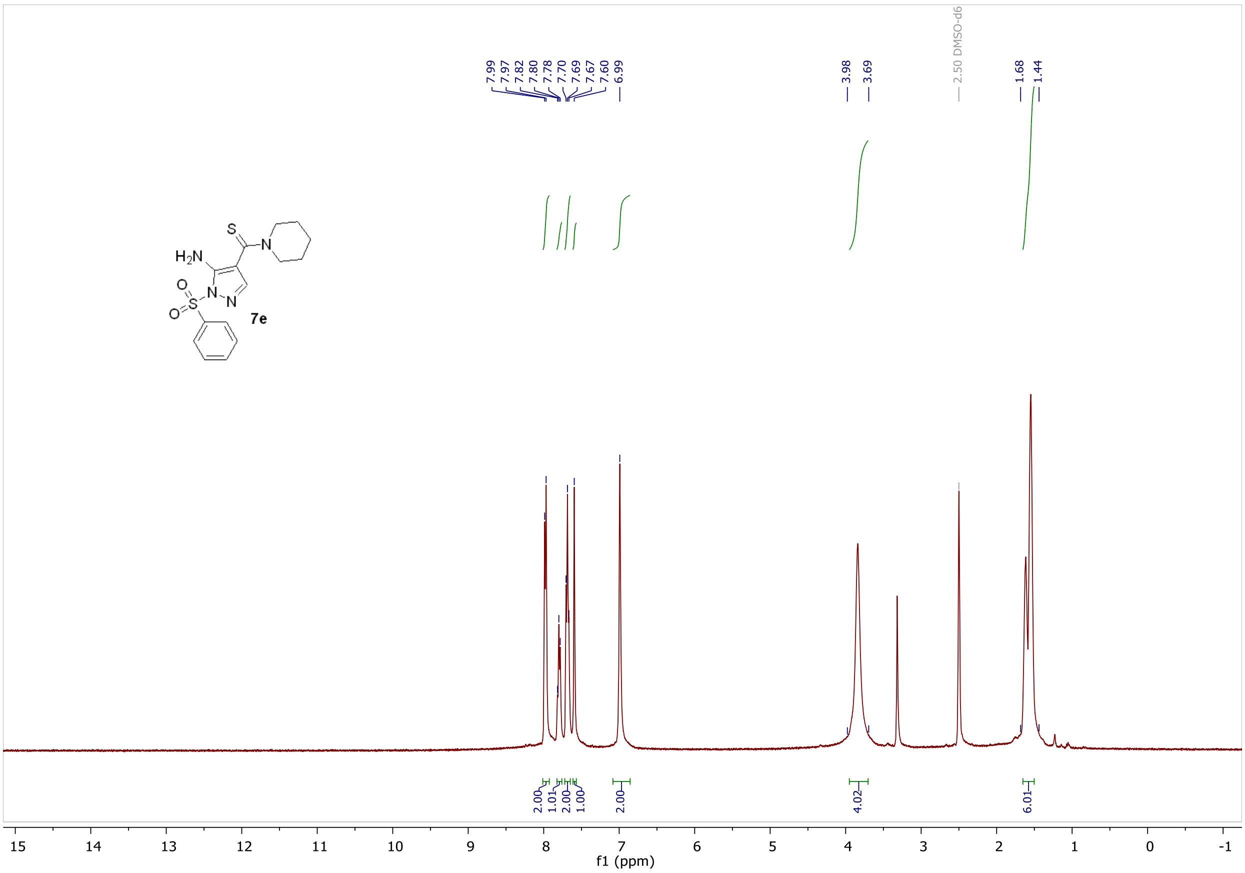

Figure S46. ^{13}C NMR spectrum (DMSO-d_6) of **7e**.

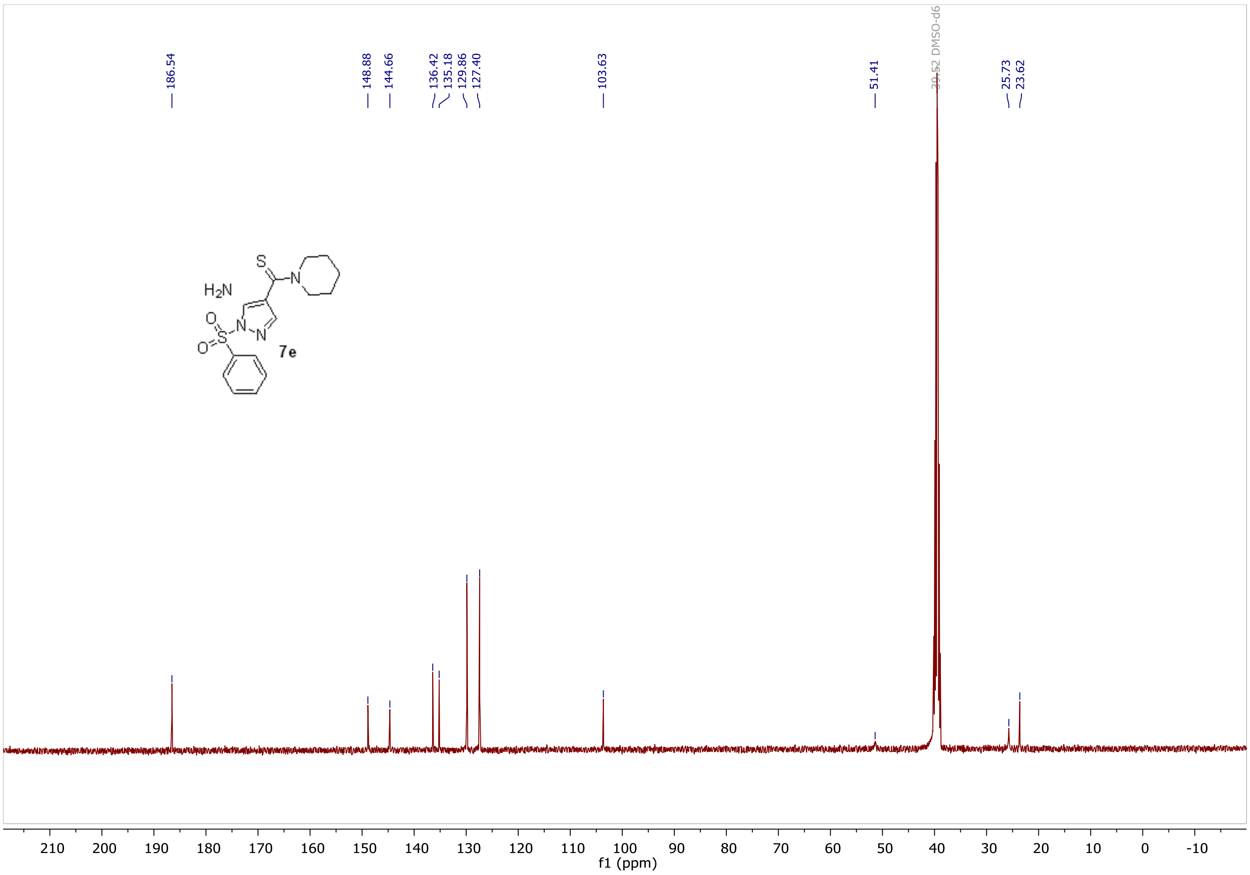

Figure S47. ^{1}H NMR spectrum (DMSO-d_6) of **7f**.

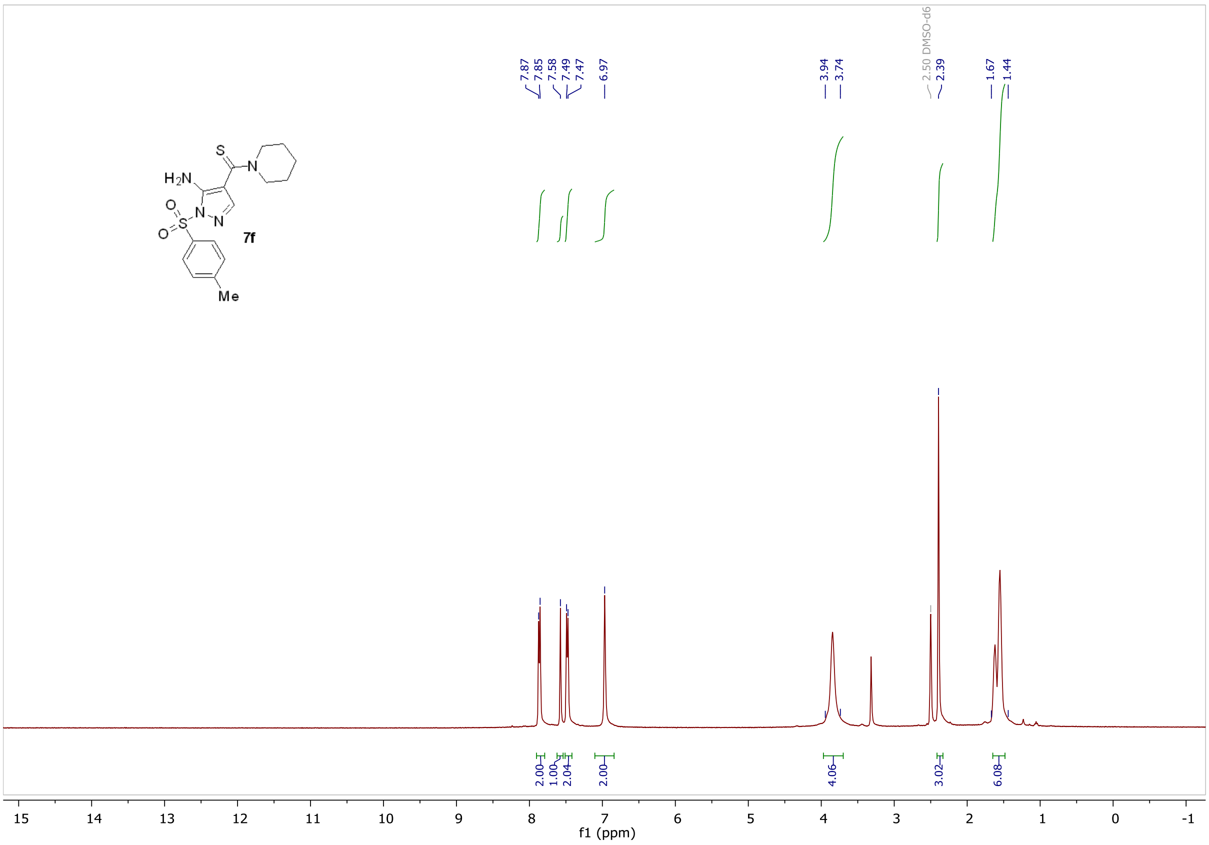

Figure S48. ^{13}C NMR spectrum (DMSO-d_6) of **7f**.

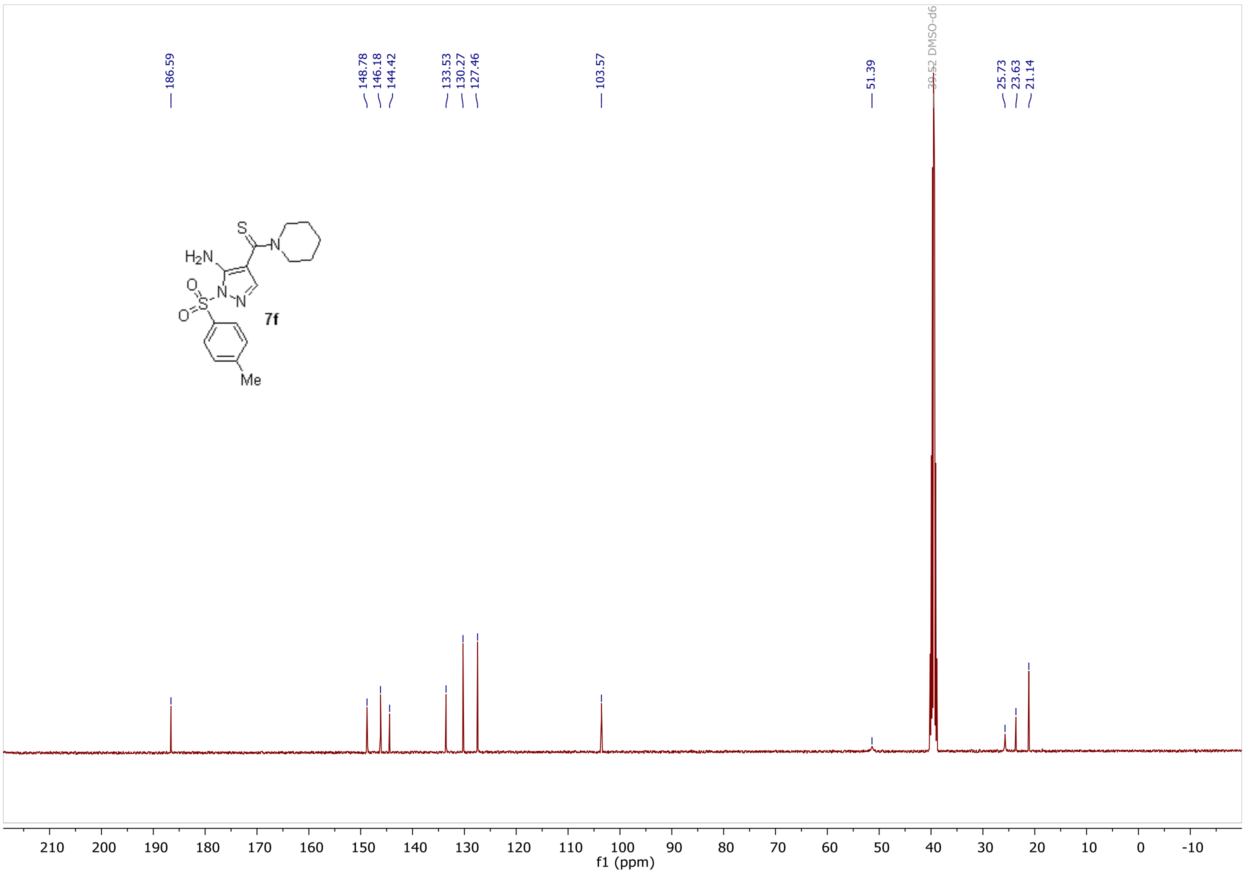

Figure S49. ^{1}H NMR spectrum (DMSO-d_6) of **7g**.

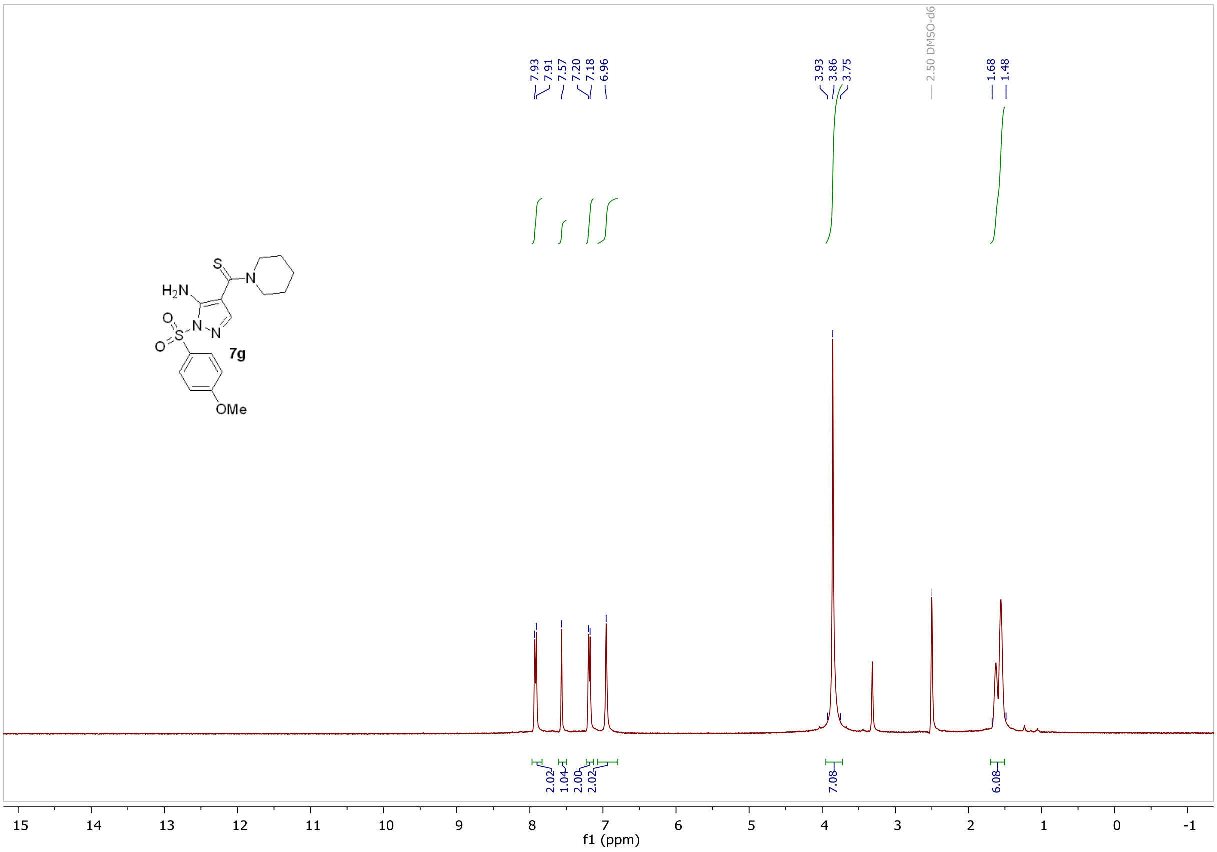

Figure S50. ^{13}C NMR spectrum (DMSO-d_6) of **7g**.

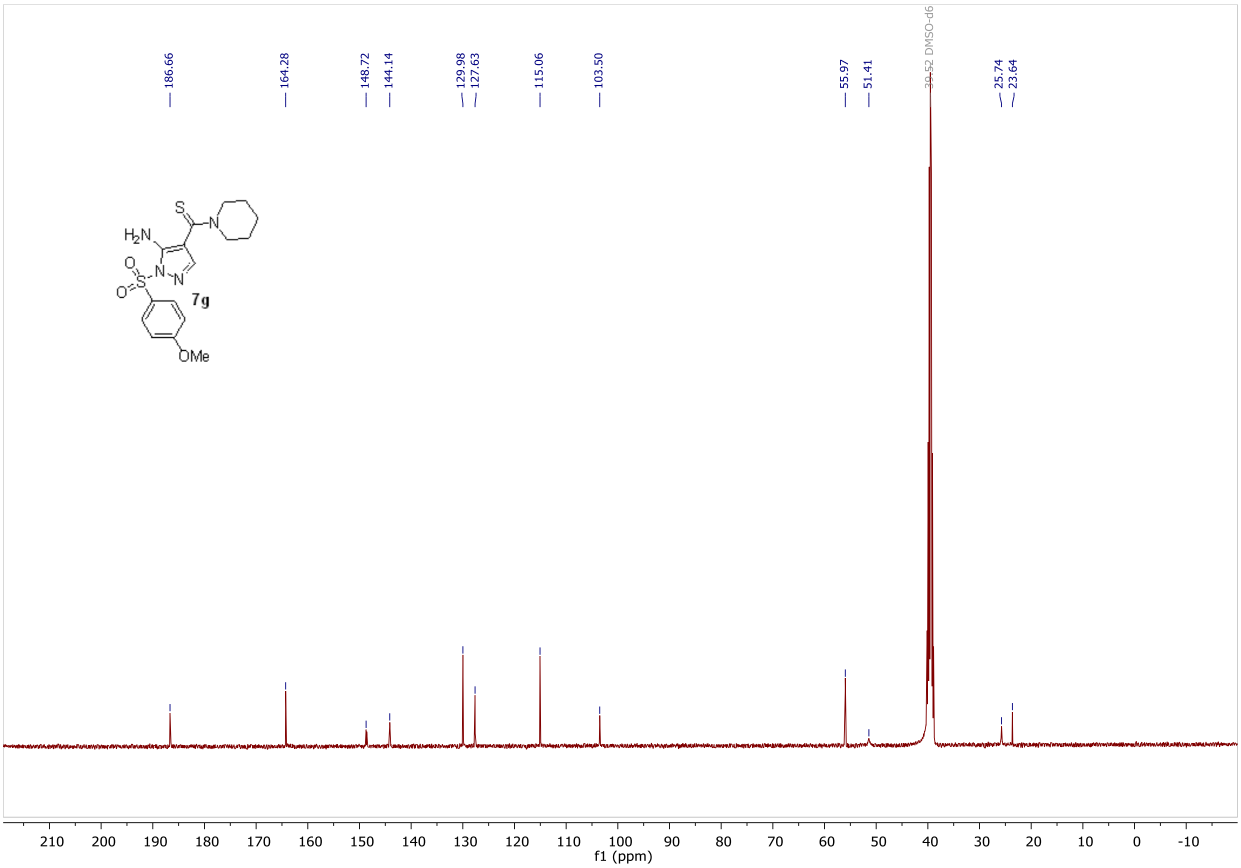

Figure S51. ^{1}H NMR spectrum (DMSO-d_6) of **7h**.

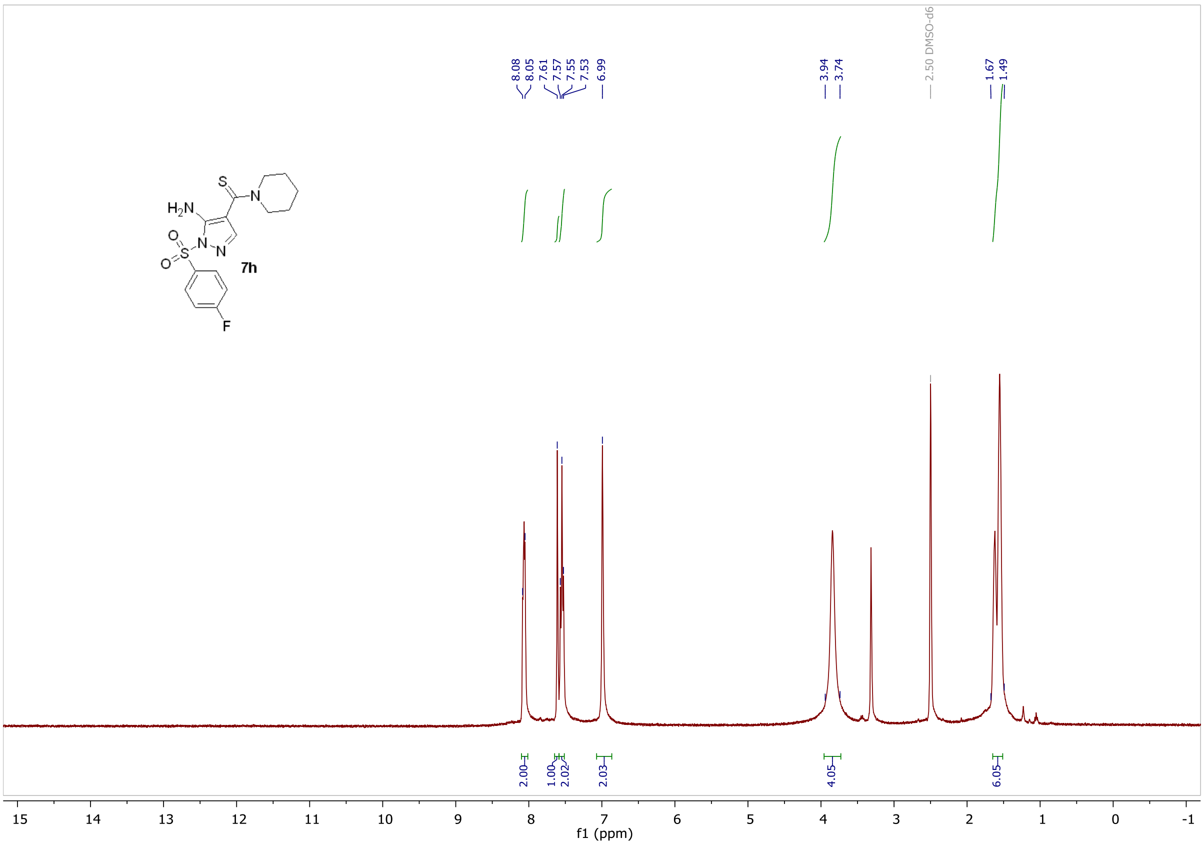

Figure S52. ^{13}C NMR spectrum (DMSO-d_6) of **7h**.

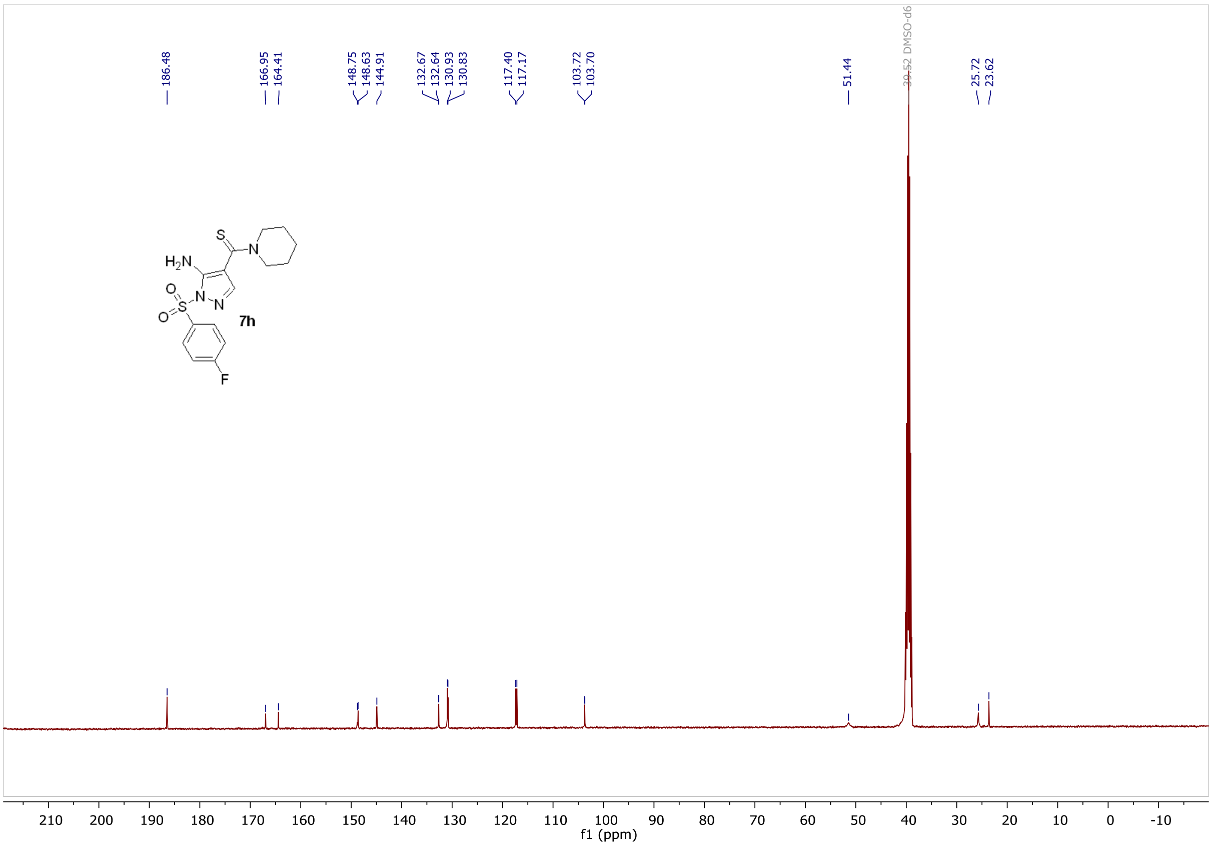

Figure S53. ^{1}H NMR spectrum (DMSO-d_6) of **7i**.

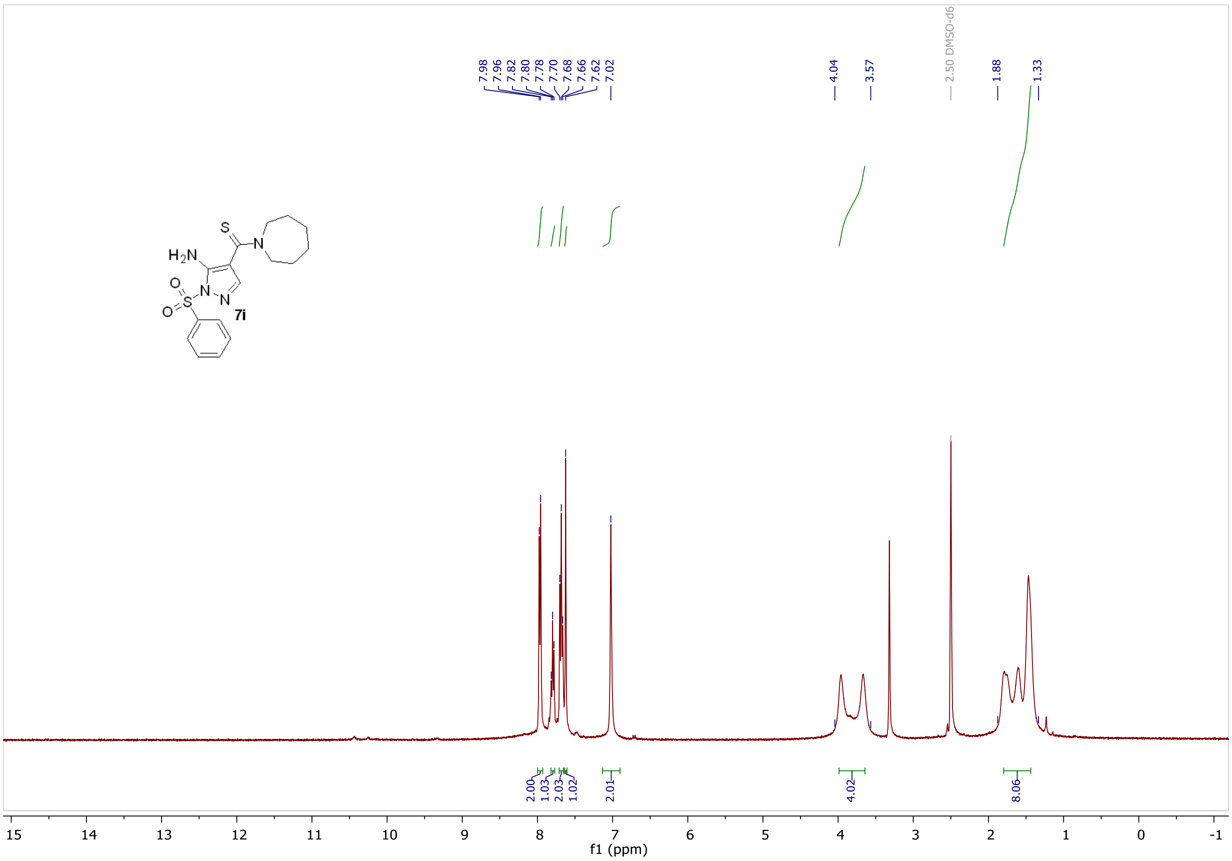

Figure S54. ^{13}C NMR spectrum (DMSO-d_6) of **7i**.

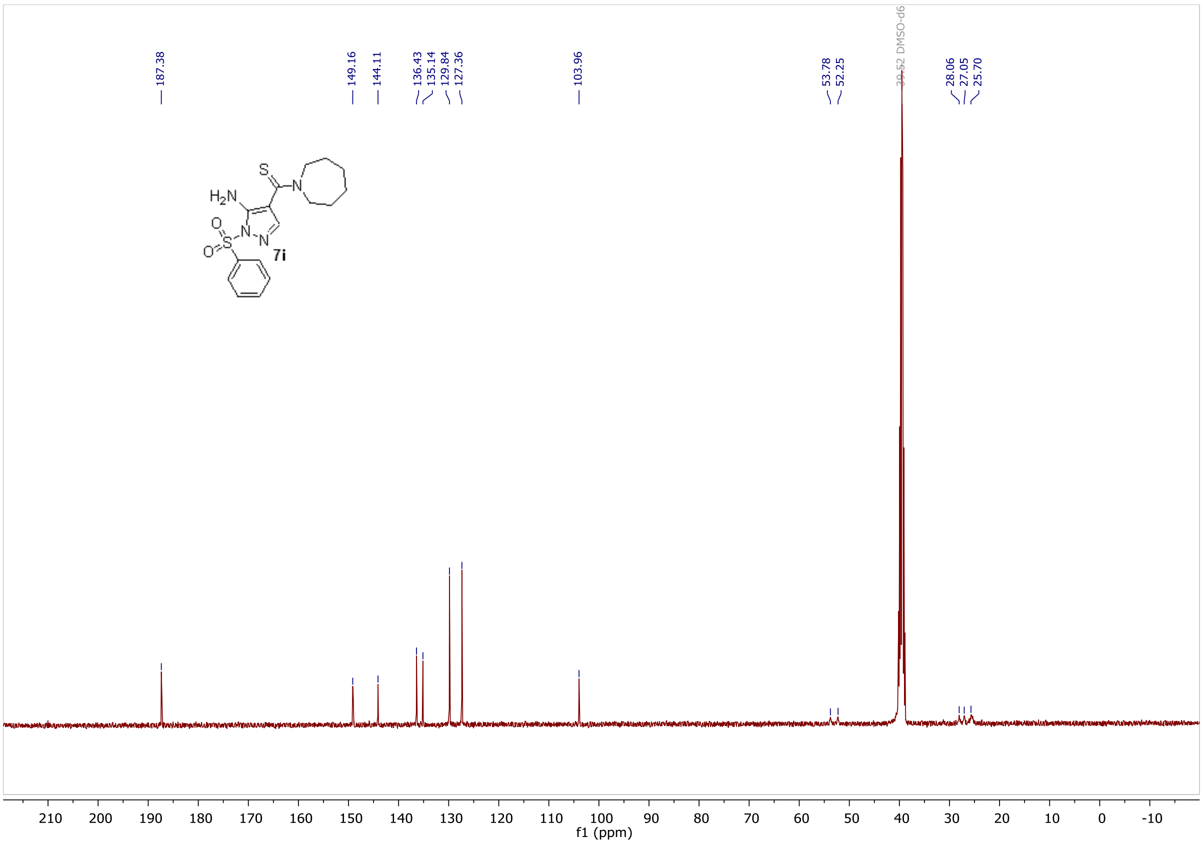

Figure S55. ^{1}H NMR spectrum (DMSO-d_6) of **7j**.

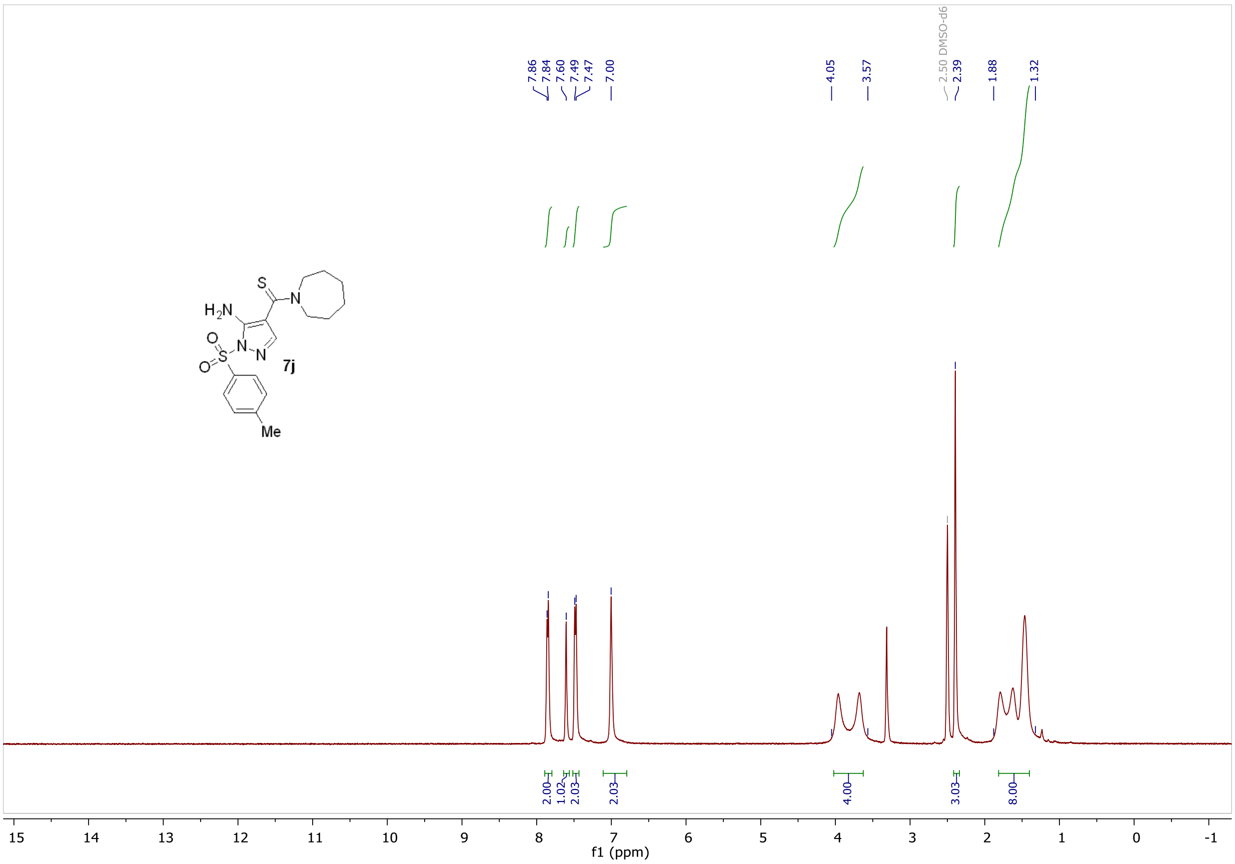

Figure S56. ^{13}C NMR spectrum (DMSO-d_6) of **7j**.

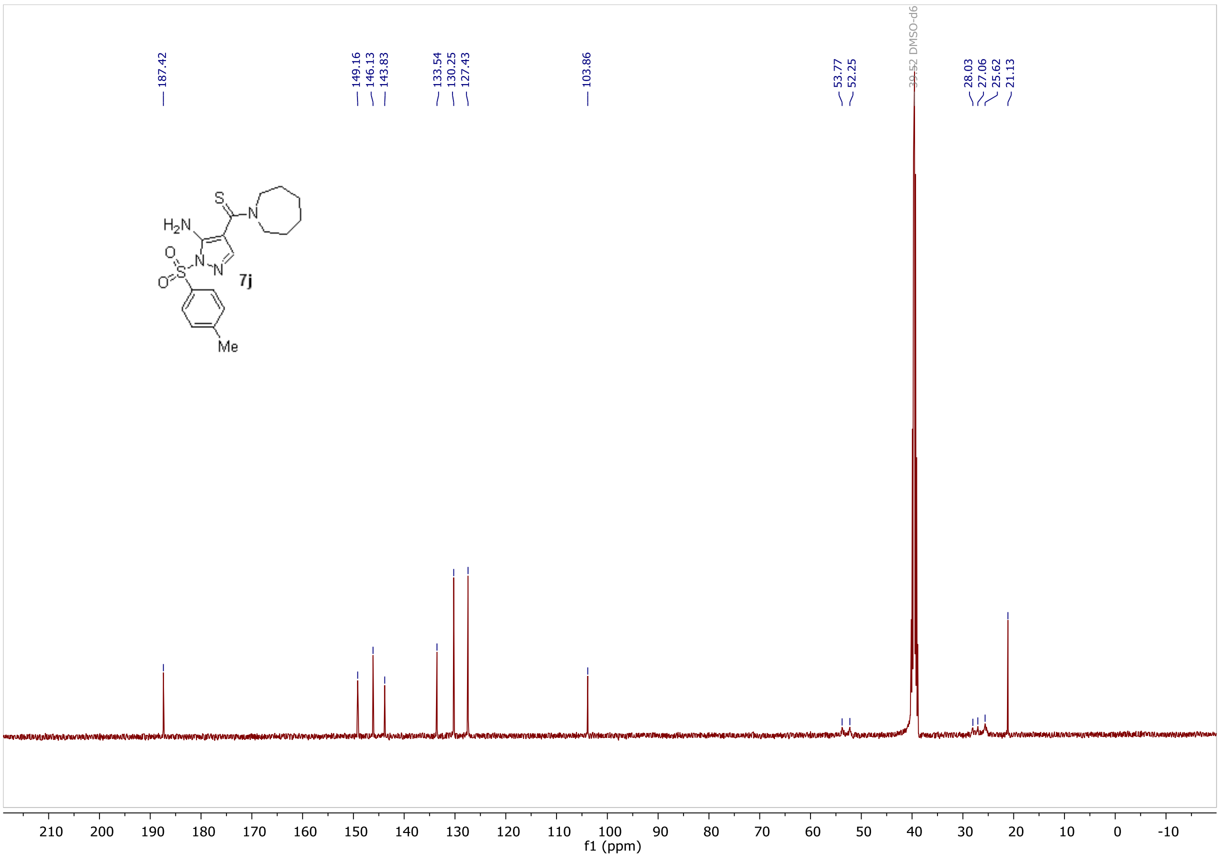

Figure S57. ^{1}H NMR spectrum (DMSO-d_6) of **7k**.

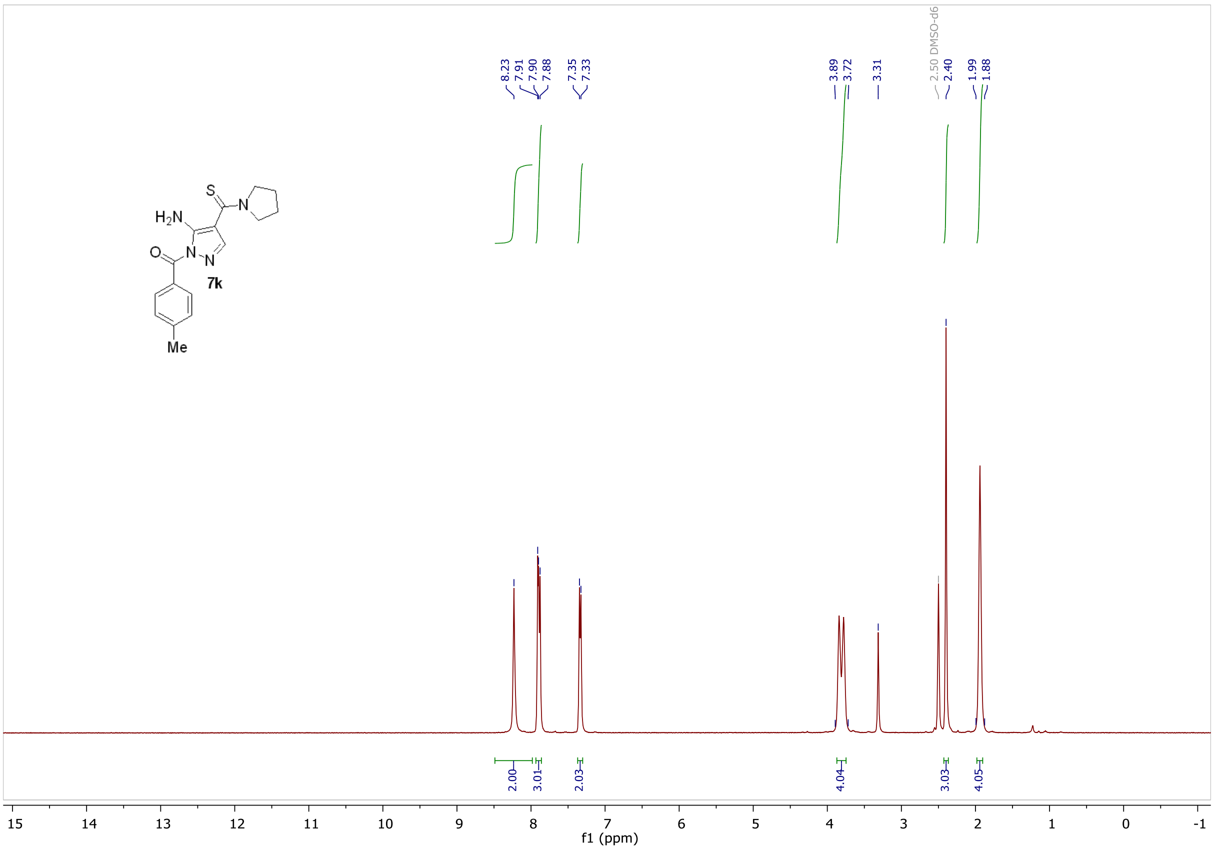

Figure S58. ^{13}C NMR spectrum (DMSO-d_6) of **7k**.

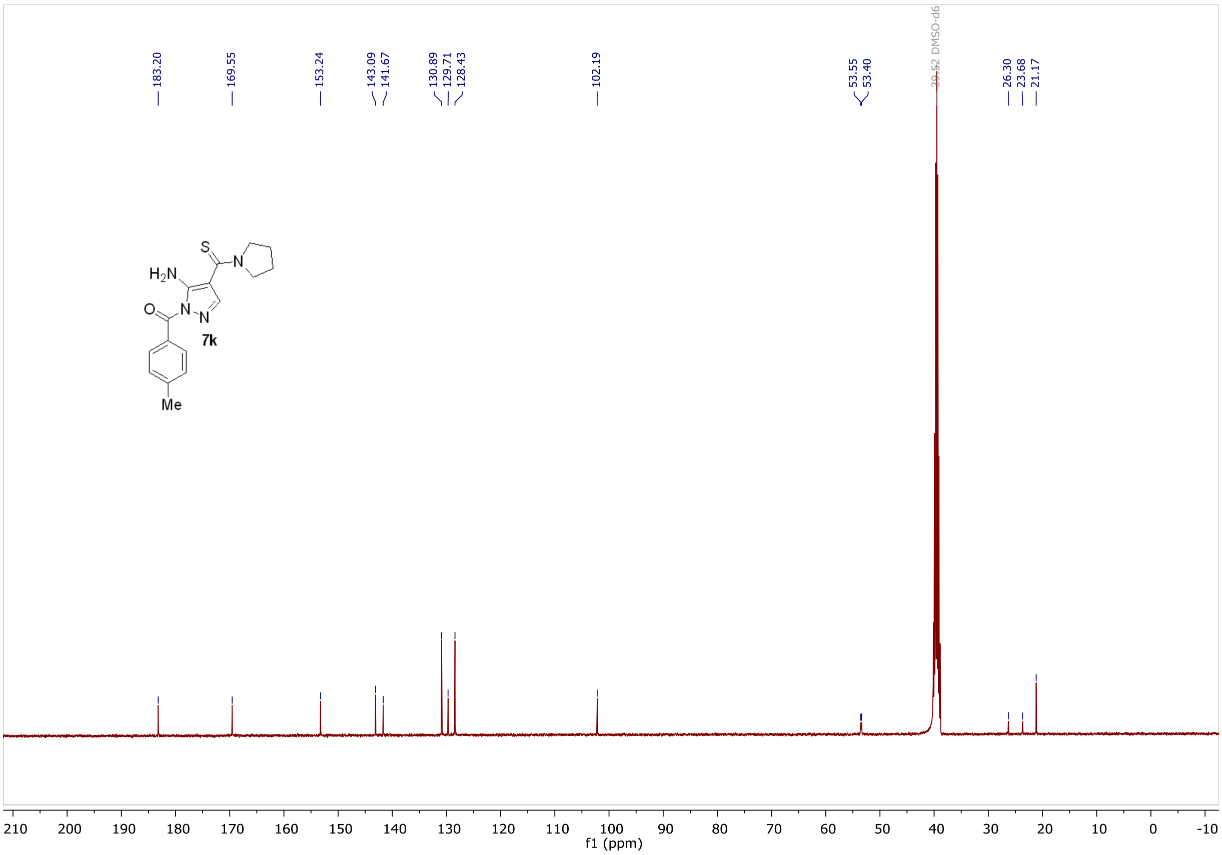

Supplement: File 2 — Copies of NMR spectra of all new compounds. [file Beilstein_J_Org_Chem-19-1191-s002.pdf]
